# Decoding the differentiation of mesenchymal stem cells into mesangial cells at the transcriptomic level

**DOI:** 10.1186/s12864-020-06868-5

**Published:** 2020-07-07

**Authors:** Chee-Yin Wong, Yao-Ming Chang, Yu-Shuen Tsai, Wailap Victor Ng, Soon-Keng Cheong, Ting-Yu Chang, I-Fang Chung, Yang-Mooi Lim

**Affiliations:** 1 Department of Pre-Clinical Sciences, Faculty of Medicine and Health Sciences, Universiti Tunku Abdul Rahman, Jalan Sungai Long, Bandar Sungai Long, 43000 Kajang, Selangor Malaysia; 2 Institute of Biomedical Sciences, Academia Sinica, 128, Academia Road, Section 2, Nankang, Taipei, Taiwan; 3 Center for Systems and Synthetic Biology, National Yang-Ming University, No. 155, Section 2, Linong Street, Taipei, Taiwan; 4 Department of Biotechnology and Laboratory Science in Medicine, National Yang-Ming University, No. 155, Section 2, Linong Street, Taipei, Taiwan; 5 Department of Research, ChangHua Christian Hospital, 135, Nan-Hsiao Street, ChangHua City, Taiwan; 6 Institute of Biomedical Informatics, National Yang-Ming University, No. 155, Section 2, Linong Street, Taipei, Taiwan; 7 Preventive Medicine Research Center, National Yang-Ming University, No. 155, Section 2, Linong Street, Taipei, Taiwan

**Keywords:** Mesenchymal stem cell, Mesangial cell, Differentiation, Monotonic feature selector, Time-ordered gene co-expression network, Transcriptomic

## Abstract

**Background:**

Mesangial cells play an important role in the glomerulus to provide mechanical support and maintaine efficient ultrafiltration of renal plasma. Loss of mesangial cells due to pathologic conditions may lead to impaired renal function. Mesenchymal stem cells (MSC) can differentiate into many cell types, including mesangial cells. However transcriptomic profiling during MSC differentiation into mesangial cells had not been studied yet. The aim of this study is to examine the pattern of transcriptomic changes during MSC differentiation into mesangial cells, to understand the involvement of transcription factor (TF) along the differentiation process, and finally to elucidate the relationship among TF-TF and TF-key gene or biomarkers during the differentiation of MSC into mesangial cells.

**Results:**

**S**everal ascending and descending monotonic key genes were identified by Monotonic Feature Selector. The identified descending monotonic key genes are related to stemness or regulation of cell cycle while ascending monotonic key genes are associated with the functions of mesangial cells. The TFs were arranged in a co-expression network in order of time by Time-Ordered Gene Co-expression Network (TO-GCN) analysis. TO-GCN analysis can classify the differentiation process into three stages: differentiation preparation, differentiation initiation and maturation. Furthermore, it can also explore TF-TF-key genes regulatory relationships in the muscle contraction process.

**Conclusions:**

A systematic analysis for transcriptomic profiling of MSC differentiation into mesangial cells has been established. Key genes or biomarkers, TFs and pathways involved in differentiation of MSC-mesangial cells have been identified and the related biological implications have been discussed. Finally, we further elucidated for the first time the three main stages of mesangial cell differentiation, and the regulatory relationships between TF-TF-key genes involved in the muscle contraction process. Through this study, we have increased fundamental understanding of the gene transcripts during the differentiation of MSC into mesangial cells.

## Background

The mesangial cell, also called modified smooth muscle cell, plays an important role in the glomerulus as these cells and their mesangial extracellular matrix constitute the glomerular mesangium and provide mechanical support to the glomerulus [1]. Mesangial cells have the characteristics of specialized renal pericytes and are also capable of a number of other functions. Their contractile properties enable mesangial cells to alter intraglomerular capillary flow and glomerular ultrafiltration surface [2]. These cells also perform phagocytosis and endocytosis in which these cells take up residues such as ferritin, colloidal carbon and aggregated proteins [3]. Loss of mesangial cells due to pathologic conditions such as glomerulonephritis or diabetic nephropathy may lead to impaired renal function.

Adult human bone marrow contains mesenchymal stem cells (MSC) that can differentiate into many cell types such as chondrocytes, osteocytes, neurons, adipocytes and renal component cells like mesangial cells, epithelial cells and endothelial cells [4–6]. Many researchers have reported on the gene expression or transcriptomic profiling during MSC differentiation into terminal cells such as chondrocytes [7], osteocytes [8], and neurons [9]. However, the gene expression or transcriptomic profiling during MSC differentiation into mesangial cells has not been studied yet.

Each terminal cell type has a specific regulated gene expression [10]. Stem cell differentiation is determined by the underlying gene regulatory network during the process of development, which leads the stem cells into their particular terminal cell phenotype. During stem cell differentiation, the stemness biomarkers of stem cells will descend over time while the characteristics and functions of terminal cells will ascend [11].

Based on such specific gene profiles mentioned above, this study was carried out to study the pattern of transcriptomic changes during MSC differentiation into mesangial cells and to identify biomarkers or key genes involved in this differentiation. Additionally, we sought to understand and identify the involvement of transcription factors (TFs) along the differentiation process, and finally to elucidate the relationship among TF-TF and TF-key genes during MSC-mesangial cell differentiation. To achieve these objectives, we used two methods that were developed in-house, Monotonic Feature Selector (MFSelector) [12] and Time-Ordered Gene Co-expression Network (TO-GCN) [13]. MFSelector was used to identify the key genes with descending monotonic patterns (MSC related stemness key genes or biomarkers) or ascending monotonic patterns (i.e. terminal cell related characteristics and functions) during the differentiation process. Meanwhile TO-GCN was used to construct a TF co-expression network and to determine TF gene expression by order of time. These two methods work synergistically to build a transcriptomic profile with identified key genes or biomarkers involved in the differentiation pathway and provide a deeper fundamental understanding of MSC differentiation into mesangial cells.

## Results

### Batch normalization and monotonic expression patterns

In this study, three biological replicates (*n* = 3) were co-cultured in two batches. Thereafter, batch normalization was carried out. Multidimensional scaling (MDS) plot (Additional file 1A) and heatmap (Additional file 1B) were generated to show the effect of the batch adjustment and monotonic gene expression across the samples at different days respectively.

### Statistics of genes after initial read processing

**Fig. 1:**
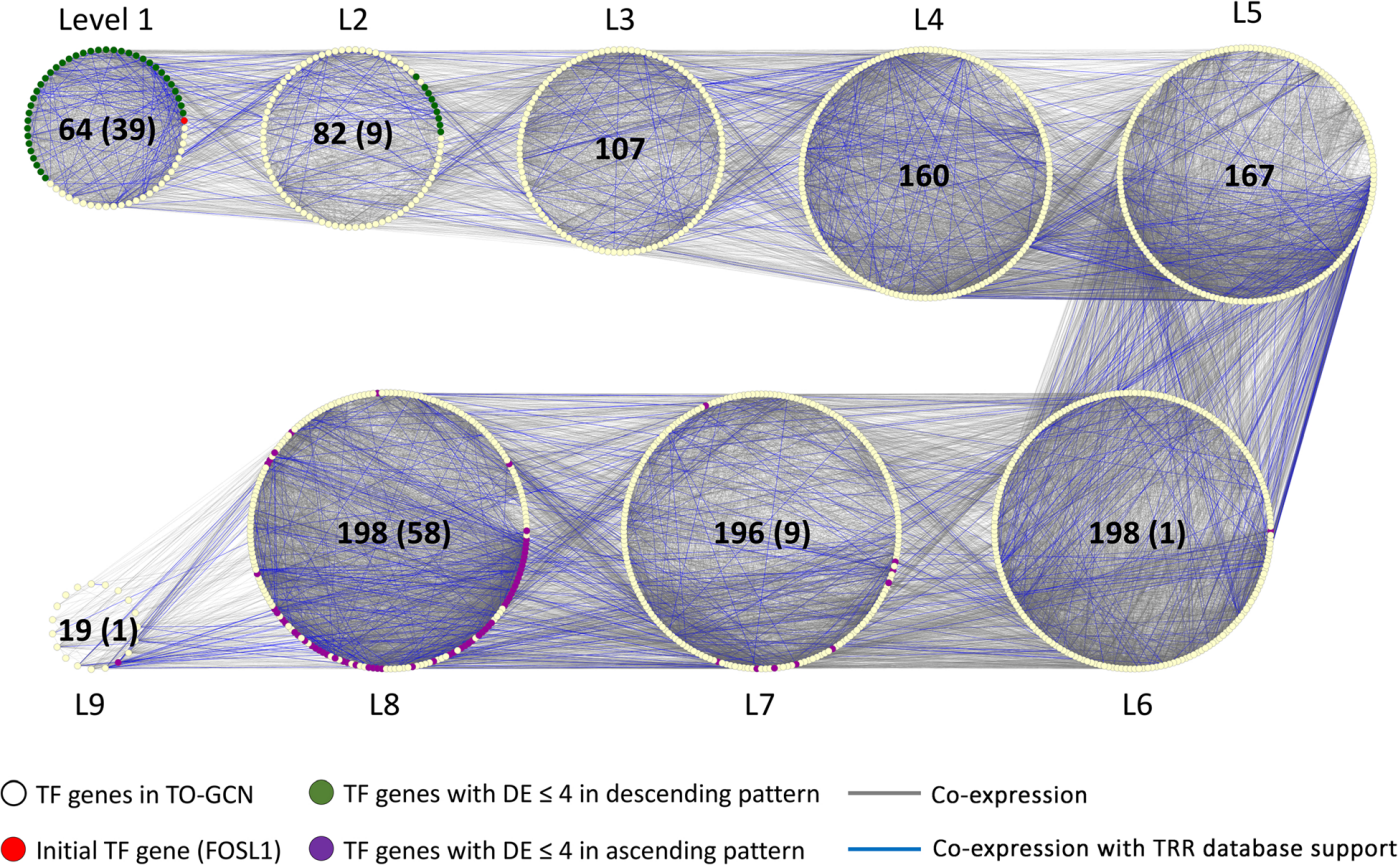
TO-GCN with 9 levels was constructed from 1191 TF genes. *FOSL1*, a TF with the strongest monotonic descending pattern (DE = 0), was used as initial TF gene. All TFs were linked together by co-expression relationship (gray line), while some of the co-expression were reported with TRR database support (blue line). TF coloured with green and purple are the TF genes with DE ≤ 4 in descending and ascending pattern respectively. The numbers stated in the middle of each level represent the number of TFs, and TFs with DE ≤ 4 (in parentheses) for that particular level

After initial processing for RNA-seq data, 13,135 genes (including 1191 TFs) were selected for MFSelector and TO-GCN analysis. TO-GCN with 9 levels was constructed from these 1191 TF genes (Fig. 1) and a list of these TF genes was supplemented in Additional file 2.

In this study, genes with DE ≤ 4 were selected for further analysis. Among the 13,135 genes, 1026 genes (including 48 TFs) were found in a monotonic descending pattern with DE ≤ 4, while 927 genes (including 69 TFs) have a monotonic ascending pattern with DE ≤ 4. Those non-TF genes with DE ≤ 4 show a similar location (L1–2 and L7–9, respectively) of their upstream TFs in TO-GCN. A list of monotonic descending and ascending pattern genes with DE ≤ 4 was shown in Additional files 3 and 4 respectively.

### Evaluation of key genes and biomarkers of MSC down-regulated during the differentiation process

**Fig. 2:**
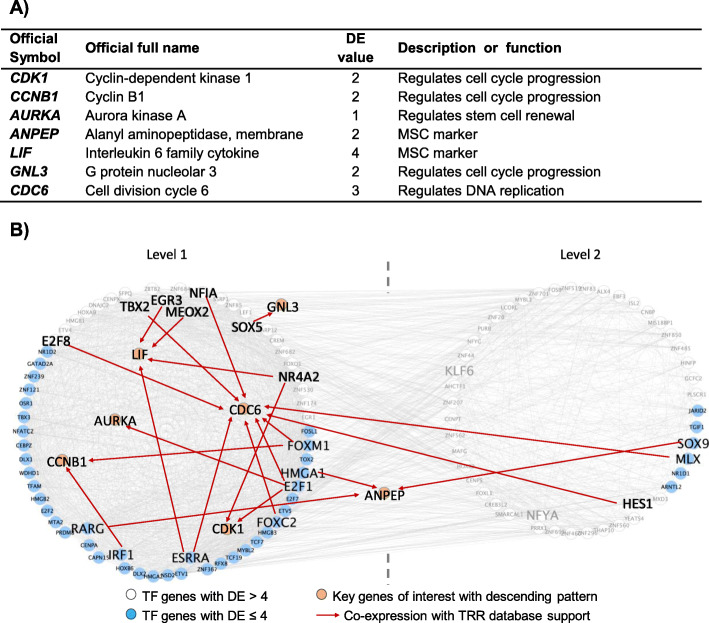
**a** Evaluation of key genes and biomarkers of MSC down-regulated during the differentiation process. **b** The connection of these selected descending key genes with their upstream regulators/TFs. These co-expression TFs and key genes were supported with TRR database

Seven descending pattern key genes with DE ≤ 4 were selected for further analysis (Fig. 2a). Three of these key genes were related to biomarkers of MSC (*ANPEP, LIF*) and stem cell renewal (*AURKA*). The remaining selected key genes with a descending-pattern during the differentiation process were related to cell cycle (*CDK1, CCNB1, GNL3*) or DNA replication (*CDC6*).

By searching the selected transcriptional regulatory relationship (TRR) databases, the upstream regulators or TFs for these key genes were identified and these TFs were located in between level (L) 1 and L2 in TO-GCN (Fig. 2b). *ANPEP* was regulated by TF *RARG, HMGA1* and *SOX9*, while TF *MEOX2* regulated *LIF*. TF *E2F1* regulated 3 key genes (*AURKA, CDK1* and *CDC6*). *CCNB1* was also being regulated by TF *IRF1* and *FOXM1.* Key gene *GNL3* was regulated by TF *SOX5*. Some of the TF-key gene relationships mentioned above were also found in ENCODE TF-Targets Dataset (Additional file 5 A).

### Key genes involved in the mesangial cell characteristic and function were up-regulated along the differentiation process

**Fig. 3:**
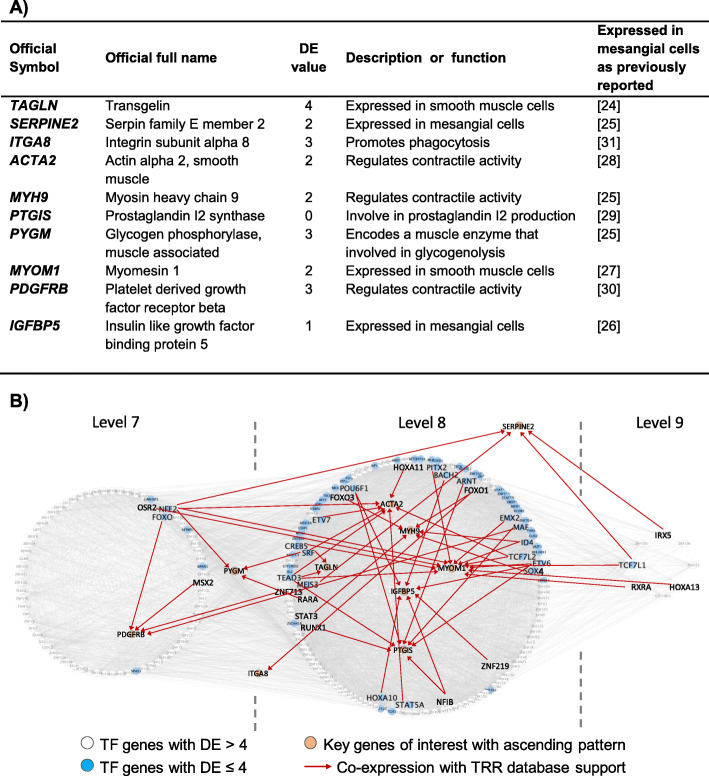
**a** Biomarkers or key genes involved in the mesangial cell characteristic and functions were up-regulated during the differentiation process. The expression of these selected key genes in mesangial cells are also being reported in other research. **b** The connection between these selected ascending key genes and their upstream regulators/TFs. These co-expression TFs and key genes were supported with TRR database

**Fig. 4:**
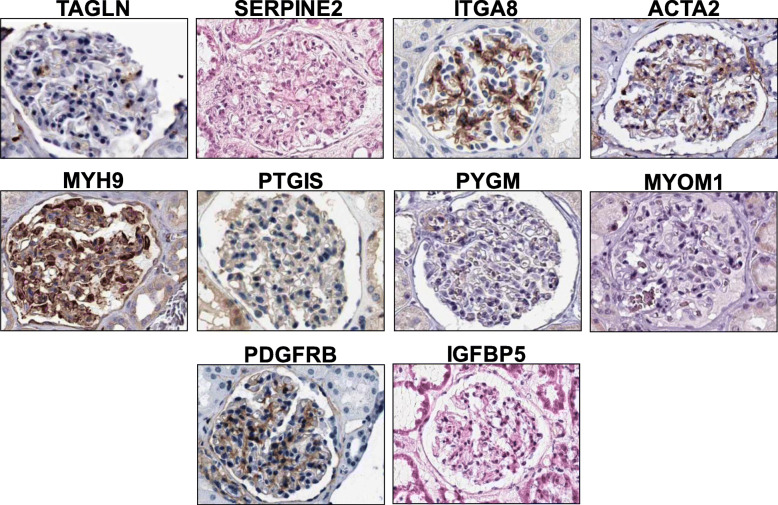
Immunohistochemistry showing glomerular expression patterns of the selected monotonic ascending pattern target genes with DE ≤ 4. These genes were expressed in human mesangial cells in glomeruli. These images were collected from the Human Protein Atlas (www.proteinatlas.org) after cropping the glomeruli from the original full images

Ten ascending pattern key genes with DE ≤ 4 were selected for further analysis (Fig. 3a). These selected key genes are related to mesangial cells or smooth muscle cells (*TAGLN, SERPINE2*, *PYGM* and *IGFBP5*), contraction (*ACTA2*, *MYH9*, *MYOM1*, *PDGFRB* and *PTGIS*) or phagocytosis (*ITGA8*). The expression of these selected key genes in mesangial cells is also being reported in other research. In order to confirm expression of these key genes in human mesangial cells, we used the human protein atlas (http://www.proteinatlas.org/) (Fig. 4).

By referring to the TRR databases, the upstream regulators or TFs for these key genes were identified and these TFs were located in between L7 and L9 in TO-GCN (Fig. 3b). TFs *TEAD3* and *NFE2* were each regulated 4 key genes. TF *TEAD3* regulated *ACTA2*, *MYOM1, PTGIS* and *TAGLN*, while TF *NFE2* regulated *ACTA2, MYOM1, PYGM* and *SERPINE2*. Both TFs *SRF* and *ACTA2* regulated 3 key genes each. TF *SRF* regulated *TAGLN*, *PYGM* and *ACTA2,* while TF *ETV6* regulated *MYOM1, IGFBP5* and *PTGIS*. Some of the TF-key gene relationships mentioned above were also found in ENCODE TF-Targets Dataset (Additional file 5B).

### Functional enrichment analysis in each TO-GCN level

A total of 69 pathways were enriched (FDR < 0.05) in these 9 levels gene co-expression network. Each level enriched a range of 4 to 31 pathways. Full list of enriched pathways in each level is shown in Additional file 6. Since a gene may co-express with TFs at multiple levels, two neighbouring gene sets might have some overlapping genes.

**Fig. 5:**
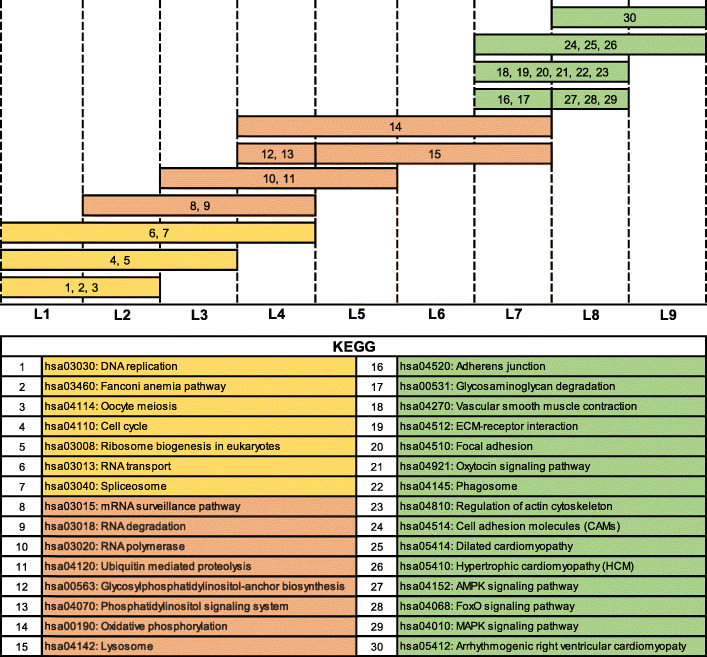
Selected analysis of functional pathways for each level. Three developmental-stage transitions can be observed. Stage 1: differentiation preparation stage (orange); pathways related to cell proliferation have been enriched. Stage 2: differentiation initiation stage (red); pathways related to regulating or driving differentiation have been enriched. Stage 3: maturation stage (green); pathways related to mesangial cell function and characteristics have been enriched

By identifying the enriched pathways among the co-expressed genes at each level of TO-GCN, three developmental-stage transitions can be observed (differentiation preparation, differentiation initiation and maturation) (Fig. 5). In between L1 and L4, pathways related to cell proliferation in the differentiation preparation stage, like cell cycle (L1 to L3) and DNA replication (L1 to L2) were enriched. Pathways related to cell differentiation preparation pathways, such as ribosome biogenesis in eukaryotes (L1 to L3), were also enriched.

Pathways related to initiation or driving differentiation were enriched at L2 to L7. The enriched pathways include mRNA surveillance pathway (L2 to L4), RNA degradation (L2 to L4), RNA polymerase (L3 to L5) and ubiquitin mediated proteolysis (L3 to L5). From L7 and upward, several mesangial cell associated pathways were enriched indicating a shift to maturation stages of the differentiated cells. These enriched pathways include vascular smooth muscle contraction (L7 to L8), regulation of actin cytoskeleton (L7 to L8), phagosome (L7 to L8) and cell adhesion molecules (L7 to L9).

### Co-cultured MSC has contraction capability

**Fig. 6:**
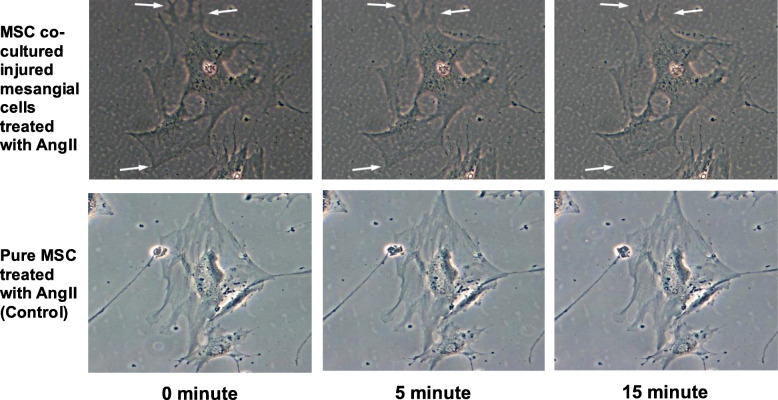
For purposes of validating one of the KEGG functional pathway: Vascular smooth muscle contraction (hsa04270), has been enriched in differentiated cells by bioinformatics analysis. MSC co-cultured with injured mesangial cells were treated with AngII; cell contraction was observed as indicated by white arrows. Pure MSC (Control) did not show any contraction after treated with AngII

One characteristic of mesangial cells is that they contract in response to vasoactive peptides, for example AngII, under in vitro conditions. In this study, one of the KEGG functional pathway: Vascular smooth muscle contraction (hsa04270), was enriched in differentiated cells by bioinformatics analysis and was performed the with wet lab validation. Co-cultured MSC or differentiated cells were contracted once treated with AngII with the obvious contraction observable at the edge of the cell (Fig. 6), while the MSC population only (Control) did not show any contraction after being treated with AngII. The contraction video of MSC differentiated mesangial cells can be viewed in Additional file 7.

**Additional file 7.** Differentiated mesangial cells contraction video.

### TF-TF-key genes relationship with vascular smooth muscle contraction related genes

**Fig. 7:**
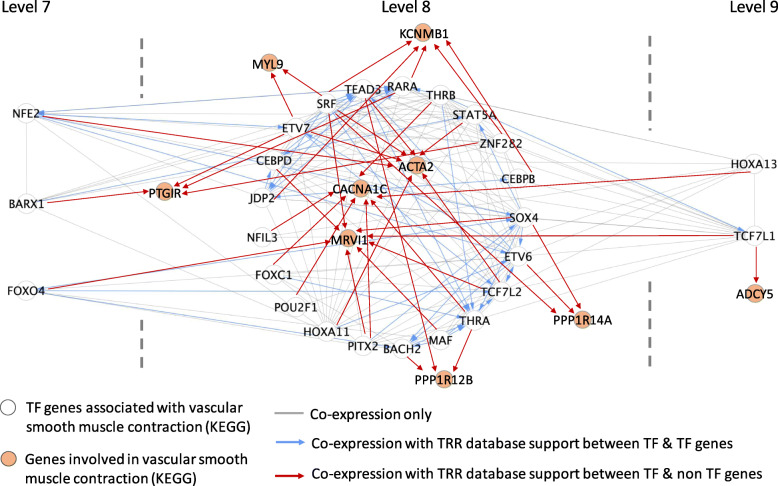
Co-expression network of selected vascular smooth muscle contraction related biomarkers with their upstream regulators/TFs. Co-expression with TRR database support between TF-TF genes were linked with blue lines. While co-expression with TRR database support between TF-non TF genes were linked with red lines

From hsa04270: Vascular smooth muscle contraction (KEGG) gene list, 9 key genes with DE ≤ 4 were selected: *PTGIR, MYL9, KCNMB1, ACTA2, CACNA1C, MRV11, PPP1R12B, PPP1R14A* and *ADCY5* (Fig. 7). These 9 key genes were found regulated by 26 TFs after reference to TRR databases. From these 9 key genes and 26 TFs, a network was constructed based on co-expression. Some of the TF-key gene relationships mentioned above were also found in ENCODE TF-Targets Dataset (Additional file 5C).

## Discussion

### Construction and robustness of TO-GCN

As there are various gene expression patterns in the time-series transcriptome data, we sought to construct relationships between TFs by examining their pattern similarity (PCC) in TO-GCN. This meant that the total number of levels represents the dynamic range of different expression patterns in TO-GCN. In this study, the number of levels in TO-GCN was dependent on the PCC cut-off setting value. The more stringent correlation coefficient or a higher PCC value that is set, the more levels in TO-GCN will be constructed. In contrast, if PCC cut-off is set at lower value, more TFs will be grouped in a single level and will therefore yield TO-GCN with lesser levels. In this study, PCC was set at ≥0.91 (*p*-value < 0.05) and 9 levels of TO-GCN were constructed.

In order to demonstrate the robustness of TO-GCN, we tested the level order stability by using 7 different TF genes with DE ≤ 4 and was co-expressed with *FOSL1* (PCC > 0.99) as new initial nodes to construct the corresponding TO-GCNs. The analysis showed that new ordered TO-GCNs are very similar to the original TO-GCN that was constructed with *FOSL1* (Additional file 8).

### Synergistic work between MFSelector and TO-GCN

MFSelector and TO-GCN were the two methods of data analysis used in this study. These methods worked together synergistically to provide a deeper understanding of MSC differentiation into mesangial cells. MFSelector determined the degree of monotonicity for all genes during the differentiation process and it provided an estimation of the expression behaviour of the gene during differentiation. TO-GCN used co-expression relationship to connect TF genes as pairs, in which they have similar expression patterns (i.e. significantly high PCC) over time. It inferred expression time orders for all TF genes in the network with the starting TF in the strongest descending pattern identified by MFSelector. By applying this method to time-series experiments, TO-GCN provided the time order information of gene regulations in developmental processes. The data obtained from both methods was further used to identify the TF-key genes at specific time points to the TO-GCN at different levels. This helped to elucidate the network interaction between TF-TF and TF-key genes at each level of TO-GCN.

In this study, the *FOSL1* gene, expressed in the strongest monotonic descending pattern, was used as initial node. As the network was constructed based on co-expression, TFs in the same or next levels of *FOSL1* in TO-GCN would be also in a descending patterns. This was consistent with the genes in descending pattern identified by MFSelector. Lower DE values (stronger monotonic pattern) of descending pattern TFs appeared in early levels from L1 to L2 (green nodes in Fig. 1). The ascending TFs with higher monotonicity (lower DE value) appeared later at the levels from L7 to L9 (purple nodes in Fig. 1). Genes with a weak monotonic pattern (either descending or ascending) were located in between the descending and ascending high monotonicity pattern genes.

### The key genes and MSC biomarkers were down-regulated during the differentiation process

MSC biomarkers such as *ANPEP* and *LIF* were down-regulated during the differentiation process. *ANPEP*, also called CD13, is well known as an MSC marker. On the other hand, *LIF*, another well-established MSC marker, has been reported to affect cell growth by inhibiting differentiation but maintaining the stemness of the stem cell. When *LIF* expression levels drop, the cells will start the process of differentiation [14]. Meanwhile, depletion of *AURKA*, known for stem cell renewal, resulted in compromised self-renewal and consequent differentiation [15].

In this study, many genes related to cell cycle regulation (*CDK1*, *CCNB1* and *GNL3*) and DNA replication (*CDC6*) were down-regulated. *CDK1* is a key regulator of mitosis. High expression levels of *CDK1* are associated with the pluripotency stage of embryonic stem cells (ESC). Decreased *CDK1* activity to a level without perturbing the cell cycle is sufficient to induce differentiation [16]. Meanwhile *CCNB1* gene expression increases during G2/M phase and decreases during terminal differentiation [17]. *GNL3*, also known as nucleostemin, regulates the cell cycle and affects cell differentiation; the amount of *GNL3* decreases as differentiation progresses. *GNL3* is also a biomarker for many stem cells and cancer cells [18]. *CDC6* is an essential regulator of DNA replication in eukaryotic cells. Down regulation of *CDC6* will lead to a drop of DNA replication before differentiation can take place [19, 20]. Even though these genes regulate the cell cycle or DNA replication, all findings show that when these genes are down-regulated in stem cells, differentiation will start.

By referring to the TRR databases, 3 TFs (*MEOX2*, *SOX9* and *HMGA1*) regulated MSC markers such as *LIF* and *ANPEP*. These TFs are known as regulators of the stem cell state through transcriptional networks that induce pluripotency. Theodorou et al. reported that neuronal differentiation in ESC was inhibited when *MEOX2* is overexpressed [21]. Shah et al. did a study showing that when ESC differentiation was induced, there was a decreased expression of *HMGA1* which was also observed in other pluripotency factors. Conversely, forced expression of *HMGA1* blocked the differentiation of ESC [22]. Meanwhile for *SOX9*, upon the differentiation of MSC into hepatocytes, *SOX9* expression was down-regulated [23].

### Biomarkers contribute to mesangial cell characteristics and functions

Ten mesangial cell key genes with DE ≤ 4 were selected for further analysis. The majority of these key genes are reported as biomarkers for mesangial cells or related to the functions of mesangial cells. *TAGLN*, or SM22-alpha, is expressed in smooth muscle cells. It is known as one of the earliest commitment biomarkers of differentiated smooth muscle cells and has been suggested to regulate their contractile functions [24]. This gene has a role in generating committed progenitor cells from undifferentiated hMSC by regulating cytoskeleton organization. *TAGLN* in the kidney is up-regulated in repopulating mesangial cells in vivo. Meanwhile *SERPINE2* and *IGFBP5* are reported to be expressed in mesangial cells [25, 26] and *MYOM1* is known to be expressed in smooth muscle cells [27].

*ACTA2* and *MYH9* play an important role in regulating both smooth muscle and non-muscle cell contractile activity [25, 28]. Another contraction related gene is *PTGIS*, also known as prostacyclin synthase. *PTGIS* is the final committed enzyme in the metabolic pathway leading to prostaglandin I2 (PGI2) production and PGI2 is needed to mediate mechanism of vascular contraction [29]. *PDGFRB* is needed for stimulation of contraction and chemotaxis [30]. *PYGM* encodes a muscle enzyme that is involved in glycogenolysis.

Mesangial cells are phagocytic cells and expression of *ITGA8* in mesangial cells facilitates phagocytosis. About 15% of the total mesangial cell population in the glomerulus is capable of exhibiting immunological function such as phagocytosis [31].

By referring to the TRR databases, TFs *SRF* and *TEAD3* were found to regulate 3 and 4 key genes respectively. This shows that TF *SRF* and *TEAD3* play an important role in mesangial cells. *SRF* is a ubiquitous expressed TF that drives smooth muscle cell-specific gene expression and is necessary for contractile and cytoskeletal functions [32, 33]. *TEAD3* has been reported to abolish myocardin function and is consistently expressed in smooth muscle cells [34].

### Pathway enrichment analysis on each TO-GCN level

Proliferation and differentiation processes are two distinct and mutually exclusive processes during development. To initiate stem cell differentiation, certain cell proliferation related genes or pathways have to be down-regulated. Estefanía et al. has reported that terminal differentiation is the process by which dividing cells stop proliferating in order to start new specific functions, which means that DNA replication fades as cells advance in their commitment to terminal differentiation [35]. Therefore, these early levels can be classified as differentiation preparation.

From L2 to L7, pathways involved in regulating or triggering differentiation were enriched. Lou et al. showed that RNA degradation drives stem cell differentiation [36]. They discovered that the steady-state level of RNAs is dictated by their decay rate and this specific RNA decay such as Nonsense-mediated mRNA decay (NMD) have a role in promoting differentiation mechanisms [37, 38]. NMD is a surveillance pathway and its main function is as a quality control pathway to reduce errors in gene expression by eliminating mRNA transcripts that contain premature stop codons [39]. During the differentiation, NMD elicits the decay of specific subsets of mRNAs and promotes the decay of mRNAs encoding pluripotency factors [36].

From L7 to L9, pathways involved in mesangial cell maturation were enriched. Some of the enriched pathways are related to characteristics and functions of mesangial cells such as contraction and phagocytosis ability. Therefore it is not surprising to find that the oxytocin signaling pathway, phagosome and certain cardiac related pathways were enriched.

The strongest evidence that the co-cultured MSC have differentiated into mesangial cells is by confirming the pathway enrichment of vascular smooth muscle contraction [40]. As mesangial cells are modified smooth muscle cells, we have further conducted a wet lab contraction functional validation. Results showed that the differentiated cells can contract and have proven that the cells have fully matured in their differentiation in which the cells now possess mesangial cell functions. On the other hand, pure MSC failed to exhibit contraction ability.

### Construction of vascular smooth muscle contraction-specific gene network

In this study we have shown that differentiated mesangial cells have contraction ability. With this specific biological function, a co-expression gene network related to vascular smooth muscle contraction was constructed. This network has illustrated the relationship between key genes and its upstream regulators or TFs. To our knowledge, such function-specific TF-TF-vascular smooth muscle contraction-related key gene network has not been reported before. This indicates that a mathematically calculated co-expression network can provide us with a first step or hints for further wet lab validation before full biological TF-TF-key gene relationship is fully uncovered. Other biological functions such as phagocytosis of mesangial cells can also be explored and constructed using the methods presented herein.

## Conclusions

In our previous studies, we developed two approaches to respectively identify monotonic genes and build a TF co-expression network in order of time. We demonstrated that genes with ascending or descending monotonic expression patterns in chronological stem cell data are involved in differentiation of stem cells into variant cell lineages or the proliferation or self-renewal activity of stem cells [12]. We also verified the regulatory relationship of TF-TF network in maize and rice leaf time-series transcriptomic data [13]. In this study, we developed a systematic analysis pipeline to integrate these two approaches to investigate the transcriptomic profiling of MSC differentiation into mesangial cells. Here we demonstrated that these two approaches are able to work synergistically to provide better understanding of MSC differentiation into mesangial cells by identification of key genes or biomarkers, TFs and pathways involved in differentiation of MSC-mesangial cells. In addition, the proposed method can be used to further classify the differentiation process into three stages: differentiation preparation, differentiation initiation and maturation, and to identify novel TF-TF-key genes regulatory relationships in a specific biological process/pathway (here vascular smooth muscle contraction). This study provides results which support further wet lab validation leading then to full discovery of biological TF-TF-key gene relationships.

These two approaches are not only limited to the investigation of the gene expression changes in stem cells differentiation process but can also be applied to other time-series data analysis. MFSelector has been used to evaluate gene expression changes across the pathologic stages of non-small cell lung cancer [41]. With the same application in this study, MFSelector can be used to identify or discover cancer markers for the specific cancer, and TO-GCN can been used to predict regulatory networks; revealing the dynamics of the biological process.

Through our study, we have increased fundamental understanding of the gene transcripts as stem cells differentiate into terminal cells, in this case from MSC to mesangial cells. Our findings can offer new opportunities to develop cellular therapies for pathologic conditions such as glomerulonephritis or diabetic nephropathy that lead to renal failure, and treatments for other diseases.

## Methods

### Cell cultures and RNA extraction

The induction of MSC-mesangial cell differentiation by co-culturing MSC with oxidant-injured mesangial cells was described in a previous report [42]. Briefly, MSC were co-cultured with hydrogen peroxide-injured commercially available human mesangial cells (Clonetics™ Normal Human Mesangial Cells, Lonza, Cat# CC-2559) (*n* = 3) in trans-well dishes separated by a membrane for 7 days. In this study, three biological replicates (*n* = 3) were co-cultured in two batches. Human bone marrow-derived MSC samples used in this study were sponsored by Cytopeutics Sdn. Bhd. with signed informed consent from all donors.

To confirm that MSC differentiated into mesangial cells, day 7 co-cultured MSC were harvested and tested for immunophenotyping and functional assay. The findings showed that co-cultured MSC differentiated into mesangial cells by exhibiting the immunophenotype, the morphology and functional assay profile of mesangial cells [42].

**Fig. 8:**
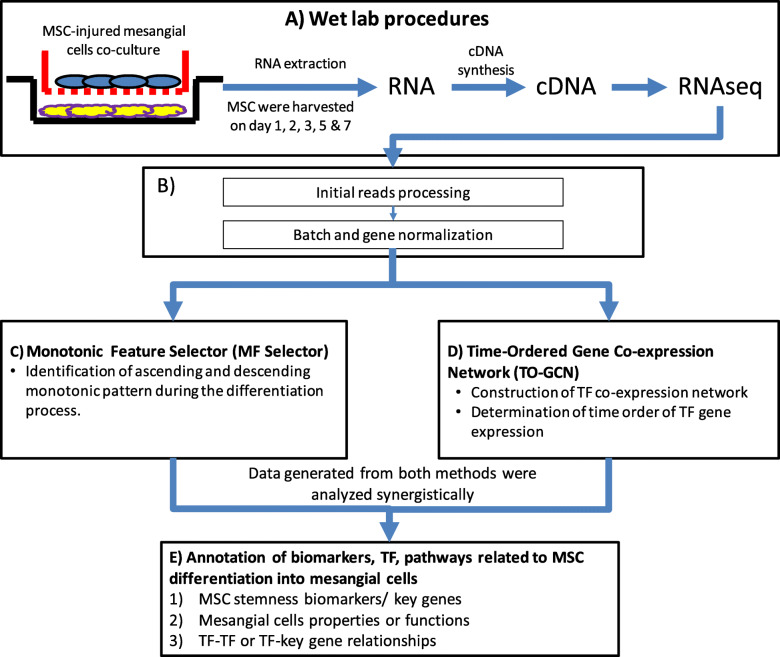
Experimental design of the study. **a** MSC were co-cultured with injured mesangial cells. On indicated days, co-cultured MSC were harvested and RNA was extracted. cDNA was synthesised and subsequently underwent RNA-seq. **b** After RNA-seq, raw data was processed by initial read processing and normalizations before the data were further analyzed simultaneously using two methods. **c** Key genes related to MSC or mesangial cells with ascending or descending monotonic patterns over the differentiation process were identified with MFSelector. **d** Co-expression patterns between TF and construction TF co-expression network were determined by TO-GCN. Once the data from both methods was generated, both sets of data were analysed synergistically (**e**). The data obtained from both methods were used to correlate the key genes at specific time points to the TO-GCN at different levels. This helps to elucidate the network interaction between TF-TF and TF-key genes at each level of TO-GCN. Additionally, this builds our understanding of the overall gene expression during the MSC differentiation into mesangial cells

In this RNA-seq study on the indicated days (day 1, 2, 3, 5 and 7), co-cultured MSC were harvested and RNA was extracted with a Quick-RNA™ MiniPrep kit (Zymo Research), according to the manufacturer’s instructions. UV spectrophotometry confirmed that the RNA preparations were free of proteins and phenol. The quality and integrity of the RNA isolated were assessed on the Agilent 2100 Bioanalyzer (Agilent Technologies). Only RNA samples free of proteins and phenol that featured an RNA Integrity Number (RIN) > 8.0 were used (Fig. 8).

### Complementary DNA generation, RNA sequencing, mapping and initial read processing

RNA-seq data for MSC during the differentiation process at different time points was generated using next generation sequencing (Fig. 8a). Poly-A mRNA selection and synthesis of a complementary DNA (cDNA) library were carried out following the Illumina TruSeq protocol. Single-end 75 bp length, 100 M reads/lane sequencing was performed on the Hiseq 2000 genome analyzer platform (Illumina). The sequencing results from poly A positive RNA were analyzed by FastQC program (www.bioinformatics.babraham.ac.uk) to monitor the read quality (Q30 > 85%). The processed reads were mapped to Genome assembly version GRCh38 (GENCODE release 33) with STAR (version 2.7.3a) (https://code.google.com/archive/p/rna-star/) [43] using the 2-pass workflow and parameters that recommended by ENCODE. The gene level read counts were evaluated by RSEM (version 1.3.3) with the same transcript information from GENCODE.

Then only genes without any zero counts across samples were kept for the following processes performed with the statistical language R. First, the raw read counts of samples of Days 1, 2, 3, 5, 7 were normalized with the TMM algorithm provided in the R package edgeR [44]. Next, the batch effects were removed with the ComBat function included in the R sva package. The non-parametric empirical Bayes framework was used with the known batch and group covariates, and the given reference-batch (batch 2).

### Monotonic feature selector and data annotation

In order to fully understand the differentiation process, MFSelector was used to identify genes with ascending or descending monotonic expression patterns over time (Fig. 8c). Details of the system was described in a previous report [12]. Briefly, genes with ascending or descending monotonic expression patterns were identified with MFSelector based on a concept of discriminating error (DE). In this system, various time stages in stage order (i.e. Stage One vs. other stages, Stages One and Two vs. remaining stages and so on) were evaluated and DE for each gene was computed with MFSelector. The value of DE represents the level of monotonic pattern for the genes. The lower the value of DE, the greater the strength of the monotonic pattern of the gene. In this study, both the permutation times for the computations of p, q-values and the sample variance for discriminating error were set to 100. DE of ≤4 was set to obtain certain number of genes with statistically significant p−/q-values for further analysis (i.e., each gene with a good monotonicity to show fewer outliers appearing in a specific time point among the 5 time points) this also help to acquire other closely related enrichment pathways and functions. The MFSelector R function and data sets can be downloaded from http://microarray.ym.edu.tw/tools/MFSelector

### Construction of the time-ordered gene co-expression network (TO-GCN)

The TO-GCN was constructed by the modified version of the method proposed by Chang et al. (https://github.com/petitmingchang/TO-GCN) [13]. The TF gene annotation was downloaded from animalTFDB 3.0 [45]. The method consists of three steps: (1) determining co-expression cutoffs, (2) constructing GCNs, and (3) determining the time order of TF gene expression. (Fig. 8d).

In this study, the expression profile for each gene based on the transcriptomes consists of five time points. The Pearson correlation coefficient (PCC) values for all TF-TF pairs were calculated and the cutoff of positive co-expression PCC ≥ 0.91 (*p*-value < 0.05) was determined. A gene co-expression network (GCN) was constructed from 1191 TF genes. *FOSL1* with the strongest monotonic descending pattern (DE = 0) in this study was selected as the initial node to generate all time-ordered levels of nodes in the GCN by the breadth-first search algorithm.

### Co-expressed gene sets and enrichment pathways and functions in each TO-GCN level

For the TF at each level of a TO-GCN, a corresponding set of co-expressed non-TF genes with the same co-expression relationship (PCC ≥ 0.91, *p*-value < 0.05) were added to that particular level in TO-GCN. Since non-TF genes may be co-expressed with TFs in multiple levels, two neighbouring gene sets will have some overlapping genes.

For each set of genes corresponding to a level in a TO-GCN, the pathway and functional enrichment analysis was conducted with the background set of all expressed genes in this study. DAVID (Database for Annotation, Visualization and Integrated Discovery, https://david.ncifcrf.gov/) by cross-referencing to the KEGG (Kyoto Encyclopedia of Genes and Genomes, https://www.genome.jp/kegg/) database was used in these functional annotations. Enriched pathways with FDR (Benjamini-Hochberg method) < 0.05 were used for further annotation.

### Inferences of gene regulation network

In each co-expression, edges (TF-TF) were built. A further check carried out into whether any built edges were reported in two TRR databases: Marbach (http://regulatorycircuits.org/) [46] and TRRUSTv2 (www.grnpedia.org/trrust) [47]. If yes, then those particular edges were labelled with different colours to indicate such edges not just based on co-expression relationship but with TRR database support as well. To improve this further, all edges that were positive for either Marbach or TRRUSTv2 were checked on the ENCODE TF-Targets Dataset (https://amp.pharm.mssm.edu/Harmonizome/dataset/ENCODE+Transcription+Factor+Targets). This dataset provides the TF binding site profiles that measured by ChIP-seq [48].

### Identification of key genes or biomarkers changes during differentiation

Several key genes with monotonic ascending or descending patterns with DE ≤ 4 were selected and further analysed by examining the functions of these genes in relation to MSC differentiation into mesangial cells. The upstream regulator or TF to regulate these key genes was checked by the TRR databases mentioned above and the TF-key gene or biomarker relationship was annotated into TO-GCN.

### Differentiated cell contraction study

For purposes of validating one of the KEGG functional pathway: Vascular smooth muscle contraction (hsa04270) has been enriched in differentiated cells by bioinformatics analysis, on day 7 of co-culture, a portion of the harvested co-cultured MSC were plated at a density of 1 × 10^4^ per well in new six-well plates with new fresh culture media. After 24 h, the culture medium was replaced with Hanks’ balanced salt solution. The cells were incubated and stimulated with 10 nmol/L of Angiotensin II (AngII) (Sigma), one type of vasoactive peptides. Images of cells were recorded for 15 min and changes in cell morphology were observed accordingly. Pure MSC population (without being co-cultured with mesangial cells) was treated with AngII and served as the control.

### TF-TF-key gene relationship for vascular smooth muscle contraction related genes

Contraction ability is one of the mesangial cell’s properties. A gene network was constructed specifically for vascular smooth muscle contraction related genes. Genes in hsa04270: Vascular smooth muscle contraction (KEGG) was used as initial gene lists. From the gene list, only monotonic ascending pattern key genes with DE ≤ 4 were selected and used for further analysis. TFs to regulate these key genes were traced by TRR databases mentioned above. A co-expression network was constructed from these selected muscle contraction related key genes and their TFs.

## Supplementary information

**Additional file 1.** MDS plot and heatmap.

**Additional file 2.** TF in TO-GCN.

**Additional file 3.** Monotonic descending pattern genes with DE ≤ 4.

**Additional file 4.** Monotonic ascending pattern genes with DE ≤ 4.

**Additional file 5.** Inferences of selected genes to Marbach, TRRUSTv2 and ENCODE TF-Targets dataset.

**Additional file 6.** List of enriched pathways in each level.

**Additional file 8.** TO-GCN robustness analysis.

## Data Availability

Raw transcriptomic data generated in this study have been deposited into National Center for Biotechnology Information Sequence Read Archive (SRA), accession number SRP233161. (https://www.ncbi.nlm.nih.gov/sra/SRP233161). Monotonic Feature Selector R function program can be downloaded from http://microarray.ym.edu.tw/tools/MFSelector. Time-Ordered Gene Co-expression Network analysis program can be downloaded from https://github.com/petitmingchang/TO-GCN. Two transcriptional regulatory relationships databases used in this study were Marbach from Regulatory Circuits (http://regulatorycircuits.org/) and TRRUST version 2 (Transcriptional Regulatory Relationships Unravelled by Sentence-based Text Mining; www.grnpedia.org/trrust). ENCODE (ENCyclopedia Of DNA Elements) Transcription Factor-Targets Dataset was obtained from Harmonizome website (https://amp.pharm.mssm.edu/Harmonizome/dataset/ENCODE+Transcription+Factor+Targets).

## Abbreviations

AngII: Angiotensin II
cDNA: Complementary DNA
DE: Discriminating error
GCN: Gene co-expression network
KEGG: Kyoto Encyclopedia of Genes and Genomes
L: Level
MFSelector: Monotonic Feature Selector
MSC: Mesenchymal stem cells
NMD: Nonsense-mediated mRNA decay
PCC: Pearson correlation coefficient
PGI2: Prostaglandin I2
RNA: Ribonucleic acid
TF: Transcription factor
TO-GCN: Time-Ordered Gene Co-expression Network
TRR: Transcriptional regulatory relationships
