# Supplementary material for: Decoding the differentiation of mesenchymal stem cells into mesangial cells at the transcriptomic level
Source: BMC Genomics. 2020 Jul 7;21:467. doi: 10.1186/s12864-020-06868-5 (PMC7339572; doi:10.1186/s12864-020-06868-5)
Supplement: Supplementary file 2 — Additional file 1. MDS plot and heatmap. [file 12864_2020_6868_MOESM1_ESM.pdf]

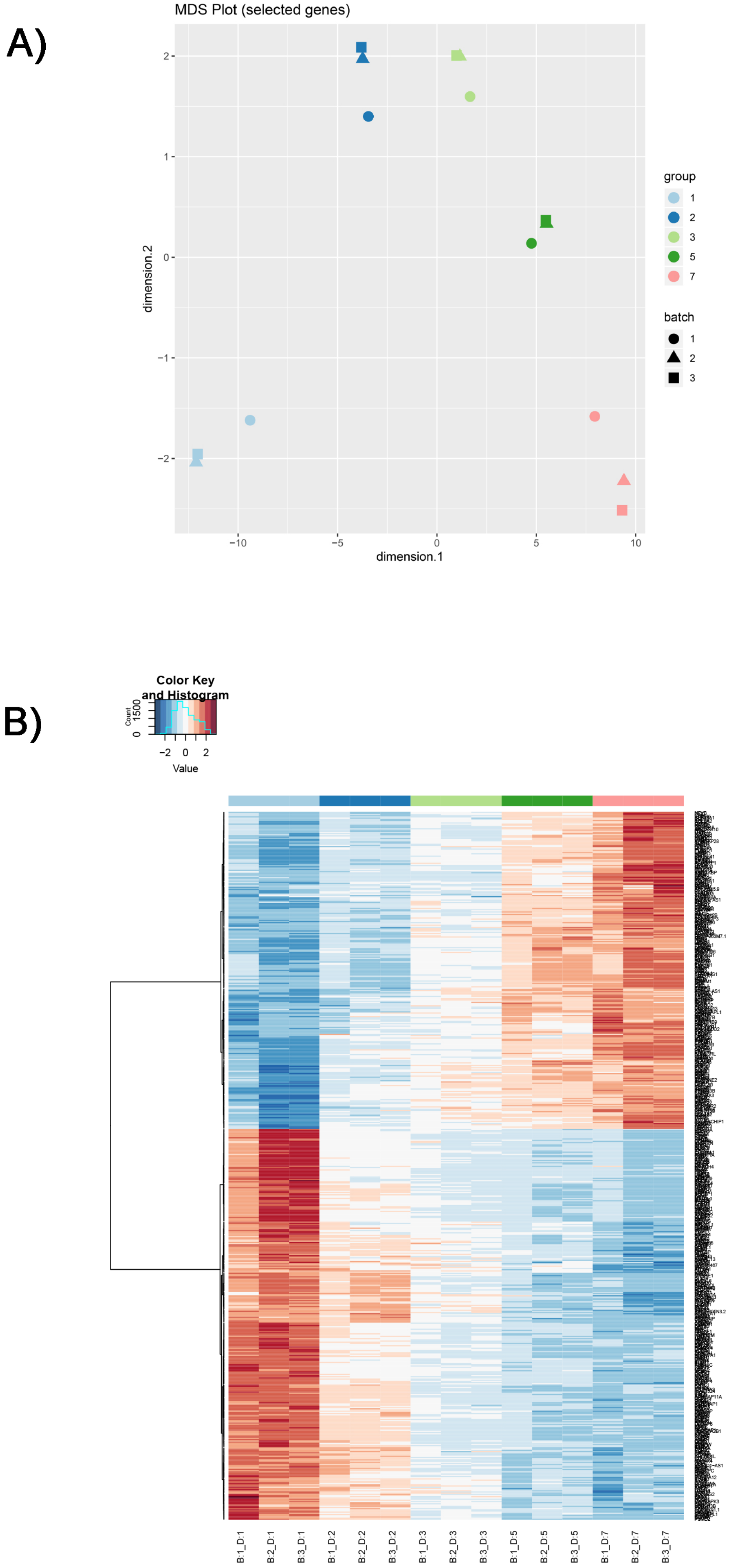

Genes with DE≤2 were selected to generate the multidimensional scaling (MDS) plot (A) and heatmap (B) to show the effect of the batch adjustment and monotonic gene expression patterns across the samples at different days respectively.
