# Supplementary material for: Decoding the differentiation of mesenchymal stem cells into mesangial cells at the transcriptomic level
Source: BMC Genomics. 2020 Jul 7;21:467. doi: 10.1186/s12864-020-06868-5 (PMC7339572; doi:10.1186/s12864-020-06868-5)
Supplement: Supplementary file 3 — Additional file 2. TF in TO-GCN. [file 12864_2020_6868_MOESM2_ESM.pdf]

TF in TO-GCN

	Ensembl Gene ID	Gene Names	TF family	TF level	Descending (DE)	Ascending (DE)
1	ENSG00000175592	*FOSL1*	TF_bZIP	1	0	12
2	ENSG00000111206	*FOXM1*	Fork_head	1	1	12
3	ENSG00000124191	*TOX2*	HMG	1	1	12
4	ENSG00000137309	*HMGA1*	HMGA	1	1	12
5	ENSG00000029993	*HMGB3*	HMG	1	2	12
6	ENSG00000081059	*TCF7*	HMG	1	2	12
7	ENSG00000101057	*MYBL2*	MYB	1	2	12
8	ENSG00000101412	*E2F1*	E2F	1	2	12
9	ENSG00000137310	*TCF19*	Others	1	2	12
10	ENSG00000165244	*ZNF367*	zf-C2H2	1	2	12
11	ENSG00000165891	*E2F7*	E2F	1	2	12
12	ENSG00000176692	*FOXC2*	Fork_head	1	2	12
13	ENSG00000196460	*RFX8*	RFX	1	2	12
14	ENSG00000244405	*ETV5*	ETS	1	2	12
15	ENSG00000103326	*CAPN15*	Others	1	3	12
16	ENSG00000108511	*HOXB6*	Homeobox	1	3	12
17	ENSG00000115163	*CENPA*	Others	1	3	12
18	ENSG00000125347	*IRF1*	IRF	1	3	12
19	ENSG00000149948	*HMGA2*	HMGA	1	3	12
20	ENSG00000172819	*RARG*	THR-like	1	3	12
21	ENSG00000173153	*ESRRA*	ESR-like	1	3	12
22	ENSG00000006468	*ETV1*	ETS	1	3	12
23	ENSG00000109685	*NSD2*	HMG	1	3	12
24	ENSG00000115844	*DLX2*	Homeobox	1	3	12
25	ENSG00000149480	*MTA2*	zf-GATA	1	3	12
26	ENSG00000152784	*PRDM8*	zf-C2H2	1	3	12
27	ENSG00000007968	*E2F2*	E2F	1	4	12
28	ENSG00000115816	*CEBPZ*	Others	1	4	12
29	ENSG00000135111	*TBX3*	T-box	1	4	12
30	ENSG00000144355	*DLX1*	Homeobox	1	4	12
31	ENSG00000167491	*GATAD2A*	zf-GATA	1	4	12
32	ENSG00000196793	*ZNF239*	zf-C2H2	1	4	12
33	ENSG00000101096	*NFATC2*	RHD	1	4	12
34	ENSG00000108064	*TFAM*	HMG	1	4	12
35	ENSG00000143867	*OSR1*	zf-C2H2	1	4	12
36	ENSG00000164104	*HMGB2*	HMG	1	4	12
37	ENSG00000197961	*ZNF121*	zf-C2H2	1	4	12
38	ENSG00000198554	*WDHD1*	HMG	1	4	12
39	ENSG00000174738	*NR1D2*	THR-like	1	4	11
40	ENSG00000105821	*DNAJC2*	MYB	1	5	12
41	ENSG00000116560	*SFPQ*	Others	1	5	12
42	ENSG00000117010	*ZNF684*	zf-C2H2	1	5	12
43	ENSG00000129173	*E2F8*	E2F	1	5	12
44	ENSG00000175832	*ETV4*	ETS	1	5	12
45	ENSG00000078399	*HOXA9*	Homeobox	1	5	12
46	ENSG00000121068	*TBX2*	T-box	1	5	12
47	ENSG00000169689	*CENPX*	Others	1	5	12
48	ENSG00000179388	*EGR3*	zf-C2H2	1	5	12
49	ENSG00000181472	*ZBTB2*	ZBTB	1	5	12
50	ENSG00000189403	*HMGB1*	HMG	1	5	12
51	ENSG00000106511	*MEOX2*	Homeobox	1	5	11
52	ENSG00000149136	*SSRP1*	HMG	1	6	12
53	ENSG00000105750	*ZNF85*	zf-C2H2	1	6	12
54	ENSG00000162599	*NFIA*	CTF/NFI	1	6	12
55	ENSG00000095794	*CREM*	TF_bZIP	1	7	12
56	ENSG00000059378	*PARP12*	Others	1	7	12
57	ENSG00000134532	*SOX5*	HMG	1	7	12
58	ENSG00000138795	*LEF1*	HMG	1	7	12
59	ENSG00000197124	*ZNF682*	zf-C2H2	1	7	11
60	ENSG00000153234	*NR4A2*	NGFIB-like	1	8	12
61	ENSG00000164379	*FOXQ1*	Fork_head	1	8	12
62	ENSG00000183647	*ZNF530*	zf-C2H2	1	8	12
63	ENSG00000103343	*ZNF174*	zf-C2H2	1	8	11
64	ENSG00000120738	*EGR1*	zf-C2H2	1	8	9
65	ENSG00000125968	*ID1*	bHLH	2	2	12
66	ENSG00000008083	*JARID2*	ARID	2	2	11
67	ENSG00000177426	*TGIF1*	Homeobox	2	3	12
68	ENSG00000125398	*SOX9*	HMG	2	3	10
69	ENSG00000108788	*MLX*	bHLH	2	4	12
70	ENSG00000088247	*KHSRP*	Others	2	4	12
71	ENSG00000182903	*ZNF721*	zf-C2H2	2	4	11
72	ENSG00000029153	*ARNTL2*	bHLH	2	4	10
73	ENSG00000126368	*NR1D1*	THR-like	2	4	10
74	ENSG00000127337	*YEATS4*	Others	2	5	12
75	ENSG00000129028	*THAP10*	THAP	2	5	12
76	ENSG00000198028	*ZNF560*	zf-C2H2	2	5	12
77	ENSG00000213347	*MXD3*	bHLH	2	5	12
78	ENSG00000111145	*ELK3*	ETS	2	5	12
79	ENSG00000113387	*SUB1*	PC4	2	5	12
80	ENSG00000114315	*HES1*	bHLH	2	5	12
81	ENSG00000170684	*ZNF296*	zf-C2H2	2	5	12
82	ENSG00000197714	*ZNF460*	zf-C2H2	2	5	12
83	ENSG00000198176	*TFDP1*	E2F	2	5	12
84	ENSG00000197472	*ZNF695*	zf-C2H2	2	5	11
85	ENSG00000028277	*POU2F2*	Pou	2	5	11
86	ENSG00000135547	*HEY2*	bHLH	2	5	11
87	ENSG00000185960	*SHOX*	Homeobox	2	5	11
88	ENSG00000168874	*ATOH8*	bHLH	2	5	11
89	ENSG00000001167	*NFYA*	NF-YA	2	5	10
90	ENSG00000116132	*PRRX1*	Homeobox	2	5	10
91	ENSG00000182158	*CREB3L2*	TF_bZIP	2	5	10

92	ENSG00000138375	*SMARCAL1*	Others	2	5	9
93	ENSG00000176678	*FOXL1*	Fork_head	2	5	9
94	ENSG00000066136	*NFYC*	NF-YC	2	6	12
95	ENSG00000171466	*ZNF562*	zf-C2H2	2	6	12
96	ENSG00000173917	*HOXB2*	Homeobox	2	6	12
97	ENSG00000175279	*CENPS*	Others	2	6	12
98	ENSG00000197063	*MAFG*	TF_bZIP	2	6	12
99	ENSG00000102901	*CENPT*	Others	2	6	12
100	ENSG00000122691	*TWIST1*	bHLH	2	6	12
101	ENSG00000132010	*ZNF20*	zf-C2H2	2	6	12
102	ENSG00000167085	*PHB*	Others	2	6	12
103	ENSG00000196652	*ZKSCAN5*	zf-C2H2	2	6	12
104	ENSG00000010244	*ZNF207*	Others	2	6	11
105	ENSG00000067082	*KLF6*	zf-C2H2	2	6	11
106	ENSG00000153207	*AHCTF1*	Others	2	6	11
107	ENSG00000197857	*ZNF44*	zf-C2H2	2	6	11
108	ENSG00000124496	*TRERF1*	zf-C2H2	2	6	11
109	ENSG00000143390	*RFX5*	RFX	2	6	11
110	ENSG00000146676	*PURB*	Others	2	6	11
111	ENSG00000185022	*MAFF*	TF_bZIP	2	6	11
112	ENSG00000178177	*LCORL*	HTH	2	6	10
113	ENSG00000065978	*YBX1*	CSD	2	6	10
114	ENSG00000110851	*PRDM4*	zf-C2H2	2	6	10
115	ENSG00000118922	*KLF12*	zf-C2H2	2	6	10
116	ENSG00000073584	*SMARCE1*	HMG	2	6	10
117	ENSG00000256229	*ZNF486*	zf-C2H2	2	6	10
118	ENSG00000125740	*FOSB*	TF_bZIP	2	7	12
119	ENSG00000167562	*ZNF701*	zf-C2H2	2	7	12
120	ENSG00000167766	*ZNF83*	zf-C2H2	2	7	12
121	ENSG00000175322	*ZNF519*	zf-C2H2	2	7	12
122	ENSG00000185697	*MYBL1*	MYB	2	7	12
123	ENSG00000169714	*CNBP*	Others	2	7	12
124	ENSG00000213967	*ZNF726*	zf-C2H2	2	7	12
125	ENSG00000052850	*ALX4*	Homeobox	2	7	11
126	ENSG00000159556	*ISL2*	Homeobox	2	7	11
127	ENSG00000108001	*EBF3*	COE	2	7	11
128	ENSG00000236287	*ZBED5*	zf-BED	2	7	11
129	ENSG00000129534	*MIS18BP1*	MYB	2	7	10
130	ENSG00000134046	*MBD2*	MBD	2	7	10
131	ENSG00000188295	*ZNF669*	zf-C2H2	2	7	10
132	ENSG00000213988	*ZNF90*	zf-C2H2	2	7	10
133	ENSG00000213793	*ZNF888*	zf-C2H2	2	7	9
134	ENSG00000198342	*ZNF442*	zf-C2H2	2	8	12
135	ENSG00000198298	*ZNF485*	zf-C2H2	2	8	11
136	ENSG00000267041	*ZNF850*	zf-C2H2	2	8	11
137	ENSG00000125482	*TTF1*	MYB	2	8	11
138	ENSG00000198039	*ZNF273*	zf-C2H2	2	8	10
139	ENSG00000141956	*PRDM15*	zf-C2H2	2	8	9
140	ENSG00000172273	*HINFP*	zf-C2H2	2	9	12
141	ENSG00000132170	*PPARG*	THR-like	2	9	12
142	ENSG00000005436	*GCFC2*	GCFC	2	9	11
143	ENSG00000181450	*ZNF678*	zf-C2H2	2	9	11
144	ENSG00000157557	*ETS2*	ETS	2	9	10
145	ENSG00000188313	*PLSCR1*	Others	2	9	10
146	ENSG00000204366	*ZBTB12*	ZBTB	2	10	11
147	ENSG00000197905	*TEAD4*	TEA	3	5	12
148	ENSG00000152804	*HHEX*	Homeobox	3	5	11
149	ENSG00000112365	*ZBTB24*	ZBTB	3	5	10
150	ENSG00000170100	*ZNF778*	zf-C2H2	3	5	9
151	ENSG00000186230	*ZNF749*	zf-C2H2	3	5	9
152	ENSG00000185630	*PBX1*	Homeobox	3	5	8
153	ENSG00000005801	*ZNF195*	zf-C2H2	3	6	11
154	ENSG00000160352	*ZNF714*	zf-C2H2	3	6	11
155	ENSG00000204519	*ZNF551*	zf-C2H2	3	6	11
156	ENSG00000057657	*PRDM1*	zf-C2H2	3	6	11
157	ENSG00000140987	*ZSCAN32*	zf-C2H2	3	6	11
158	ENSG00000153560	*UBP1*	CP2	3	6	11
159	ENSG00000173473	*SMARCC1*	MYB	3	6	11
160	ENSG00000166949	*SMAD3*	MH1	3	6	11
161	ENSG00000204514	*ZNF814*	zf-C2H2	3	6	11
162	ENSG00000131788	*PIAS3*	zf-MIZ	3	6	10
163	ENSG00000143067	*ZNF697*	zf-C2H2	3	6	10
164	ENSG00000186660	*ZFP91*	zf-C2H2	3	6	10
165	ENSG00000214575	*CPEB1*	Others	3	6	10
166	ENSG00000065970	*FOXJ2*	Fork_head	3	6	10
167	ENSG00000067646	*ZFY*	zf-C2H2	3	6	10
168	ENSG00000117475	*BLZF1*	Others	3	6	10
169	ENSG00000118217	*ATF6*	TF_bZIP	3	6	10
170	ENSG00000269343	*ZNF587B*	zf-C2H2	3	6	10
171	ENSG00000197928	*ZNF677*	zf-C2H2	3	6	10
172	ENSG00000173011	*TADA2B*	MYB	3	6	9
173	ENSG00000136997	*MYC*	bHLH	3	6	8
174	ENSG00000135899	*SP110*	SAND	3	7	12
175	ENSG00000145220	*LYAR*	Others	3	7	12
176	ENSG00000064933	*PMS1*	HMG	3	7	12
177	ENSG00000126456	*IRF3*	IRF	3	7	12
178	ENSG00000019549	*SNAI2*	zf-C2H2	3	7	11
179	ENSG00000029363	*BCLAF1*	Others	3	7	11
180	ENSG00000172466	*ZNF24*	zf-C2H2	3	7	11
181	ENSG00000136169	*SETDB2*	MBD	3	7	11
182	ENSG00000067955	*CBFB*	CBF	3	7	10
183	ENSG00000105497	*ZNF175*	zf-C2H2	3	7	10
184	ENSG00000134852	*CLOCK*	bHLH	3	7	10
185	ENSG00000171467	*ZNF318*	Others	3	7	10

186	ENSG00000075407	*ZNF37A*	zf-C2H2	3	7	10
187	ENSG00000116793	*PHTF1*	Others	3	7	10
188	ENSG00000140548	*ZNF710*	zf-C2H2	3	7	10
189	ENSG00000141027	*NCOR1*	MYB	3	7	10
190	ENSG00000169955	*ZNF747*	zf-C2H2	3	7	10
191	ENSG00000185947	*ZNF267*	zf-C2H2	3	7	10
192	ENSG00000186416	*NKRF*	Others	3	7	10
193	ENSG00000196247	*ZNF107*	zf-C2H2	3	7	10
194	ENSG00000197933	*ZNF823*	zf-C2H2	3	7	10
195	ENSG00000198300	*n_a*	zf-C2H2	3	7	10
196	ENSG00000198482	*ZNF808*	zf-C2H2	3	7	10
197	ENSG00000245848	*CEBPA*	TF_bZIP	3	7	10
198	ENSG00000066422	*ZBTB11*	ZBTB	3	7	9
199	ENSG00000172262	*ZNF131*	ZBTB	3	7	9
200	ENSG00000081189	*MEF2C*	SRF	3	7	9
201	ENSG00000123933	*MXD4*	bHLH	3	7	9
202	ENSG00000144655	*CSRNP1*	CSRNP_N	3	7	9
203	ENSG00000090447	*TFAP4*	bHLH	3	8	12
204	ENSG00000179965	*ZNF771*	zf-C2H2	3	8	12
205	ENSG00000116044	*NFE2L2*	TF_bZIP	3	8	11
206	ENSG00000127124	*HIVEP3*	zf-C2H2	3	8	11
207	ENSG00000093167	*LRRFIP2*	LRRFIP	3	8	11
208	ENSG00000102974	*CTCF*	zf-C2H2	3	8	11
209	ENSG00000112242	*E2F3*	E2F	3	8	11
210	ENSG00000127152	*BCL11B*	zf-C2H2	3	8	11
211	ENSG00000147601	*TERF1*	MYB	3	8	11
212	ENSG00000163939	*PBRM1*	HMG	3	8	11
213	ENSG00000196345	*ZKSCAN7*	zf-C2H2	3	8	11
214	ENSG00000131848	*ZSCAN5A*	zf-C2H2	3	8	11
215	ENSG00000129535	*NRL*	TF_bZIP	3	8	10
216	ENSG00000171649	*ZIK1*	zf-C2H2	3	8	10
217	ENSG00000185252	*ZNF74*	zf-C2H2	3	8	10
218	ENSG00000096401	*CDC5L*	MYB	3	8	10
219	ENSG00000105996	*HOXA2*	Homeobox	3	8	10
220	ENSG00000119042	*SATB2*	CUT	3	8	10
221	ENSG00000158805	*ZNF276*	zf-C2H2	3	8	10
222	ENSG00000186446	*ZNF501*	zf-C2H2	3	8	10
223	ENSG00000160199	*PKNOX1*	Homeobox	3	8	10
224	ENSG00000198131	*ZNF544*	zf-C2H2	3	8	10
225	ENSG00000011451	*WIZ*	zf-C2H2	3	8	9
226	ENSG00000196757	*ZNF700*	zf-C2H2	3	8	9
227	ENSG00000204531	*POU5F1*	Pou	3	8	7
228	ENSG00000104903	*LYL1*	bHLH	3	9	12
229	ENSG00000196458	*ZNF605*	zf-C2H2	3	9	12
230	ENSG00000197343	*ZNF655*	zf-C2H2	3	9	12
231	ENSG00000162664	*ZNF326*	Others	3	9	11
232	ENSG00000072736	*NFATC3*	RHD	3	9	11
233	ENSG00000102935	*ZNF423*	zf-C2H2	3	9	11
234	ENSG00000130584	*ZBTB46*	ZBTB	3	9	11
235	ENSG00000180938	*ZNF572*	zf-C2H2	3	9	11
236	ENSG00000140262	*TCF12*	bHLH	3	9	11
237	ENSG00000156853	*ZNF689*	zf-C2H2	3	9	11
238	ENSG00000250312	*ZNF718*	zf-C2H2	3	9	11
239	ENSG00000179348	*GATA2*	zf-GATA	3	9	11
240	ENSG00000115355	*CCDC88A*	TF_bZIP	3	9	10
241	ENSG00000147180	*ZNF711*	zf-C2H2	3	9	10
242	ENSG00000175745	*NR2F1*	RXR-like	3	9	10
243	ENSG00000234444	*ZNF736*	zf-C2H2	3	9	10
244	ENSG00000277462	*ZNF670*	zf-C2H2	3	9	10
245	ENSG00000153896	*ZNF599*	zf-C2H2	3	9	10
246	ENSG00000185404	*SP140L*	SAND	3	9	9
247	ENSG00000167034	*NKX3-1*	Homeobox	3	9	8
248	ENSG00000145908	*ZNF300*	zf-C2H2	3	10	11
249	ENSG00000128652	*HOXD3*	Homeobox	3	10	10
250	ENSG00000137492	*THAP12*	THAP	3	10	10
251	ENSG00000169594	*BNC1*	zf-C2H2	3	10	10
252	ENSG00000089335	*ZNF302*	zf-C2H2	3	11	11
253	ENSG00000187801	*ZFP69B*	zf-C2H2	3	11	9
254	ENSG00000141510	*TP53*	P53	4	5	10
255	ENSG00000135457	*TFCP2*	CP2	4	6	10
256	ENSG00000213762	*ZNF134*	zf-C2H2	4	6	10
257	ENSG00000171735	*CAMTA1*	CG-1	4	6	10
258	ENSG00000197114	*ZGPAT*	Others	4	6	10
259	ENSG00000198081	*ZBTB14*	ZBTB	4	7	11
260	ENSG00000130338	*TULP4*	Tub	4	7	11
261	ENSG00000101665	*SMAD7*	MH1	4	7	10
262	ENSG00000131931	*THAP1*	THAP	4	7	10
263	ENSG00000107175	*CREB3*	TF_bZIP	4	7	10
264	ENSG00000091831	*ESR1*	ESR-like	4	7	10
265	ENSG00000092607	*TBX15*	T-box	4	7	9
266	ENSG00000164330	*EBF1*	COE	4	7	9
267	ENSG00000166860	*ZBTB39*	ZBTB	4	7	9
268	ENSG00000168310	*IRF2*	IRF	4	7	9
269	ENSG00000143578	*CREB3L4*	TF_bZIP	4	7	9
270	ENSG00000258405	*ZNF578*	zf-C2H2	4	7	9
271	ENSG00000177508	*IRX3*	Homeobox	4	7	9
272	ENSG00000179981	*TSHZ1*	zf-C2H2	4	7	8
273	ENSG00000141905	*NFIC*	CTF/NFI	4	7	7
274	ENSG00000167981	*ZNF597*	zf-C2H2	4	8	12
275	ENSG00000188321	*ZNF559*	zf-C2H2	4	8	12
276	ENSG00000104856	*RELB*	RHD	4	8	11
277	ENSG00000108175	*ZMIZ1*	zf-MIZ	4	8	11
278	ENSG00000170365	*SMAD1*	MH1	4	8	11
279	ENSG00000172748	*ZNF596*	zf-C2H2	4	8	11

280	ENSG00000173451	*THAP2*	THAP	4	8	11
281	ENSG00000059728	*MXD1*	bHLH	4	8	11
282	ENSG00000149054	*ZNF215*	zf-C2H2	4	8	11
283	ENSG00000171161	*ZNF672*	zf-C2H2	4	8	11
284	ENSG00000163132	*MSX1*	Homeobox	4	8	11
285	ENSG00000167074	*TEF*	TF_bZIP	4	8	11
286	ENSG00000141644	*MBD1*	MBD	4	8	11
287	ENSG00000113580	*NR3C1*	ESR-like	4	8	10
288	ENSG00000126746	*ZNF384*	zf-C2H2	4	8	10
289	ENSG00000136451	*VEZF1*	zf-C2H2	4	8	10
290	ENSG00000177485	*ZBTB33*	ZBTB	4	8	10
291	ENSG00000181896	*ZNF101*	zf-C2H2	4	8	10
292	ENSG00000198466	*ZNF587*	zf-C2H2	4	8	10
293	ENSG00000168661	*ZNF30*	zf-C2H2	4	8	10
294	ENSG00000196418	*ZNF124*	zf-C2H2	4	8	10
295	ENSG00000129911	*KLF16*	zf-C2H2	4	8	10
296	ENSG00000111087	*GLI1*	zf-C2H2	4	8	10
297	ENSG00000117036	*ETV3*	ETS	4	8	9
298	ENSG00000120690	*ELF1*	ETS	4	8	9
299	ENSG00000167528	*ZNF641*	zf-C2H2	4	8	9
300	ENSG00000172845	*SP3*	zf-C2H2	4	8	9
301	ENSG00000197020	*ZNF100*	zf-C2H2	4	8	9
302	ENSG00000198521	*ZNF43*	zf-C2H2	4	8	9
303	ENSG00000230797	*YY2*	zf-C2H2	4	8	9
304	ENSG00000025156	*HSF2*	HSF	4	8	9
305	ENSG00000149050	*ZNF214*	zf-C2H2	4	8	9
306	ENSG00000167635	*ZNF146*	zf-C2H2	4	8	9
307	ENSG00000064195	*n_a*	Homeobox	4	8	9
308	ENSG00000170345	*FOS*	TF_bZIP	4	8	9
309	ENSG00000181638	*ZFP41*	zf-C2H2	4	8	8
310	ENSG00000010818	*HIVEP2*	zf-C2H2	4	8	8
311	ENSG00000100281	*HMGXB4*	HMG	4	9	12
312	ENSG00000006194	*ZNF263*	zf-C2H2	4	9	12
313	ENSG00000177853	*ZNF518A*	zf-C2H2	4	9	11
314	ENSG00000077092	*RARB*	THR-like	4	9	11
315	ENSG00000168152	*THAP9*	THAP	4	9	11
316	ENSG00000170802	*FOXN2*	Fork_head	4	9	11
317	ENSG00000158691	*ZSCAN12*	zf-C2H2	4	9	11
318	ENSG00000029639	*TFB1M*	Others	4	9	11
319	ENSG00000070444	*MNT*	bHLH	4	9	11
320	ENSG00000197362	*ZNF786*	zf-C2H2	4	9	11
321	ENSG00000110925	*CSRNP2*	CSRNP_N	4	9	10
322	ENSG00000033800	*PIAS1*	zf-MIZ	4	9	10
323	ENSG00000048405	*ZNF800*	zf-C2H2	4	9	10
324	ENSG00000049618	*ARID1B*	ARID	4	9	10
325	ENSG00000084093	*REST*	zf-C2H2	4	9	10
326	ENSG00000123268	*ATF1*	TF_bZIP	4	9	10
327	ENSG00000124459	*ZNF45*	zf-C2H2	4	9	10
328	ENSG00000126767	*ELK1*	ETS	4	9	10
329	ENSG00000171940	*ZNF217*	zf-C2H2	4	9	10
330	ENSG00000177311	*ZBTB38*	ZBTB	4	9	10
331	ENSG00000185591	*SP1*	zf-C2H2	4	9	10
332	ENSG00000196628	*TCF4*	bHLH	4	9	10
333	ENSG00000198464	*ZNF480*	zf-C2H2	4	9	10
334	ENSG00000198551	*ZNF627*	zf-C2H2	4	9	10
335	ENSG00000215421	*ZNF407*	zf-C2H2	4	9	10
336	ENSG00000101544	*ADNP2*	Others	4	9	10
337	ENSG00000103449	*SALL1*	zf-C2H2	4	9	10
338	ENSG00000196437	*ZNF569*	zf-C2H2	4	9	10
339	ENSG00000197128	*ZNF772*	zf-C2H2	4	9	10
340	ENSG00000074047	*GLI2*	zf-C2H2	4	9	10
341	ENSG00000128000	*ZNF780B*	zf-C2H2	4	9	10
342	ENSG00000142528	*ZNF473*	zf-C2H2	4	9	10
343	ENSG00000172789	*HOXC5*	Homeobox	4	9	10
344	ENSG00000167182	*SP2*	zf-C2H2	4	9	9
345	ENSG00000147124	*ZNF41*	zf-C2H2	4	9	9
346	ENSG00000178163	*ZNF518B*	zf-C2H2	4	9	9
347	ENSG00000180357	*ZNF609*	Others	4	9	9
348	ENSG00000196110	*ZNF699*	zf-C2H2	4	9	9
349	ENSG00000198346	*ZNF813*	zf-C2H2	4	9	9
350	ENSG00000198815	*FOXJ3*	Fork_head	4	9	9
351	ENSG00000104064	*GABPB1*	Others	4	9	9
352	ENSG00000106052	*TAX1BP1*	Others	4	9	9
353	ENSG00000115415	*STAT1*	STAT	4	9	9
354	ENSG00000120093	*HOXB3*	Homeobox	4	9	9
355	ENSG00000137871	*ZNF280D*	Others	4	9	9
356	ENSG00000170448	*NFXL1*	zf-NF-X1	4	9	9
357	ENSG00000204524	*ZNF805*	zf-C2H2	4	9	9
358	ENSG00000070476	*ZXDC*	zf-C2H2	4	9	8
359	ENSG00000100644	*HIF1A*	Others	4	9	8
360	ENSG00000120693	*SMAD9*	MH1	4	9	8
361	ENSG00000165259	*HDX*	Homeobox	4	9	8
362	ENSG00000171970	*ZNF57*	zf-C2H2	4	9	8
363	ENSG00000056277	*ZNF280C*	Others	4	9	7
364	ENSG00000118620	*ZNF430*	zf-C2H2	4	9	7
365	ENSG00000010539	*ZNF200*	zf-C2H2	4	10	11
366	ENSG00000054267	*ARID4B*	ARID	4	10	11
367	ENSG00000078246	*TULP3*	Tub	4	10	11
368	ENSG00000113658	*SMAD5*	MH1	4	10	11
369	ENSG00000142556	*ZNF614*	zf-C2H2	4	10	11
370	ENSG00000102034	*ELF4*	ETS	4	10	11
371	ENSG00000050344	*NFE2L3*	TF_bZIP	4	10	11
372	ENSG00000100426	*ZBED4*	zf-BED	4	10	10
373	ENSG00000121417	*ZNF211*	zf-C2H2	4	10	10

374	ENSG00000152443	*ZNF776*	zf-C2H2	4	10	10
375	ENSG00000167384	*ZNF180*	zf-C2H2	4	10	10
376	ENSG00000100625	*SIX4*	Homeobox	4	10	10
377	ENSG00000146757	*ZNF92*	zf-C2H2	4	10	10
378	ENSG00000159140	*SON*	Others	4	10	10
379	ENSG00000077150	*NFKB2*	RHD	4	10	10
380	ENSG00000170561	*IRX2*	Homeobox	4	10	10
381	ENSG00000085274	*MYNN*	ZBTB	4	10	9
382	ENSG00000120075	*HOXB5*	Homeobox	4	10	9
383	ENSG00000171606	*ZNF274*	zf-C2H2	4	10	9
384	ENSG00000182986	*ZNF320*	zf-C2H2	4	10	9
385	ENSG00000198026	*ZNF335*	zf-C2H2	4	10	9
386	ENSG00000275004	*ZNF280B*	Others	4	10	9
387	ENSG00000106546	*AHR*	bHLH	4	10	9
388	ENSG00000136866	*ZFP37*	zf-C2H2	4	10	9
389	ENSG00000167785	*ZNF558*	zf-C2H2	4	10	9
390	ENSG00000174963	*ZIC4*	zf-C2H2	4	10	9
391	ENSG00000198963	*RORB*	THR-like	4	10	9
392	ENSG00000101126	*ADNP*	Homeobox	4	10	8
393	ENSG00000147118	*ZNF182*	zf-C2H2	4	10	8
394	ENSG00000176542	*USF3*	bHLH	4	10	8
395	ENSG00000036549	*n_a*	MYB	4	10	8
396	ENSG00000168813	*ZNF507*	zf-C2H2	4	10	8
397	ENSG00000170322	*NFRKB*	Others	4	10	8
398	ENSG00000177463	*NR2C2*	RXR-like	4	10	8
399	ENSG00000117713	*ARID1A*	ARID	4	10	7
400	ENSG00000140382	*HMG20A*	HMG	4	10	7
401	ENSG00000263001	*GTF2I*	GTF2I	4	10	7
402	ENSG00000174796	*THAP6*	THAP	4	11	11
403	ENSG00000177888	*ZBTB41*	ZBTB	4	11	11
404	ENSG00000086102	*NFX1*	zf-NF-X1	4	11	10
405	ENSG00000155090	*KLF10*	zf-C2H2	4	11	10
406	ENSG00000172888	*ZNF621*	zf-C2H2	4	11	10
407	ENSG00000188227	*ZNF793*	zf-C2H2	4	11	10
408	ENSG00000124201	*ZNFX1*	Others	4	11	9
409	ENSG00000117625	*RCOR3*	MYB	4	11	9
410	ENSG00000162601	*MYSM1*	MYB	4	11	9
411	ENSG00000196417	*ZNF765*	zf-C2H2	4	11	9
412	ENSG00000198538	*ZNF28*	zf-C2H2	4	11	9
413	ENSG00000152439	*ZNF773*	zf-C2H2	4	11	8
414	ENSG00000243678	*NME2*	Others	5	6	10
415	ENSG00000105991	*HOXA1*	Homeobox	5	6	9
416	ENSG00000108509	*CAMTA2*	CG-1	5	6	8
417	ENSG00000205250	*E2F4*	E2F	5	7	12
418	ENSG00000251369	*ZNF550*	zf-C2H2	5	7	11
419	ENSG00000276234	*TADA2A*	MYB	5	7	11
420	ENSG00000185869	*ZNF829*	zf-C2H2	5	7	11
421	ENSG00000105717	*PBX4*	Homeobox	5	7	10
422	ENSG00000141002	*TCF25*	Others	5	7	10
423	ENSG00000125952	*MAX*	bHLH	5	7	10
424	ENSG00000176946	*THAP4*	THAP	5	7	10
425	ENSG00000196653	*ZNF502*	zf-C2H2	5	7	10
426	ENSG00000178386	*n_a*	zf-C2H2	5	7	9
427	ENSG00000155966	*AFF2*	AF-4	5	7	9
428	ENSG00000153879	*CEBPG*	TF_bZIP	5	7	9
429	ENSG00000197937	*ZNF347*	zf-C2H2	5	7	8
430	ENSG00000121406	*ZNF549*	zf-C2H2	5	7	8
431	ENSG00000188070	*C11orf95*	zf-BED	5	7	8
432	ENSG00000256771	*ZNF253*	zf-C2H2	5	7	7
433	ENSG00000105732	*ZNF574*	zf-C2H2	5	8	12
434	ENSG00000085276	*MECOM*	zf-C2H2	5	8	11
435	ENSG00000122592	*HOXA7*	Homeobox	5	8	11
436	ENSG00000178187	*ZNF454*	zf-C2H2	5	8	11
437	ENSG00000197008	*ZNF138*	zf-C2H2	5	8	11
438	ENSG00000136826	*KLF4*	zf-C2H2	5	8	11
439	ENSG00000167380	*ZNF226*	zf-C2H2	5	8	11
440	ENSG00000178662	*CSRNP3*	CSRNP_N	5	8	10
441	ENSG00000169136	*ATF5*	TF_bZIP	5	8	10
442	ENSG00000124813	*RUNX2*	Runt	5	8	9
443	ENSG00000124613	*ZNF391*	zf-C2H2	5	8	9
444	ENSG00000119725	*ZNF410*	zf-C2H2	5	8	9
445	ENSG00000125812	*GZF1*	ZBTB	5	8	9
446	ENSG00000169554	*ZEB2*	zf-C2H2	5	8	8
447	ENSG00000136770	*DNAJC1*	MYB	5	8	8
448	ENSG00000180884	*ZNF792*	zf-C2H2	5	8	8
449	ENSG00000186376	*ZNF75D*	zf-C2H2	5	8	8
450	ENSG00000130856	*ZNF236*	zf-C2H2	5	8	7
451	ENSG00000113716	*HMGXB3*	HMG	5	8	6
452	ENSG00000064961	*HMG20B*	HMG	5	9	11
453	ENSG00000132024	*CC2D1A*	Others	5	9	11
454	ENSG00000203326	*ZNF525*	zf-C2H2	5	9	11
455	ENSG00000196263	*ZNF471*	zf-C2H2	5	9	10
456	ENSG00000065029	*ZNF76*	zf-C2H2	5	9	10
457	ENSG00000169083	*AR*	ESR-like	5	9	10
458	ENSG00000188095	*MESP2*	bHLH	5	9	10
459	ENSG00000158773	*USF1*	bHLH	5	9	10
460	ENSG00000167967	*E4F1*	zf-C2H2	5	9	10
461	ENSG00000060138	*YBX3*	CSD	5	9	10
462	ENSG00000083838	*ZNF446*	zf-C2H2	5	9	10
463	ENSG00000106459	*NRF1*	Nrf1	5	9	10
464	ENSG00000130818	*ZNF426*	zf-C2H2	5	9	10
465	ENSG00000078403	*MLLT10*	Others	5	9	9
466	ENSG00000157657	*ZNF618*	zf-C2H2	5	9	9
467	ENSG00000158711	*ELK4*	ETS	5	9	9

468	ENSG00000213676	*ATF6B*	TF_bZIP	5	9	9
469	ENSG00000121864	*ZNF639*	zf-C2H2	5	9	9
470	ENSG00000173041	*ZNF680*	zf-C2H2	5	9	9
471	ENSG00000120963	*ZNF706*	Others	5	9	9
472	ENSG00000166888	*STAT6*	STAT	5	9	8
473	ENSG00000177599	*ZNF491*	zf-C2H2	5	9	8
474	ENSG00000178764	*ZHX2*	Homeobox	5	9	8
475	ENSG00000181315	*ZNF322*	zf-C2H2	5	9	8
476	ENSG00000198455	*ZXDB*	zf-C2H2	5	9	8
477	ENSG00000272602	*ZNF595*	zf-C2H2	5	9	8
478	ENSG00000182318	*ZSCAN22*	zf-C2H2	5	9	8
479	ENSG00000102870	*ZNF629*	zf-C2H2	5	9	8
480	ENSG00000196453	*ZNF777*	zf-C2H2	5	9	8
481	ENSG00000233608	*TWIST2*	bHLH	5	9	8
482	ENSG00000144747	*TMF1*	Others	5	9	7
483	ENSG00000184677	*ZBTB40*	ZBTB	5	9	7
484	ENSG00000116731	*PRDM2*	zf-C2H2	5	9	7
485	ENSG00000185551	*NR2F2*	RXR-like	5	9	7
486	ENSG00000181220	*ZNF746*	zf-C2H2	5	10	11
487	ENSG00000186300	*ZNF555*	zf-C2H2	5	10	11
488	ENSG00000167840	*ZNF232*	zf-C2H2	5	10	11
489	ENSG00000171574	*ZNF584*	zf-C2H2	5	10	11
490	ENSG00000170325	*PRDM10*	zf-C2H2	5	10	10
491	ENSG00000186564	*FOXD2*	Fork_head	5	10	10
492	ENSG00000214029	*ZNF891*	zf-C2H2	5	10	10
493	ENSG00000181894	*ZNF329*	zf-C2H2	5	10	10
494	ENSG00000187626	*ZKSCAN4*	zf-C2H2	5	10	10
495	ENSG00000198546	*ZNF511*	Others	5	10	10
496	ENSG00000221923	*ZNF880*	zf-C2H2	5	10	10
497	ENSG00000072364	*AFF4*	AF-4	5	10	9
498	ENSG00000104447	*TRPS1*	zf-GATA	5	10	9
499	ENSG00000118495	*PLAGL1*	zf-C2H2	5	10	9
500	ENSG00000151612	*ZNF827*	zf-C2H2	5	10	9
501	ENSG00000183309	*ZNF623*	zf-C2H2	5	10	9
502	ENSG00000123411	*IKZF4*	zf-C2H2	5	10	9
503	ENSG00000134107	*BHLHE40*	bHLH	5	10	9
504	ENSG00000155545	*MIER3*	MYB	5	10	9
505	ENSG00000177045	*SIX5*	Homeobox	5	10	9
506	ENSG00000196705	*ZNF431*	zf-C2H2	5	10	9
507	ENSG00000198169	*ZNF251*	zf-C2H2	5	10	9
508	ENSG00000078043	*PIAS2*	zf-MIZ	5	10	9
509	ENSG00000120784	*ZFP30*	zf-C2H2	5	10	9
510	ENSG00000175879	*HOXD8*	Homeobox	5	10	9
511	ENSG00000235109	*ZSCAN31*	zf-C2H2	5	10	9
512	ENSG00000109381	*ELF2*	ETS	5	10	8
513	ENSG00000123636	*BAZ2B*	MBD	5	10	8
514	ENSG00000165156	*ZHX1*	Homeobox	5	10	8
515	ENSG00000185129	*PURA*	Others	5	10	8
516	ENSG00000188786	*MTF1*	zf-C2H2	5	10	8
517	ENSG00000189042	*ZNF567*	zf-C2H2	5	10	8
518	ENSG00000095574	*IKZF5*	zf-C2H2	5	10	8
519	ENSG00000102804	*TSC22D1*	TSC22	5	10	8
520	ENSG00000126804	*ZBTB1*	ZBTB	5	10	8
521	ENSG00000138738	*PRDM5*	zf-C2H2	5	10	8
522	ENSG00000162086	*ZNF75A*	zf-C2H2	5	10	8
523	ENSG00000180626	*ZNF594*	zf-C2H2	5	10	8
524	ENSG00000196428	*TSC22D2*	TSC22	5	10	8
525	ENSG00000197841	*ZNF181*	zf-C2H2	5	10	8
526	ENSG00000140265	*ZSCAN29*	zf-C2H2	5	10	8
527	ENSG00000166478	*ZNF143*	zf-C2H2	5	10	8
528	ENSG00000197647	*ZNF433*	zf-C2H2	5	10	8
529	ENSG00000160908	*ZNF394*	zf-C2H2	5	10	8
530	ENSG00000141646	*SMAD4*	MH1	5	10	7
531	ENSG00000169926	*KLF13*	zf-C2H2	5	10	7
532	ENSG00000198146	*ZNF770*	zf-C2H2	5	10	7
533	ENSG00000115966	*ATF2*	TF_bZIP	5	10	7
534	ENSG00000131845	*ZNF304*	zf-C2H2	5	10	7
535	ENSG00000148516	*ZEB1*	zf-C2H2	5	10	7
536	ENSG00000163848	*ZNF148*	zf-C2H2	5	10	7
537	ENSG00000196693	*ZNF33B*	zf-C2H2	5	10	7
538	ENSG00000176024	*ZNF613*	zf-C2H2	5	10	7
539	ENSG00000180855	*ZNF443*	zf-C2H2	5	10	7
540	ENSG00000197372	*ZNF675*	zf-C2H2	5	10	7
541	ENSG00000095951	*HIVEP1*	zf-C2H2	5	10	6
542	ENSG00000114439	*BBX*	HMG	5	10	6
543	ENSG00000198160	*MIER1*	MYB	5	10	6
544	ENSG00000135164	*DMTF1*	MYB	5	10	6
545	ENSG00000213020	*ZNF611*	zf-C2H2	5	11	11
546	ENSG00000131849	*ZNF132*	zf-C2H2	5	11	11
547	ENSG00000175787	*ZNF169*	zf-C2H2	5	11	11
548	ENSG00000138380	*CARF*	Others	5	11	10
549	ENSG00000173276	*ZBTB21*	ZBTB	5	11	10
550	ENSG00000076108	*BAZ2A*	MBD	5	11	9
551	ENSG00000116580	*GON4L*	Others	5	11	9
552	ENSG00000121903	*ZSCAN20*	zf-C2H2	5	11	9
553	ENSG00000196357	*ZNF565*	zf-C2H2	5	11	9
554	ENSG00000197497	*ZNF665*	zf-C2H2	5	11	9
555	ENSG00000197782	*ZNF780A*	zf-C2H2	5	11	9
556	ENSG00000081386	*ZNF510*	zf-C2H2	5	11	9
557	ENSG00000102908	*NFAT5*	RHD	5	11	9
558	ENSG00000122482	*ZNF644*	zf-C2H2	5	11	9
559	ENSG00000174197	*MGA*	T-box	5	11	9
560	ENSG00000177683	*THAP5*	THAP	5	11	9
561	ENSG00000178338	*ZNF354B*	zf-C2H2	5	11	9

562	ENSG00000179195	*ZNF664*	zf-C2H2	5	11	9
563	ENSG00000186814	*ZSCAN30*	zf-C2H2	5	11	9
564	ENSG00000196268	*ZNF493*	zf-C2H2	5	11	9
565	ENSG00000204604	*ZNF468*	zf-C2H2	5	11	9
566	ENSG00000089902	*RCOR1*	MYB	5	11	8
567	ENSG00000118260	*CREB1*	TF_bZIP	5	11	8
568	ENSG00000161551	*ZNF577*	zf-C2H2	5	11	7
569	ENSG00000109320	*NFKB1*	RHD	5	11	7
570	ENSG00000115568	*ZNF142*	zf-C2H2	5	11	7
571	ENSG00000124782	*RREB1*	zf-C2H2	5	11	7
572	ENSG00000168769	*TET2*	Others	5	11	7
573	ENSG00000256294	*ZNF225*	zf-C2H2	5	11	7
574	ENSG00000177932	*ZNF354C*	zf-C2H2	5	11	6
575	ENSG00000133794	*ARNTL*	bHLH	5	12	8
576	ENSG00000154727	*GABPA*	ETS	5	12	8
577	ENSG00000156030	*ELMSAN1*	Others	5	12	8
578	ENSG00000186951	*PPARA*	THR-like	5	12	8
579	ENSG00000197779	*ZNF81*	zf-C2H2	5	12	8
580	ENSG00000106006	*HOXA6*	Homeobox	5	12	6
581	ENSG00000196267	*ZNF836*	zf-C2H2	6	7	8
582	ENSG00000159086	*PAXBP1*	GCFC	6	7	8
583	ENSG00000227124	*ZNF717*	zf-C2H2	6	7	8
584	ENSG00000165684	*SNAPC4*	MYB	6	8	11
585	ENSG00000100105	*PATZ1*	ZBTB	6	8	10
586	ENSG00000166261	*ZNF202*	zf-C2H2	6	8	10
587	ENSG00000187815	*ZFP69*	zf-C2H2	6	8	10
588	ENSG00000176222	*ZNF404*	zf-C2H2	6	8	9
589	ENSG00000125826	*RBCK1*	Others	6	8	8
590	ENSG00000197050	*ZNF420*	zf-C2H2	6	8	8
591	ENSG00000096654	*ZNF184*	zf-C2H2	6	8	8
592	ENSG00000186017	*ZNF566*	zf-C2H2	6	8	8
593	ENSG00000079432	*CIC*	HMG	6	8	7
594	ENSG00000186272	*ZNF17*	zf-C2H2	6	8	7
595	ENSG00000178935	*ZNF552*	zf-C2H2	6	8	6
596	ENSG00000166925	*TSC22D4*	TSC22	6	9	11
597	ENSG00000109705	*NKX3-2*	Homeobox	6	9	11
598	ENSG00000221994	*ZNF630*	zf-C2H2	6	9	10
599	ENSG00000189190	*ZNF600*	zf-C2H2	6	9	10
600	ENSG00000196172	*ZNF681*	zf-C2H2	6	9	10
601	ENSG00000123388	*HOXC11*	Homeobox	6	9	10
602	ENSG00000064490	*RFXANK*	Others	6	9	9
603	ENSG00000256087	*ZNF432*	zf-C2H2	6	9	9
604	ENSG00000151500	*THYN1*	Others	6	9	9
605	ENSG00000169016	*E2F6*	E2F	6	9	9
606	ENSG00000171443	*ZNF524*	zf-C2H2	6	9	9
607	ENSG00000174332	*GLIS1*	zf-C2H2	6	9	9
608	ENSG00000188290	*HES4*	bHLH	6	9	9
609	ENSG00000198205	*ZXDA*	zf-C2H2	6	9	9
610	ENSG00000179528	*LBX2*	Homeobox	6	9	9
611	ENSG00000127528	*KLF2*	zf-C2H2	6	9	9
612	ENSG00000163795	*ZNF513*	zf-C2H2	6	9	9
613	ENSG00000169946	*ZFPM2*	zf-C2H2	6	9	9
614	ENSG00000180818	*HOXC10*	Homeobox	6	9	8
615	ENSG00000185278	*ZBTB37*	ZBTB	6	9	8
616	ENSG00000198795	*ZNF521*	zf-C2H2	6	9	8
617	ENSG00000183621	*ZNF438*	zf-C2H2	6	9	8
618	ENSG00000179632	*MAF1*	Others	6	9	8
619	ENSG00000167625	*ZNF526*	zf-C2H2	6	9	8
620	ENSG00000170260	*ZNF212*	zf-C2H2	6	9	8
621	ENSG00000187098	*MITF*	bHLH	6	9	8
622	ENSG00000018869	*ZNF582*	zf-C2H2	6	9	8
623	ENSG00000106571	*GLI3*	zf-C2H2	6	9	8
624	ENSG00000106948	*AKNA*	Others	6	9	7
625	ENSG00000147789	*ZNF7*	zf-C2H2	6	9	7
626	ENSG00000197062	*ZSCAN26*	zf-C2H2	6	9	7
627	ENSG00000062194	*GPBP1*	Others	6	9	7
628	ENSG00000196466	*ZNF799*	zf-C2H2	6	9	7
629	ENSG00000171163	*ZNF692*	zf-C2H2	6	9	7
630	ENSG00000066827	*ZFAT*	zf-C2H2	6	9	7
631	ENSG00000213096	*ZNF254*	zf-C2H2	6	9	6
632	ENSG00000181690	*PLAG1*	zf-C2H2	6	9	6
633	ENSG00000173545	*ZNF622*	zf-C2H2	6	9	6
634	ENSG00000132604	*TERF2*	MYB	6	10	10
635	ENSG00000237440	*n_a*	zf-C2H2	6	10	10
636	ENSG00000265763	*ZNF488*	Others	6	10	10
637	ENSG00000103495	*MAZ*	zf-C2H2	6	10	10
638	ENSG00000124444	*ZNF576*	zf-C2H2	6	10	10
639	ENSG00000178860	*MSC*	bHLH	6	10	10
640	ENSG00000164048	*ZNF589*	zf-C2H2	6	10	10
641	ENSG00000133740	*E2F5*	E2F	6	10	10
642	ENSG00000171295	*ZNF440*	zf-C2H2	6	10	9
643	ENSG00000122034	*GTF3A*	zf-C2H2	6	10	9
644	ENSG00000130803	*ZNF317*	zf-C2H2	6	10	9
645	ENSG00000168779	*SHOX2*	Homeobox	6	10	9
646	ENSG00000169740	*ZNF32*	zf-C2H2	6	10	9
647	ENSG00000105136	*ZNF419*	zf-C2H2	6	10	9
648	ENSG00000175213	*ZNF408*	zf-C2H2	6	10	9
649	ENSG00000176472	*ZNF575*	zf-C2H2	6	10	9
650	ENSG00000108813	*DLX4*	Homeobox	6	10	9
651	ENSG00000137185	*ZSCAN9*	zf-C2H2	6	10	9
652	ENSG00000104177	*MYEF2*	Others	6	10	8
653	ENSG00000112033	*PPARD*	THR-like	6	10	8
654	ENSG00000186130	*ZBTB6*	ZBTB	6	10	8
655	ENSG00000196646	*ZNF136*	zf-C2H2	6	10	8

656	ENSG00000213999	*n_a*	SRF	6	10	8
657	ENSG00000142409	*ZNF787*	zf-C2H2	6	10	8
658	ENSG00000152518	*ZFP36L2*	zf-CCCH	6	10	8
659	ENSG00000243943	*ZNF512*	zf-C2H2	6	10	8
660	ENSG00000126003	*PLAGL2*	zf-C2H2	6	10	8
661	ENSG00000037965	*n_a*	Homeobox	6	10	8
662	ENSG00000185650	*ZFP36L1*	zf-CCCH	6	10	8
663	ENSG00000137504	*CREBZF*	TF_bZIP	6	10	7
664	ENSG00000170653	*ATF7*	TF_bZIP	6	10	7
665	ENSG00000142611	*PRDM16*	zf-C2H2	6	10	7
666	ENSG00000172059	*KLF11*	zf-C2H2	6	10	7
667	ENSG00000197013	*ZNF429*	zf-C2H2	6	10	7
668	ENSG00000105672	*ETV2*	ETS	6	10	7
669	ENSG00000176887	*SOX11*	HMG	6	10	7
670	ENSG00000177030	*DEAF1*	SAND	6	10	7
671	ENSG00000068305	*MEF2A*	SRF	6	10	6
672	ENSG00000139613	*SMARCC2*	MYB	6	10	6
673	ENSG00000177125	*ZBTB34*	ZBTB	6	10	6
674	ENSG00000105866	*SP4*	zf-C2H2	6	10	6
675	ENSG00000137203	*TFAP2A*	AP-2	6	10	6
676	ENSG00000198429	*ZNF69*	zf-C2H2	6	10	6
677	ENSG00000198105	*ZNF248*	zf-C2H2	6	10	5
678	ENSG00000162419	*GMEB1*	SAND	6	11	12
679	ENSG00000198945	*L3MBTL3*	Others	6	11	11
680	ENSG00000138073	*PREB*	Others	6	11	11
681	ENSG00000130544	*ZNF557*	zf-C2H2	6	11	11
682	ENSG00000178229	*ZNF543*	zf-C2H2	6	11	10
683	ENSG00000164011	*ZNF691*	zf-C2H2	6	11	10
684	ENSG00000185122	*HSF1*	HSF	6	11	10
685	ENSG00000148337	*CIZ1*	Others	6	11	10
686	ENSG00000197044	*ZNF441*	zf-C2H2	6	11	9
687	ENSG00000134954	*ETS1*	ETS	6	11	9
688	ENSG00000169155	*ZBTB43*	ZBTB	6	11	9
689	ENSG00000175105	*ZNF654*	zf-C2H2	6	11	9
690	ENSG00000197608	*ZNF841*	zf-C2H2	6	11	9
691	ENSG00000245680	*ZNF585B*	zf-C2H2	6	11	9
692	ENSG00000136630	*HLX*	Homeobox	6	11	9
693	ENSG00000159263	*SIM2*	bHLH	6	11	9
694	ENSG00000184436	*THAP7*	THAP	6	11	9
695	ENSG00000260027	*HOXB7*	Homeobox	6	11	9
696	ENSG00000069011	*PITX1*	Homeobox	6	11	9
697	ENSG00000071655	*MBD3*	MBD	6	11	9
698	ENSG00000119574	*ZBTB45*	ZBTB	6	11	9
699	ENSG00000133937	*GSC*	Homeobox	6	11	9
700	ENSG00000169957	*ZNF768*	zf-C2H2	6	11	9
701	ENSG00000185507	*IRF7*	IRF	6	11	9
702	ENSG00000109787	*KLF3*	zf-C2H2	6	11	8
703	ENSG00000151702	*FLI1*	ETS	6	11	8
704	ENSG00000041988	*THAP3*	THAP	6	11	8
705	ENSG00000121297	*TSHZ3*	zf-C2H2	6	11	8
706	ENSG00000122778	*KIAA1549*	Others	6	11	8
707	ENSG00000131115	*ZNF227*	zf-C2H2	6	11	8
708	ENSG00000173480	*ZNF417*	zf-C2H2	6	11	8
709	ENSG00000180479	*ZNF571*	zf-C2H2	6	11	8
710	ENSG00000213015	*ZNF580*	zf-C2H2	6	11	8
711	ENSG00000112561	*TFEB*	bHLH	6	11	8
712	ENSG00000133250	*ZNF414*	Others	6	11	8
713	ENSG00000167685	*ZNF444*	zf-C2H2	6	11	8
714	ENSG00000256683	*ZNF350*	zf-C2H2	6	11	8
715	ENSG00000157554	*ERG*	ETS	6	11	8
716	ENSG00000223547	*ZNF844*	zf-C2H2	6	11	8
717	ENSG00000107249	*GLIS3*	zf-C2H2	6	11	7
718	ENSG00000189079	*ARID2*	ARID	6	11	7
719	ENSG00000198185	*ZNF334*	zf-C2H2	6	11	7
720	ENSG00000008441	*NFIX*	CTF/NFI	6	11	7
721	ENSG00000067066	*SP100*	SAND	6	11	7
722	ENSG00000125945	*ZNF436*	zf-C2H2	6	11	7
723	ENSG00000134138	*MEIS2*	Homeobox	6	11	7
724	ENSG00000146587	*RBAK*	zf-C2H2	6	11	7
725	ENSG00000167555	*ZNF528*	zf-C2H2	6	11	7
726	ENSG00000186812	*ZNF397*	zf-C2H2	6	11	7
727	ENSG00000197037	*ZSCAN25*	zf-C2H2	6	11	7
728	ENSG00000257591	*ZNF625*	zf-C2H2	6	11	7
729	ENSG00000278129	*ZNF8*	zf-C2H2	6	11	7
730	ENSG00000178917	*ZNF852*	zf-C2H2	6	11	7
731	ENSG00000105856	*HBP1*	HMG	6	11	6
732	ENSG00000167232	*ZNF91*	zf-C2H2	6	11	6
733	ENSG00000159592	*GPBP1L1*	Others	6	11	6
734	ENSG00000100811	*YY1*	zf-C2H2	6	11	5
735	ENSG00000139154	*AEBP2*	zf-C2H2	6	11	5
736	ENSG00000182568	*SATB1*	CUT	6	11	5
737	ENSG00000101190	*TCFL5*	bHLH	6	11	5
738	ENSG00000092203	*TOX4*	HMG	6	12	10
739	ENSG00000102038	*SMARCA1*	MYB	6	12	9
740	ENSG00000063587	*ZNF275*	zf-C2H2	6	12	9
741	ENSG00000083844	*ZNF264*	zf-C2H2	6	12	9
742	ENSG00000181135	*ZNF707*	zf-C2H2	6	12	9
743	ENSG00000032219	*ARID4A*	ARID	6	12	8
744	ENSG00000055609	*KMT2C*	HMG	6	12	8
745	ENSG00000156273	*BACH1*	TF_bZIP	6	12	8
746	ENSG00000168916	*ZNF608*	Others	6	12	8
747	ENSG00000196233	*LCOR*	HTH	6	12	8
748	ENSG00000197619	*ZNF615*	zf-C2H2	6	12	8
749	ENSG00000254004	*ZNF260*	zf-C2H2	6	12	8

750	ENSG00000275111	*ZNF2*	zf-C2H2	6	12	8
751	ENSG00000141448	*GATA6*	zf-GATA	6	12	8
752	ENSG00000175395	*ZNF25*	zf-C2H2	6	12	8
753	ENSG00000169131	*ZNF354A*	zf-C2H2	6	12	8
754	ENSG00000118263	*KLF7*	zf-C2H2	6	12	7
755	ENSG00000213799	*ZNF845*	zf-C2H2	6	12	7
756	ENSG00000082641	*NFE2L1*	TF_bZIP	6	12	7
757	ENSG00000116016	*EPAS1*	Others	6	12	7
758	ENSG00000166402	*TUB*	Tub	6	12	7
759	ENSG00000169057	*MECP2*	MBD	6	12	7
760	ENSG00000170485	*NPAS2*	bHLH	6	12	7
761	ENSG00000173068	*BNC2*	zf-C2H2	6	12	7
762	ENSG00000173875	*ZNF791*	zf-C2H2	6	12	7
763	ENSG00000225614	*ZNF469*	zf-C2H2	6	12	7
764	ENSG00000106261	*ZKSCAN1*	zf-C2H2	6	12	7
765	ENSG00000132846	*ZBED3*	zf-BED	6	12	7
766	ENSG00000160961	*ZNF333*	zf-C2H2	6	12	7
767	ENSG00000179909	*ZNF154*	zf-C2H2	6	12	6
768	ENSG00000111269	*CREBL2*	Others	6	12	6
769	ENSG00000131127	*ZNF141*	zf-C2H2	6	12	6
770	ENSG00000139651	*ZNF740*	zf-C2H2	6	12	6
771	ENSG00000167395	*ZNF646*	zf-C2H2	6	12	6
772	ENSG00000171469	*ZNF561*	zf-C2H2	6	12	6
773	ENSG00000180787	*ZFP3*	zf-C2H2	6	12	6
774	ENSG00000188994	*ZNF292*	zf-C2H2	6	12	6
775	ENSG00000009307	*CSDE1*	CSD	6	12	5
776	ENSG00000081665	*ZNF506*	zf-C2H2	6	12	5
777	ENSG00000164463	*CREBRF*	Others	6	12	5
778	ENSG00000174306	*ZHX3*	Homeobox	6	12	4
779	ENSG00000120149	*MSX2*	Homeobox	7	7	7
780	ENSG00000090612	*ZNF268*	zf-C2H2	7	8	10
781	ENSG00000006377	*DLX6*	Homeobox	7	8	9
782	ENSG00000161298	*ZNF382*	zf-C2H2	7	8	9
783	ENSG00000087903	*RFX2*	RFX	7	8	8
784	ENSG00000132005	*RFX1*	RFX	7	8	8
785	ENSG00000116035	*VAX2*	Homeobox	7	8	6
786	ENSG00000116819	*TFAP2E*	AP-2	7	9	11
787	ENSG00000169951	*ZNF764*	zf-C2H2	7	9	10
788	ENSG00000197483	*ZNF628*	zf-C2H2	7	9	9
789	ENSG00000182742	*HOXB4*	Homeobox	7	9	9
790	ENSG00000147421	*HMBOX1*	Homeobox	7	9	9
791	ENSG00000256223	*ZNF10*	zf-C2H2	7	9	9
792	ENSG00000112837	*TBX18*	T-box	7	9	8
793	ENSG00000119138	*KLF9*	zf-C2H2	7	9	8
794	ENSG00000176293	*ZNF135*	zf-C2H2	7	9	8
795	ENSG00000196381	*ZNF781*	zf-C2H2	7	9	8
796	ENSG00000144485	*HES6*	bHLH	7	9	8
797	ENSG00000152433	*ZNF547*	zf-C2H2	7	9	8
798	ENSG00000198556	*ZNF789*	zf-C2H2	7	9	8
799	ENSG00000144792	*ZNF660*	zf-C2H2	7	9	7
800	ENSG00000197054	*ZNF763*	zf-C2H2	7	9	7
801	ENSG00000197935	*ZNF311*	zf-C2H2	7	9	7
802	ENSG00000196700	*ZNF512B*	zf-C2H2	7	9	6
803	ENSG00000020256	*ZFP64*	zf-C2H2	7	9	5
804	ENSG00000197757	*HOXC6*	Homeobox	7	9	5
805	ENSG00000167637	*ZNF283*	zf-C2H2	7	10	10
806	ENSG00000148200	*NR6A1*	GCNF-like	7	10	9
807	ENSG00000171291	*ZNF439*	zf-C2H2	7	10	9
808	ENSG00000198440	*ZNF583*	zf-C2H2	7	10	9
809	ENSG00000204304	*PBX2*	Homeobox	7	10	9
810	ENSG00000088876	*ZNF343*	zf-C2H2	7	10	9
811	ENSG00000128272	*ATF4*	TF_bZIP	7	10	9
812	ENSG00000167554	*ZNF610*	zf-C2H2	7	10	9
813	ENSG00000177732	*SOX12*	HMG	7	10	9
814	ENSG00000129194	*SOX15*	HMG	7	10	9
815	ENSG00000188171	*ZNF626*	zf-C2H2	7	10	9
816	ENSG00000143373	*ZNF687*	zf-C2H2	7	10	8
817	ENSG00000198740	*ZNF652*	zf-C2H2	7	10	8
818	ENSG00000019485	*PRDM11*	Others	7	10	8
819	ENSG00000078900	*TP73*	P53	7	10	8
820	ENSG00000116809	*ZBTB17*	ZBTB	7	10	8
821	ENSG00000140836	*ZFHX3*	Homeobox	7	10	8
822	ENSG00000166823	*MESP1*	bHLH	7	10	8
823	ENSG00000263002	*ZNF234*	zf-C2H2	7	10	8
824	ENSG00000154957	*ZNF18*	zf-C2H2	7	10	8
825	ENSG00000170549	*IRX1*	Homeobox	7	10	8
826	ENSG00000162714	*ZNF496*	zf-C2H2	7	10	8
827	ENSG00000162772	*ATF3*	TF_bZIP	7	10	8
828	ENSG00000167157	*PRRX2*	Homeobox	7	10	7
829	ENSG00000120798	*NR2C1*	RXR-like	7	10	7
830	ENSG00000128573	*FOXP2*	Fork_head	7	10	7
831	ENSG00000136936	*XPA*	Others	7	10	7
832	ENSG00000171425	*ZNF581*	zf-C2H2	7	10	7
833	ENSG00000151623	*NR3C2*	ESR-like	7	10	7
834	ENSG00000179943	*FIZ1*	zf-C2H2	7	10	7
835	ENSG00000204103	*MAFB*	TF_bZIP	7	10	7
836	ENSG00000204859	*ZBTB48*	ZBTB	7	10	7
837	ENSG00000249471	*ZNF324B*	zf-C2H2	7	10	7
838	ENSG00000083812	*ZNF324*	zf-C2H2	7	10	7
839	ENSG00000197951	*ZNF71*	zf-C2H2	7	10	7
840	ENSG00000204947	*ZNF425*	zf-C2H2	7	10	7
841	ENSG00000185730	*ZNF696*	zf-C2H2	7	10	7
842	ENSG00000175197	*DDIT3*	TF_bZIP	7	10	6
843	ENSG00000177374	*HIC1*	ZBTB	7	10	6

844	ENSG00000179588	*ZFPM1*	zf-C2H2	7	10	6
845	ENSG00000218891	*ZNF579*	zf-C2H2	7	10	6
846	ENSG00000130522	*JUND*	TF_bZIP	7	10	6
847	ENSG00000161277	*THAP8*	THAP	7	10	6
848	ENSG00000174652	*ZNF266*	zf-C2H2	7	10	6
849	ENSG00000196967	*ZNF585A*	zf-C2H2	7	10	6
850	ENSG00000186020	*ZNF529*	zf-C2H2	7	10	5
851	ENSG00000074219	*TEAD2*	TEA	7	10	5
852	ENSG00000170577	*SIX2*	Homeobox	7	10	5
853	ENSG00000151322	*NPAS3*	bHLH	7	10	4
854	ENSG00000167081	*PBX3*	Homeobox	7	11	10
855	ENSG00000196387	*ZNF140*	zf-C2H2	7	11	10
856	ENSG00000128709	*HOXD9*	Homeobox	7	11	9
857	ENSG00000137166	*FOXP4*	Fork_head	7	11	9
858	ENSG00000157613	*CREB3L1*	TF_bZIP	7	11	9
859	ENSG00000197279	*ZNF165*	zf-C2H2	7	11	9
860	ENSG00000159885	*ZNF222*	zf-C2H2	7	11	9
861	ENSG00000179361	*ARID3B*	ARID	7	11	8
862	ENSG00000187079	*TEAD1*	TEA	7	11	8
863	ENSG00000198393	*ZNF26*	zf-C2H2	7	11	8
864	ENSG00000105516	*DBP*	TF_bZIP	7	11	8
865	ENSG00000165821	*SALL2*	zf-C2H2	7	11	8
866	ENSG00000168286	*THAP11*	THAP	7	11	8
867	ENSG00000173039	*RELA*	RHD	7	11	8
868	ENSG00000204611	*ZNF616*	zf-C2H2	7	11	8
869	ENSG00000250571	*GLI4*	zf-C2H2	7	11	8
870	ENSG00000025434	*NR1H3*	THR-like	7	11	8
871	ENSG00000071564	*TCF3*	bHLH	7	11	8
872	ENSG00000152475	*ZNF837*	zf-C2H2	7	11	8
873	ENSG00000157429	*ZNF19*	zf-C2H2	7	11	8
874	ENSG00000249709	*ZNF564*	zf-C2H2	7	11	8
875	ENSG00000108312	*UBTF*	HMG	7	11	8
876	ENSG00000188629	*ZNF177*	zf-C2H2	7	11	8
877	ENSG00000198715	*GLMP*	NCU-G1	7	11	8
878	ENSG00000169635	*HIC2*	ZBTB	7	11	8
879	ENSG00000152454	*ZNF256*	zf-C2H2	7	11	7
880	ENSG00000160336	*ZNF761*	zf-C2H2	7	11	7
881	ENSG00000173275	*ZNF449*	zf-C2H2	7	11	7
882	ENSG00000185670	*ZBTB3*	ZBTB	7	11	7
883	ENSG00000122386	*ZNF205*	zf-C2H2	7	11	7
884	ENSG00000125520	*SLC2A4RG*	Others	7	11	7
885	ENSG00000196391	*ZNF774*	zf-C2H2	7	11	7
886	ENSG00000122515	*ZMIZ2*	zf-MIZ	7	11	7
887	ENSG00000179922	*ZNF784*	zf-C2H2	7	11	7
888	ENSG00000198816	*ZNF358*	zf-C2H2	7	11	7
889	ENSG00000172346	*CSDC2*	CSD	7	11	7
890	ENSG00000180318	*ALX1*	Homeobox	7	11	7
891	ENSG00000182983	*ZNF662*	zf-C2H2	7	11	7
892	ENSG00000166454	*ATMIN*	zf-C2H2	7	11	7
893	ENSG00000188868	*ZNF563*	zf-C2H2	7	11	6
894	ENSG00000113916	*BCL6*	ZBTB	7	11	6
895	ENSG00000130751	*NPAS1*	bHLH	7	11	6
896	ENSG00000136870	*ZNF189*	zf-C2H2	7	11	6
897	ENSG00000160113	*NR2F6*	RXR-like	7	11	6
898	ENSG00000166716	*ZNF592*	zf-C2H2	7	11	6
899	ENSG00000180806	*n_a*	Homeobox	7	11	6
900	ENSG00000229809	*ZNF688*	zf-C2H2	7	11	6
901	ENSG00000129071	*MBD4*	MBD	7	11	6
902	ENSG00000131408	*n_a*	THR-like	7	11	6
903	ENSG00000167394	*ZNF668*	zf-C2H2	7	11	6
904	ENSG00000187187	*ZNF546*	zf-C2H2	7	11	6
905	ENSG00000267680	*ZNF224*	zf-C2H2	7	11	6
906	ENSG00000020633	*RUNX3*	Runt	7	11	6
907	ENSG00000164920	*OSR2*	zf-C2H2	7	11	6
908	ENSG00000083828	*ZNF586*	zf-C2H2	7	11	6
909	ENSG00000188785	*ZNF548*	zf-C2H2	7	11	5
910	ENSG00000105698	*USF2*	bHLH	7	11	5
911	ENSG00000161642	*ZNF385A*	zf-C2H2	7	11	5
912	ENSG00000116990	*MYCL*	bHLH	7	11	5
913	ENSG00000196150	*ZNF250*	zf-C2H2	7	11	5
914	ENSG00000153048	*CARHSP1*	CSD	7	11	4
915	ENSG00000123405	*NFE2*	TF_bZIP	7	11	4
916	ENSG00000148143	*ZNF462*	zf-C2H2	7	12	9
917	ENSG00000242852	*ZNF709*	zf-C2H2	7	12	9
918	ENSG00000075426	*FOSL2*	TF_bZIP	7	12	8
919	ENSG00000110693	*SOX6*	HMG	7	12	8
920	ENSG00000170954	*ZNF415*	zf-C2H2	7	12	8
921	ENSG00000214022	*REPIN1*	zf-C2H2	7	12	8
922	ENSG00000089775	*ZBTB25*	ZBTB	7	12	7
923	ENSG00000101493	*ZNF516*	zf-C2H2	7	12	7
924	ENSG00000105708	*ZNF14*	zf-C2H2	7	12	7
925	ENSG00000157259	*GATAD1*	Others	7	12	7
926	ENSG00000164631	*ZNF12*	zf-C2H2	7	12	7
927	ENSG00000166704	*ZNF606*	zf-C2H2	7	12	7
928	ENSG00000181827	*RFX7*	RFX	7	12	7
929	ENSG00000251192	*ZNF674*	zf-C2H2	7	12	7
930	ENSG00000116604	*MEF2D*	SRF	7	12	7
931	ENSG00000182141	*ZNF708*	zf-C2H2	7	12	7
932	ENSG00000185219	*ZNF445*	zf-C2H2	7	12	7
933	ENSG00000197576	*HOXA4*	Homeobox	7	12	7
934	ENSG00000178951	*ZBTB7A*	ZBTB	7	12	7
935	ENSG00000197016	*ZNF470*	zf-C2H2	7	12	7
936	ENSG00000198353	*HOXC4*	Homeobox	7	12	7
937	ENSG00000117000	*RLF*	zf-C2H2	7	12	6

938	ENSG00000069667	*RORA*	THR-like	7	12	6
939	ENSG00000124831	*LRRFIP1*	LRRFIP	7	12	6
940	ENSG00000144026	*ZNF514*	zf-C2H2	7	12	6
941	ENSG00000164916	*FOXK1*	Fork_head	7	12	6
942	ENSG00000168795	*ZBTB5*	ZBTB	7	12	6
943	ENSG00000171827	*ZNF570*	zf-C2H2	7	12	6
944	ENSG00000172493	*AFF1*	AF-4	7	12	6
945	ENSG00000175387	*SMAD2*	MH1	7	12	6
946	ENSG00000189298	*ZKSCAN3*	zf-C2H2	7	12	6
947	ENSG00000278318	*ZNF229*	zf-C2H2	7	12	6
948	ENSG00000143614	*GATAD2B*	zf-GATA	7	12	6
949	ENSG00000198040	*ZNF84*	zf-C2H2	7	12	6
950	ENSG00000267508	*ZNF285*	zf-C2H2	7	12	6
951	ENSG00000260428	*SCX*	bHLH	7	12	6
952	ENSG00000187792	*ZNF70*	zf-C2H2	7	12	6
953	ENSG00000005889	*ZFX*	zf-C2H2	7	12	5
954	ENSG00000062370	*ZNF112*	zf-C2H2	7	12	5
955	ENSG00000084676	*NCOA1*	bHLH	7	12	5
956	ENSG00000142599	*RERE*	zf-GATA	7	12	5
957	ENSG00000170949	*ZNF160*	zf-C2H2	7	12	5
958	ENSG00000172379	*ARNT2*	bHLH	7	12	5
959	ENSG00000196498	*NCOR2*	MYB	7	12	5
960	ENSG00000198046	*ZNF667*	zf-C2H2	7	12	5
961	ENSG00000091656	*ZFHX4*	Homeobox	7	12	5
962	ENSG00000102984	*ZNF821*	zf-C2H2	7	12	5
963	ENSG00000114126	*TFDP2*	E2F	7	12	5
964	ENSG00000162702	*ZNF281*	zf-C2H2	7	12	5
965	ENSG00000184939	*ZFP90*	zf-C2H2	7	12	5
966	ENSG00000198093	*ZNF649*	zf-C2H2	7	12	5
967	ENSG00000198315	*ZKSCAN8*	zf-C2H2	7	12	5
968	ENSG00000074657	*ZNF532*	zf-C2H2	7	12	4
969	ENSG00000174282	*ZBTB4*	ZBTB	7	12	4
970	ENSG00000131668	*BARX1*	Homeobox	7	12	4
971	ENSG00000053254	*FOXN3*	Fork_head	7	12	3
972	ENSG00000184481	*FOXO4*	Fork_head	7	12	3
973	ENSG00000152217	*SETBP1*	Others	7	12	0
974	ENSG00000171448	*ZBTB26*	ZBTB	7	13	5
975	ENSG00000204644	*ZFP57*	zf-C2H2	8	7	10
976	ENSG00000160321	*ZNF208*	zf-C2H2	8	7	9
977	ENSG00000143458	*GABPB2*	Others	8	8	9
978	ENSG00000012223	*LTF*	Others	8	8	9
979	ENSG00000123358	*NR4A1*	NGFIB-like	8	8	7
980	ENSG00000181722	*ZBTB20*	ZBTB	8	9	10
981	ENSG00000173258	*ZNF483*	zf-C2H2	8	9	8
982	ENSG00000188033	*ZNF490*	zf-C2H2	8	9	8
983	ENSG00000274349	*ZNF658*	zf-C2H2	8	9	8
984	ENSG00000100219	*XBP1*	TF_bZIP	8	9	8
985	ENSG00000243660	*ZNF487*	Others	8	9	7
986	ENSG00000177842	*ZNF620*	zf-C2H2	8	9	6
987	ENSG00000196670	*ZFP62*	zf-C2H2	8	10	8
988	ENSG00000083817	*ZNF416*	zf-C2H2	8	10	8
989	ENSG00000131061	*ZNF341*	zf-C2H2	8	10	8
990	ENSG00000106004	*HOXA5*	Homeobox	8	10	8
991	ENSG00000198517	*MAFK*	TF_bZIP	8	10	8
992	ENSG00000162924	*REL*	RHD	8	10	7
993	ENSG00000124920	*MYRF*	NDT80/PhoG	8	10	7
994	ENSG00000198939	*ZFP2*	zf-C2H2	8	10	7
995	ENSG00000251247	*ZNF345*	zf-C2H2	8	10	7
996	ENSG00000197024	*ZNF398*	zf-C2H2	8	10	7
997	ENSG00000102554	*KLF5*	zf-C2H2	8	10	6
998	ENSG00000166526	*ZNF3*	zf-C2H2	8	10	6
999	ENSG00000172216	*CEBPB*	TF_bZIP	8	10	6
1000	ENSG00000180035	*ZNF48*	zf-C2H2	8	10	6
1001	ENSG00000189067	*LITAF*	zf-LITAF-like	8	10	5
1002	ENSG00000083814	*ZNF671*	zf-C2H2	8	10	5
1003	ENSG00000165804	*ZNF219*	zf-C2H2	8	10	5
1004	ENSG00000179456	*ZBTB18*	ZBTB	8	10	5
1005	ENSG00000198839	*ZNF277*	zf-C2H2	8	10	5
1006	ENSG00000159882	*ZNF230*	zf-C2H2	8	10	5
1007	ENSG00000182979	*MTA1*	zf-GATA	8	10	5
1008	ENSG00000184635	*ZNF93*	zf-C2H2	8	10	4
1009	ENSG00000160094	*ZNF362*	zf-C2H2	8	10	4
1010	ENSG00000155592	*ZKSCAN2*	zf-C2H2	8	11	9
1011	ENSG00000030419	*IKZF2*	zf-C2H2	8	11	9
1012	ENSG00000196597	*ZNF782*	zf-C2H2	8	11	8
1013	ENSG00000159917	*ZNF235*	zf-C2H2	8	11	8
1014	ENSG00000171223	*JUNB*	TF_bZIP	8	11	8
1015	ENSG00000172006	*ZNF554*	zf-C2H2	8	11	8
1016	ENSG00000196812	*ZSCAN16*	zf-C2H2	8	11	8
1017	ENSG00000197302	*n_a*	Others	8	11	8
1018	ENSG00000204920	*ZNF155*	zf-C2H2	8	11	8
1019	ENSG00000185513	*L3MBTL1*	zf-C2HC	8	11	8
1020	ENSG00000213928	*IRF9*	IRF	8	11	8
1021	ENSG00000141040	*ZNF287*	zf-C2H2	8	11	8
1022	ENSG00000189180	*ZNF33A*	zf-C2H2	8	11	7
1023	ENSG00000177606	*JUN*	TF_bZIP	8	11	7
1024	ENSG00000205189	*ZBTB10*	ZBTB	8	11	7
1025	ENSG00000130844	*ZNF331*	zf-C2H2	8	11	7
1026	ENSG00000165495	*PKNOX2*	Homeobox	8	11	7
1027	ENSG00000166529	*ZSCAN21*	zf-C2H2	8	11	7
1028	ENSG00000181007	*ZFP82*	zf-C2H2	8	11	7
1029	ENSG00000196378	*ZNF34*	zf-C2H2	8	11	7
1030	ENSG00000125618	*PAX8*	PAX	8	11	6
1031	ENSG00000124216	*SNAI1*	zf-C2H2	8	11	6

1032	ENSG00000147862	*NFIB*	CTF/NFI	8	11	6
1033	ENSG00000163909	*HEYL*	bHLH	8	11	6
1034	ENSG00000170265	*ZNF282*	zf-C2H2	8	11	6
1035	ENSG00000197363	*ZNF517*	zf-C2H2	8	11	6
1036	ENSG00000234284	*ZNF879*	zf-C2H2	8	11	6
1037	ENSG00000261221	*ZNF865*	zf-C2H2	8	11	6
1038	ENSG00000085644	*ZNF213*	zf-C2H2	8	11	6
1039	ENSG00000196724	*ZNF418*	zf-C2H2	8	11	6
1040	ENSG00000141568	*FOXK2*	Fork_head	8	11	6
1041	ENSG00000197162	*ZNF785*	zf-C2H2	8	11	6
1042	ENSG00000117318	*ID3*	bHLH	8	11	5
1043	ENSG00000166188	*ZNF319*	zf-C2H2	8	11	5
1044	ENSG00000114861	*FOXP1*	Fork_head	8	11	5
1045	ENSG00000160685	*ZBTB7B*	ZBTB	8	11	5
1046	ENSG00000177873	*ZNF619*	zf-C2H2	8	11	5
1047	ENSG00000101115	*SALL4*	zf-C2H2	8	11	5
1048	ENSG00000105229	*PIAS4*	zf-MIZ	8	11	5
1049	ENSG00000118707	*TGIF2*	Homeobox	8	11	4
1050	ENSG00000121413	*ZSCAN18*	zf-C2H2	8	11	4
1051	ENSG00000126561	*STAT5A*	STAT	8	11	4
1052	ENSG00000105419	*MEIS3*	Homeobox	8	11	4
1053	ENSG00000007866	*TEAD3*	TEA	8	11	3
1054	ENSG00000189164	*ZNF527*	zf-C2H2	8	11	3
1055	ENSG00000253293	*HOXA10*	Homeobox	8	11	3
1056	ENSG00000160062	*ZBTB8A*	ZBTB	8	12	9
1057	ENSG00000186026	*ZNF284*	zf-C2H2	8	12	9
1058	ENSG00000186448	*ZNF197*	zf-C2H2	8	12	9
1059	ENSG00000099326	*MZF1*	zf-C2H2	8	12	9
1060	ENSG00000215271	*HOMEZ*	Homeobox	8	12	8
1061	ENSG00000181666	*ZNF875*	zf-C2H2	8	12	8
1062	ENSG00000184517	*ZFP1*	zf-C2H2	8	12	8
1063	ENSG00000257923	*CUX1*	CUT	8	12	8
1064	ENSG00000068323	*TFE3*	bHLH	8	12	8
1065	ENSG00000102878	*HSF4*	HSF	8	12	8
1066	ENSG00000118689	*FOXO3*	Fork_head	8	12	7
1067	ENSG00000174586	*ZNF497*	zf-C2H2	8	12	7
1068	ENSG00000072310	*SREBF1*	bHLH	8	12	7
1069	ENSG00000136367	*ZFHX2*	Homeobox	8	12	7
1070	ENSG00000157514	*TSC22D3*	TSC22	8	12	7
1071	ENSG00000101216	*GMEB2*	SAND	8	12	7
1072	ENSG00000105722	*ERF*	ETS	8	12	7
1073	ENSG00000105880	*DLX5*	Homeobox	8	12	7
1074	ENSG00000119508	*NR4A3*	NGFIB-like	8	12	7
1075	ENSG00000143178	*TBX19*	T-box	8	12	7
1076	ENSG00000176371	*ZSCAN2*	zf-C2H2	8	12	7
1077	ENSG00000188283	*ZNF383*	zf-C2H2	8	12	7
1078	ENSG00000152926	*ZNF117*	zf-C2H2	8	12	6
1079	ENSG00000174428	*GTF2IRD2B*	GTF2I	8	12	6
1080	ENSG00000178665	*ZNF713*	zf-C2H2	8	12	6
1081	ENSG00000198182	*ZNF607*	zf-C2H2	8	12	6
1082	ENSG00000080298	*RFX3*	RFX	8	12	6
1083	ENSG00000105997	*HOXA3*	Homeobox	8	12	6
1084	ENSG00000116017	*ARID3A*	ARID	8	12	6
1085	ENSG00000143190	*POU2F1*	Pou	8	12	6
1086	ENSG00000171817	*ZNF540*	zf-C2H2	8	12	6
1087	ENSG00000176399	*DMRTA1*	DM	8	12	6
1088	ENSG00000186918	*ZNF395*	Others	8	12	6
1089	ENSG00000197566	*ZNF624*	zf-C2H2	8	12	6
1090	ENSG00000198453	*ZNF568*	zf-C2H2	8	12	6
1091	ENSG00000125846	*ZNF133*	zf-C2H2	8	12	6
1092	ENSG00000138378	*STAT4*	STAT	8	12	6
1093	ENSG00000143842	*SOX13*	HMG	8	12	6
1094	ENSG00000151090	*THRB*	THR-like	8	12	6
1095	ENSG00000159905	*n_a*	zf-C2H2	8	12	6
1096	ENSG00000163064	*EN1*	Homeobox	8	12	6
1097	ENSG00000165030	*NFIL3*	TF_bZIP	8	12	6
1098	ENSG00000170631	*ZNF16*	zf-C2H2	8	12	6
1099	ENSG00000185669	*n_a*	zf-C2H2	8	12	6
1100	ENSG00000187634	*SAMD11*	SAND	8	12	6
1101	ENSG00000196152	*ZNF79*	zf-C2H2	8	12	6
1102	ENSG00000196456	*ZNF775*	zf-C2H2	8	12	6
1103	ENSG00000137273	*FOXF2*	Fork_head	8	12	6
1104	ENSG00000159216	*RUNX1*	Runt	8	12	5
1105	ENSG00000167377	*ZNF23*	zf-C2H2	8	12	5
1106	ENSG00000168610	*STAT3*	STAT	8	12	5
1107	ENSG00000054598	*FOXC1*	Fork_head	8	12	5
1108	ENSG00000165512	*ZNF22*	zf-C2H2	8	12	5
1109	ENSG00000165655	*ZNF503*	Others	8	12	5
1110	ENSG00000182463	*TSHZ2*	zf-C2H2	8	12	5
1111	ENSG00000197863	*ZNF790*	zf-C2H2	8	12	5
1112	ENSG00000204231	*RXRB*	RXR-like	8	12	5
1113	ENSG00000005073	*HOXA11*	Homeobox	8	12	5
1114	ENSG00000061455	*PRDM6*	zf-C2H2	8	12	5
1115	ENSG00000117595	*IRF6*	IRF	8	12	5
1116	ENSG00000131759	*RARA*	THR-like	8	12	5
1117	ENSG00000150907	*FOXO1*	Fork_head	8	12	5
1118	ENSG00000196214	*ZNF766*	zf-C2H2	8	12	5
1119	ENSG00000197808	*ZNF461*	zf-C2H2	8	12	5
1120	ENSG00000198911	*SREBF2*	bHLH	8	12	5
1121	ENSG00000236104	*ZBTB22*	ZBTB	8	12	5
1122	ENSG00000198846	*TOX*	HMG	8	12	5
1123	ENSG00000150347	*ARID5B*	ARID	8	12	4
1124	ENSG00000196867	*ZFP28*	zf-C2H2	8	12	4
1125	ENSG00000142065	*ZFP14*	zf-C2H2	8	12	4

1126	ENSG00000143379	*SETDB1*	MBD	8	12	4
1127	ENSG00000143437	*ARNT*	bHLH	8	12	4
1128	ENSG00000146592	*CREB5*	TF_bZIP	8	12	4
1129	ENSG00000175727	*MLXIP*	bHLH	8	12	4
1130	ENSG00000196275	*GTF2IRD2*	GTF2I	8	12	4
1131	ENSG00000057935	*MTA3*	zf-GATA	8	12	4
1132	ENSG00000089486	*CDIP1*	zf-LITAF-like	8	12	4
1133	ENSG00000100968	*NFATC4*	RHD	8	12	4
1134	ENSG00000112658	*SRF*	SRF	8	12	4
1135	ENSG00000115738	*ID2*	bHLH	8	12	4
1136	ENSG00000150051	*MKX*	Homeobox	8	12	4
1137	ENSG00000170581	*STAT2*	STAT	8	12	4
1138	ENSG00000184271	*n_a*	Pou	8	12	4
1139	ENSG00000196605	*ZNF846*	zf-C2H2	8	12	4
1140	ENSG00000251493	*FOXD1*	Fork_head	8	12	4
1141	ENSG00000167771	*RCOR2*	MYB	8	12	4
1142	ENSG00000130684	*ZNF337*	zf-C2H2	8	12	3
1143	ENSG00000131196	*NFATC1*	RHD	8	12	3
1144	ENSG00000144218	*AFF3*	AF-4	8	12	3
1145	ENSG00000148737	*TCF7L2*	HMG	8	12	3
1146	ENSG00000164093	*PITX2*	Homeobox	8	12	3
1147	ENSG00000010030	*ETV7*	ETS	8	12	3
1148	ENSG00000102349	*KLF8*	zf-C2H2	8	12	3
1149	ENSG00000140044	*JDP2*	TF_bZIP	8	12	3
1150	ENSG00000143995	*MEIS1*	Homeobox	8	12	3
1151	ENSG00000196843	*ARID5A*	ARID	8	12	3
1152	ENSG00000205903	*ZNF316*	zf-C2H2	8	12	3
1153	ENSG00000221869	*CEBPD*	TF_bZIP	8	12	3
1154	ENSG00000112182	*BACH2*	TF_bZIP	8	12	2
1155	ENSG00000114853	*ZBTB47*	ZBTB	8	12	2
1156	ENSG00000119950	*MXI1*	bHLH	8	12	2
1157	ENSG00000126351	*THRA*	THR-like	8	12	2
1158	ENSG00000173757	*STAT5B*	STAT	8	12	2
1159	ENSG00000196323	*ZBTB44*	ZBTB	8	12	2
1160	ENSG00000126603	*GLIS2*	zf-C2H2	8	12	2
1161	ENSG00000126778	*SIX1*	Homeobox	8	12	2
1162	ENSG00000164684	*ZNF704*	Others	8	12	2
1163	ENSG00000170370	*EMX2*	Homeobox	8	12	2
1164	ENSG00000178573	*MAF*	TF_bZIP	8	12	2
1165	ENSG00000214717	*ZBED1*	zf-BED	8	12	2
1166	ENSG00000120837	*NFYB*	NF-YB	8	12	1
1167	ENSG00000139083	*ETV6*	ETS	8	12	1
1168	ENSG00000153814	*JAZF1*	zf-C2H2	8	12	1
1169	ENSG00000172201	*ID4*	bHLH	8	12	1
1170	ENSG00000123095	*BHLHE41*	bHLH	8	12	0
1171	ENSG00000124766	*SOX4*	HMG	8	12	0
1172	ENSG00000187607	*ZNF286A*	zf-C2H2	8	13	8
1173	ENSG00000168214	*RBPJ*	CSL	9	7	8
1174	ENSG00000175691	*ZNF77*	zf-C2H2	9	8	9
1175	ENSG00000170166	*HOXD4*	Homeobox	9	9	10
1176	ENSG00000121075	*TBX4*	T-box	9	9	8
1177	ENSG00000169981	*ZNF35*	zf-C2H2	9	10	10
1178	ENSG00000204946	*ZNF783*	zf-C2H2	9	10	10
1179	ENSG00000137834	*SMAD6*	MH1	9	10	9
1180	ENSG00000284691	*n_a*	zf-C2H2	9	10	8
1181	ENSG00000122877	*EGR2*	zf-C2H2	9	10	8
1182	ENSG00000143355	*LHX9*	Homeobox	9	10	7
1183	ENSG00000161914	*ZNF653*	zf-C2H2	9	11	6
1184	ENSG00000006704	*GTF2IRD1*	GTF2I	9	11	5
1185	ENSG00000179627	*n_a*	ZBTB	9	12	8
1186	ENSG00000103199	*ZNF500*	zf-C2H2	9	12	6
1187	ENSG00000106031	*HOXA13*	Homeobox	9	12	5
1188	ENSG00000176842	*IRX5*	Homeobox	9	12	5
1189	ENSG00000186350	*RXRA*	RXR-like	9	12	5
1190	ENSG00000152284	*TCF7L1*	HMG	9	12	3
1191	ENSG00000186496	*ZNF396*	zf-C2H2	9	13	7
