# Supplementary material for: Decoding the differentiation of mesenchymal stem cells into mesangial cells at the transcriptomic level
Source: BMC Genomics. 2020 Jul 7;21:467. doi: 10.1186/s12864-020-06868-5 (PMC7339572; doi:10.1186/s12864-020-06868-5)
Supplement: Supplementary file 4 — Additional file 3. Monotonic descending pattern genes with DE ≤ 4. [file 12864_2020_6868_MOESM3_ESM.pdf]

Monotonic descending pattern genes with DE≤4

	Ensembl Gene ID	Gene Names	DE	p.value	q.value	SVDE
1	ENSG00000100994	*PYGB*	0	0	0	0
2	ENSG00000144136	*SLC20A1*	0	0	0	0
3	ENSG00000168288	*MMADHC*	0	0	0	0
4	ENSG00000184661	*CDCA2*	0	0	0	0
5	ENSG00000198805	*PNP*	0	0	0	0
6	ENSG00000143549	*TPM3*	0	0	0	0.01
7	ENSG00000171552	*BCL2L1*	0	0	0	0.06
8	ENSG00000110955	*ATP5F1B*	0	0	0	0.08
9	ENSG00000100462	*PRMT5*	0	0	0	0.14
10	ENSG00000135046	*ANXA1*	0	0	0	0.15
11	ENSG00000115053	*NCL*	0	0	0	0.16
12	ENSG00000148773	*MKI67*	0	0	0	0.19
13	ENSG00000178773	*CPNE7*	0	0	0	0.29
14	ENSG00000188064	*WNT7B*	0	0	0	0.31
15	ENSG00000150753	*CCT5*	0	0	0	0.4
16	ENSG00000175592	*FOSL1*	0	0	0	0.4
17	ENSG00000181163	*NPM1*	0	0	0	0.4
18	ENSG00000140525	*FANCI*	0	0	0	0.48
19	ENSG00000102312	*PORCN*	0	0	0	0.56
20	ENSG00000123473	*STIL*	0	0	0	0.61
21	ENSG00000175166	*PSMD2*	0	0	0	0.74
22	ENSG00000165678	*GHITM*	0	0	0	0.79
23	ENSG00000139289	*PHLDA1*	0	0	0	0.8
24	ENSG00000150630	*VEGFC*	0	0	0	2.46
25	ENSG00000100034	*PPM1F*	1	6.38E-07	8.70E-05	0
26	ENSG00000137309	*HMGA1*	1	6.38E-07	8.70E-05	0
27	ENSG00000159131	*GART*	1	6.38E-07	8.70E-05	0
28	ENSG00000203760	*CENPW*	1	6.38E-07	8.70E-05	0.03
29	ENSG00000198901	*PRC1*	1	6.38E-07	8.70E-05	0.07
30	ENSG00000124191	*TOX2*	1	6.38E-07	8.70E-05	0.08
31	ENSG00000197467	*COL13A1*	1	6.38E-07	8.70E-05	0.08
32	ENSG00000071539	*TRIP13*	1	6.38E-07	8.70E-05	0.09
33	ENSG00000089685	*BIRC5*	1	6.38E-07	8.70E-05	0.09
34	ENSG00000118785	*SPP1*	1	6.38E-07	8.70E-05	0.09
35	ENSG00000111206	*FOXM1*	1	6.38E-07	8.70E-05	0.11
36	ENSG00000119714	*GPR68*	1	6.38E-07	8.70E-05	0.11
37	ENSG00000166803	*PCLAF*	1	6.38E-07	8.70E-05	0.11
38	ENSG00000135472	*FAIM2*	1	6.38E-07	8.70E-05	0.12
39	ENSG00000196878	*LAMB3*	1	6.38E-07	8.70E-05	0.12
40	ENSG00000100739	*BDKRB1*	1	6.38E-07	8.70E-05	0.13
41	ENSG00000110092	*CCND1*	1	6.38E-07	8.70E-05	0.13
42	ENSG00000113368	*LMNB1*	1	6.38E-07	8.70E-05	0.15
43	ENSG00000196497	*IPO4*	1	6.38E-07	8.70E-05	0.16
44	ENSG00000088325	*TPX2*	1	6.38E-07	8.70E-05	0.17
45	ENSG00000184232	*OAF*	1	6.38E-07	8.70E-05	0.17
46	ENSG00000258947	*TUBB3*	1	6.38E-07	8.70E-05	0.18
47	ENSG00000198826	*ARHGAP11A*	1	6.38E-07	8.70E-05	0.21
48	ENSG00000133195	*SLC39A11*	1	6.38E-07	8.70E-05	0.22
49	ENSG00000183856	*IQGAP3*	1	6.38E-07	8.70E-05	0.22
50	ENSG00000107984	*DKK1*	1	6.38E-07	8.70E-05	0.25
51	ENSG00000165733	*BMS1*	1	6.38E-07	8.70E-05	0.25
52	ENSG00000171241	*SHCBP1*	1	6.38E-07	8.70E-05	0.26
53	ENSG00000137812	*KNL1*	1	6.38E-07	8.70E-05	0.27
54	ENSG00000125912	*NCLN*	1	6.38E-07	8.70E-05	0.29
55	ENSG00000132780	*NASP*	1	6.38E-07	8.70E-05	0.29
56	ENSG00000119392	*GLE1*	1	6.38E-07	8.70E-05	0.31
57	ENSG00000109917	*ZPR1*	1	6.38E-07	8.70E-05	0.32
58	ENSG00000186185	*KIF18B*	1	6.38E-07	8.70E-05	0.32
59	ENSG00000214114	*MYCBP*	1	6.38E-07	8.70E-05	0.33
60	ENSG00000138162	*TACC2*	1	6.38E-07	8.70E-05	0.34
61	ENSG00000143228	*NUF2*	1	6.38E-07	8.70E-05	0.35
62	ENSG00000187522	*HSPA14*	1	6.38E-07	8.70E-05	0.35
63	ENSG00000100526	*CDKN3*	1	6.38E-07	8.70E-05	0.38
64	ENSG00000068438	*FTSJ1*	1	6.38E-07	8.70E-05	0.4
65	ENSG00000131467	*PSME3*	1	6.38E-07	8.70E-05	0.4
66	ENSG00000109511	*ANXA10*	1	6.38E-07	8.70E-05	0.41
67	ENSG00000138279	*ANXA7*	1	6.38E-07	8.70E-05	0.41
68	ENSG00000090889	*KIF4A*	1	6.38E-07	8.70E-05	0.42
69	ENSG00000104824	*HNRNPL*	1	6.38E-07	8.70E-05	0.42
70	ENSG00000142945	*KIF2C*	1	6.38E-07	8.70E-05	0.46
71	ENSG00000144381	*HSPD1*	1	6.38E-07	8.70E-05	0.46
72	ENSG00000122966	*CIT*	1	6.38E-07	8.70E-05	0.47
73	ENSG00000149554	*CHEK1*	1	6.38E-07	8.70E-05	0.47
74	ENSG00000101447	*FAM83D*	1	6.38E-07	8.70E-05	0.48
75	ENSG00000089159	*PXN*	1	6.38E-07	8.70E-05	0.49
76	ENSG00000085063	*CD59*	1	6.38E-07	8.70E-05	0.5
77	ENSG00000144063	*MALL*	1	6.38E-07	8.70E-05	0.5
78	ENSG00000008394	*MGST1*	1	6.38E-07	8.70E-05	0.51
79	ENSG00000271303	*SRXN1*	1	6.38E-07	8.70E-05	0.51
80	ENSG00000026508	*CD44*	1	6.38E-07	8.70E-05	0.52
81	ENSG00000114738	*MAPKAPK3*	1	6.38E-07	8.70E-05	0.53
82	ENSG00000055044	*NOP58*	1	6.38E-07	8.70E-05	0.55
83	ENSG00000092853	*CLSPN*	1	6.38E-07	8.70E-05	0.55
84	ENSG00000099783	*HNRNPM*	1	6.38E-07	8.70E-05	0.55
85	ENSG00000106089	*STX1A*	1	6.38E-07	8.70E-05	0.6
86	ENSG00000131747	*TOP2A*	1	6.38E-07	8.70E-05	0.63
87	ENSG00000175426	*PCSK1*	1	6.38E-07	8.70E-05	0.63
88	ENSG00000181751	*C5orf30*	1	6.38E-07	8.70E-05	0.63
89	ENSG00000132436	*FIGNL1*	1	6.38E-07	8.70E-05	0.64
90	ENSG00000065328	*MCM10*	1	6.38E-07	8.70E-05	0.65
91	ENSG00000072571	*HMMR*	1	6.38E-07	8.70E-05	0.65

92	ENSG00000146410	*MTFR2*	1	6.38E-07	8.70E-05	0.65
93	ENSG00000181019	*NQO1*	1	6.38E-07	8.70E-05	0.66
94	ENSG00000198768	*APCDD1L*	1	6.38E-07	8.70E-05	0.66
95	ENSG00000163468	*CCT3*	1	6.38E-07	8.70E-05	0.73
96	ENSG00000166881	*NEMP1*	1	6.38E-07	8.70E-05	0.74
97	ENSG00000093009	*CDC45*	1	6.38E-07	8.70E-05	0.8
98	ENSG00000143127	*ITGA10*	1	6.38E-07	8.70E-05	0.82
99	ENSG00000109805	*NCAPG*	1	6.38E-07	8.70E-05	0.98
100	ENSG00000153044	*CENPH*	1	6.38E-07	8.70E-05	0.98
101	ENSG00000024526	*DEPDC1*	1	6.38E-07	8.70E-05	1.01
102	ENSG00000164038	*SLC9B2*	1	6.38E-07	8.70E-05	1.03
103	ENSG00000135476	*ESPL1*	1	6.38E-07	8.70E-05	1.06
104	ENSG00000103522	*IL21R*	1	6.38E-07	8.70E-05	1.07
105	ENSG00000277161	*PIGW*	1	6.38E-07	8.70E-05	1.07
106	ENSG00000013016	*EHD3*	1	6.38E-07	8.70E-05	1.09
107	ENSG00000102172	*SMS*	1	6.38E-07	8.70E-05	1.09
108	ENSG00000164032	*H2AFZ*	1	6.38E-07	8.70E-05	1.18
109	ENSG00000087586	*AURKA*	1	6.38E-07	8.70E-05	1.41
110	ENSG00000119777	*TMEM214*	1	6.38E-07	8.70E-05	1.45
111	ENSG00000122952	*ZWINT*	1	6.38E-07	8.70E-05	1.61
112	ENSG00000176890	*TYMS*	1	6.38E-07	8.70E-05	1.97
113	ENSG00000158402	*CDC25C*	1	6.38E-07	8.70E-05	2.14
114	ENSG00000108671	*PSMD11*	1	6.38E-07	8.70E-05	3.15
115	ENSG00000013297	*CLDN11*	2	7.66E-06	0.000364742	0
116	ENSG00000107959	*PITRM1*	2	7.66E-06	0.000364742	0
117	ENSG00000110090	*CPT1A*	2	7.66E-06	0.000364742	0
118	ENSG00000113643	*RARS*	2	7.66E-06	0.000364742	0
119	ENSG00000163584	*RPL22L1*	2	7.66E-06	0.000364742	0
120	ENSG00000121579	*NAA50*	2	7.66E-06	0.000364742	0.01
121	ENSG00000143387	*CTSK*	2	7.66E-06	0.000364742	0.01
122	ENSG00000172115	*CYCS*	2	7.66E-06	0.000364742	0.02
123	ENSG00000113013	*HSPA9*	2	7.66E-06	0.000364742	0.03
124	ENSG00000125968	*ID1*	2	7.66E-06	0.000364742	0.03
125	ENSG00000008083	*JARID2*	2	7.66E-06	0.000364742	0.04
126	ENSG00000033011	*ALG1*	2	7.66E-06	0.000364742	0.05
127	ENSG00000171488	*LRRC8C*	2	7.66E-06	0.000364742	0.06
128	ENSG00000256235	*SMIM3*	2	7.66E-06	0.000364742	0.06
129	ENSG00000011426	*ANLN*	2	7.66E-06	0.000364742	0.07
130	ENSG00000057608	*GDI2*	2	7.66E-06	0.000364742	0.07
131	ENSG00000138134	*STAMBPL1*	2	7.66E-06	0.000364742	0.07
132	ENSG00000152234	*ATP5F1A*	2	7.66E-06	0.000364742	0.07
133	ENSG00000101367	*MAPRE1*	2	7.66E-06	0.000364742	0.09
134	ENSG00000119139	*TJP2*	2	7.66E-06	0.000364742	0.09
135	ENSG00000148680	*HTR7*	2	7.66E-06	0.000364742	0.09
136	ENSG00000172009	*THOP1*	2	7.66E-06	0.000364742	0.09
137	ENSG00000111667	*USP5*	2	7.66E-06	0.000364742	0.1
138	ENSG00000107937	*GTPBP4*	2	7.66E-06	0.000364742	0.11
139	ENSG00000110330	*BIRC2*	2	7.66E-06	0.000364742	0.12
140	ENSG00000115884	*SDC1*	2	7.66E-06	0.000364742	0.12
141	ENSG00000117724	*CENPF*	2	7.66E-06	0.000364742	0.12
142	ENSG00000075218	*GTSE1*	2	7.66E-06	0.000364742	0.13
143	ENSG00000152253	*SPC25*	2	7.66E-06	0.000364742	0.13
144	ENSG00000058729	*RIOK2*	2	7.66E-06	0.000364742	0.14
145	ENSG00000120948	*TARDBP*	2	7.66E-06	0.000364742	0.14
146	ENSG00000170522	*ELOVL6*	2	7.66E-06	0.000364742	0.14
147	ENSG00000165244	*ZNF367*	2	7.66E-06	0.000364742	0.15
148	ENSG00000244405	*ETV5*	2	7.66E-06	0.000364742	0.15
149	ENSG00000142634	*EFHD2*	2	7.66E-06	0.000364742	0.16
150	ENSG00000173418	*NAA20*	2	7.66E-06	0.000364742	0.16
151	ENSG00000186193	*SAPCD2*	2	7.66E-06	0.000364742	0.16
152	ENSG00000104738	*MCM4*	2	7.66E-06	0.000364742	0.17
153	ENSG00000138376	*BARD1*	2	7.66E-06	0.000364742	0.17
154	ENSG00000147536	*GINS4*	2	7.66E-06	0.000364742	0.17
155	ENSG00000115233	*PSMD14*	2	7.66E-06	0.000364742	0.18
156	ENSG00000137310	*TCF19*	2	7.66E-06	0.000364742	0.19
157	ENSG00000138182	*KIF20B*	2	7.66E-06	0.000364742	0.19
158	ENSG00000139618	*BRCA2*	2	7.66E-06	0.000364742	0.19
159	ENSG00000168078	*PBK*	2	7.66E-06	0.000364742	0.2
160	ENSG00000071054	*MAP4K4*	2	7.66E-06	0.000364742	0.21
161	ENSG00000109919	*MTCH2*	2	7.66E-06	0.000364742	0.21
162	ENSG00000102359	*SRPX2*	2	7.66E-06	0.000364742	0.22
163	ENSG00000119969	*HELLS*	2	7.66E-06	0.000364742	0.22
164	ENSG00000184254	*ALDH1A3*	2	7.66E-06	0.000364742	0.22
165	ENSG00000135451	*TROAP*	2	7.66E-06	0.000364742	0.23
166	ENSG00000166451	*CENPN*	2	7.66E-06	0.000364742	0.23
167	ENSG00000167900	*TK1*	2	7.66E-06	0.000364742	0.23
168	ENSG00000100297	*MCM5*	2	7.66E-06	0.000364742	0.25
169	ENSG00000123485	*HJURP*	2	7.66E-06	0.000364742	0.26
170	ENSG00000125871	*MGME1*	2	7.66E-06	0.000364742	0.26
171	ENSG00000183715	*OPCML*	2	7.66E-06	0.000364742	0.26
172	ENSG00000165480	*SKA3*	2	7.66E-06	0.000364742	0.27
173	ENSG00000213551	*DNAJC9*	2	7.66E-06	0.000364742	0.27
174	ENSG00000122566	*HNRNPA2B1*	2	7.66E-06	0.000364742	0.28
175	ENSG00000135486	*HNRNPA1*	2	7.66E-06	0.000364742	0.29
176	ENSG00000137364	*TPMT*	2	7.66E-06	0.000364742	0.29
177	ENSG00000169679	*BUB1*	2	7.66E-06	0.000364742	0.29
178	ENSG00000104368	*PLAT*	2	7.66E-06	0.000364742	0.3
179	ENSG00000144959	*NCEH1*	2	7.66E-06	0.000364742	0.3
180	ENSG00000151725	*CENPU*	2	7.66E-06	0.000364742	0.3
181	ENSG00000154839	*SKA1*	2	7.66E-06	0.000364742	0.3
182	ENSG00000169436	*COL22A1*	2	7.66E-06	0.000364742	0.3
183	ENSG00000189001	*SBSN*	2	7.66E-06	0.000364742	0.3
184	ENSG00000138018	*SELENOI*	2	7.66E-06	0.000364742	0.31
185	ENSG00000164985	*PSIP1*	2	7.66E-06	0.000364742	0.31

186	ENSG00000116120	*FARSB*	2	7.66E-06	0.000364742	0.32
187	ENSG00000137807	*KIF23*	2	7.66E-06	0.000364742	0.32
188	ENSG00000165490	*DDIAS*	2	7.66E-06	0.000364742	0.32
189	ENSG00000104691	*UBXN8*	2	7.66E-06	0.000364742	0.33
190	ENSG00000196460	*RFX8*	2	7.66E-06	0.000364742	0.33
191	ENSG00000162746	*FCRLB*	2	7.66E-06	0.000364742	0.34
192	ENSG00000171723	*GPHN*	2	7.66E-06	0.000364742	0.34
193	ENSG00000173085	*COQ2*	2	7.66E-06	0.000364742	0.34
194	ENSG00000174442	*ZWILCH*	2	7.66E-06	0.000364742	0.34
195	ENSG00000117399	*CDC20*	2	7.66E-06	0.000364742	0.35
196	ENSG00000169258	*GPRIN1*	2	7.66E-06	0.000364742	0.35
197	ENSG00000186871	*ERCC6L*	2	7.66E-06	0.000364742	0.35
198	ENSG00000089006	*SNX5*	2	7.66E-06	0.000364742	0.36
199	ENSG00000100065	*CARD10*	2	7.66E-06	0.000364742	0.36
200	ENSG00000115840	*SLC25A12*	2	7.66E-06	0.000364742	0.36
201	ENSG00000136108	*CKAP2*	2	7.66E-06	0.000364742	0.36
202	ENSG00000154027	*AK5*	2	7.66E-06	0.000364742	0.36
203	ENSG00000167670	*CHAF1A*	2	7.66E-06	0.000364742	0.36
204	ENSG00000168917	*SLC35G2*	2	7.66E-06	0.000364742	0.36
205	ENSG00000237649	*KIFC1*	2	7.66E-06	0.000364742	0.36
206	ENSG00000073150	*PANX2*	2	7.66E-06	0.000364742	0.37
207	ENSG00000084090	*STARD7*	2	7.66E-06	0.000364742	0.37
208	ENSG00000092199	*HNRNPC*	2	7.66E-06	0.000364742	0.37
209	ENSG00000114251	*WNT5A*	2	7.66E-06	0.000364742	0.37
210	ENSG00000136045	*PWP1*	2	7.66E-06	0.000364742	0.37
211	ENSG00000166825	*ANPEP*	2	7.66E-06	0.000364742	0.37
212	ENSG00000167325	*RRM1*	2	7.66E-06	0.000364742	0.37
213	ENSG00000176692	*FOXC2*	2	7.66E-06	0.000364742	0.37
214	ENSG00000137821	*LRRC49*	2	7.66E-06	0.000364742	0.38
215	ENSG00000139318	*DUSP6*	2	7.66E-06	0.000364742	0.38
216	ENSG00000171848	*RRM2*	2	7.66E-06	0.000364742	0.38
217	ENSG00000129195	*PIMREG*	2	7.66E-06	0.000364742	0.4
218	ENSG00000076382	*SPAG5*	2	7.66E-06	0.000364742	0.41
219	ENSG00000101266	*CSNK2A1*	2	7.66E-06	0.000364742	0.41
220	ENSG00000104341	*LAPTM4B*	2	7.66E-06	0.000364742	0.41
221	ENSG00000105825	*TFPI2*	2	7.66E-06	0.000364742	0.41
222	ENSG00000174371	*EXO1*	2	7.66E-06	0.000364742	0.41
223	ENSG00000108010	*GLRX3*	2	7.66E-06	0.000364742	0.42
224	ENSG00000129055	*ANAPC13*	2	7.66E-06	0.000364742	0.43
225	ENSG00000137804	*NUSAP1*	2	7.66E-06	0.000364742	0.43
226	ENSG00000170606	*HSPA4*	2	7.66E-06	0.000364742	0.43
227	ENSG00000058866	*DGKG*	2	7.66E-06	0.000364742	0.44
228	ENSG00000117650	*NEK2*	2	7.66E-06	0.000364742	0.44
229	ENSG00000148672	*GLUD1*	2	7.66E-06	0.000364742	0.44
230	ENSG00000165280	*VCP*	2	7.66E-06	0.000364742	0.44
231	ENSG00000051341	*POLQ*	2	7.66E-06	0.000364742	0.45
232	ENSG00000134308	*YWHAQ*	2	7.66E-06	0.000364742	0.45
233	ENSG00000156970	*BUB1B*	2	7.66E-06	0.000364742	0.45
234	ENSG00000167553	*TUBA1C*	2	7.66E-06	0.000364742	0.45
235	ENSG00000103018	*CYB5B*	2	7.66E-06	0.000364742	0.46
236	ENSG00000115363	*EVA1A*	2	7.66E-06	0.000364742	0.46
237	ENSG00000164109	*MAD2L1*	2	7.66E-06	0.000364742	0.46
238	ENSG00000187678	*SPRY4*	2	7.66E-06	0.000364742	0.46
239	ENSG00000040275	*SPDL1*	2	7.66E-06	0.000364742	0.47
240	ENSG00000160957	*RECQL4*	2	7.66E-06	0.000364742	0.47
241	ENSG00000128567	*PODXL*	2	7.66E-06	0.000364742	0.48
242	ENSG00000166851	*PLK1*	2	7.66E-06	0.000364742	0.48
243	ENSG00000105011	*ASF1B*	2	7.66E-06	0.000364742	0.49
244	ENSG00000157456	*CCNB2*	2	7.66E-06	0.000364742	0.49
245	ENSG00000170312	*CDK1*	2	7.66E-06	0.000364742	0.49
246	ENSG00000276043	*UHRF1*	2	7.66E-06	0.000364742	0.5
247	ENSG00000134001	*EIF2S1*	2	7.66E-06	0.000364742	0.51
248	ENSG00000184178	*SCFD2*	2	7.66E-06	0.000364742	0.51
249	ENSG00000081059	*TCF7*	2	7.66E-06	0.000364742	0.52
250	ENSG00000147010	*SH3KBP1*	2	7.66E-06	0.000364742	0.52
251	ENSG00000165304	*MELK*	2	7.66E-06	0.000364742	0.52
252	ENSG00000111247	*RAD51AP1*	2	7.66E-06	0.000364742	0.53
253	ENSG00000171208	*NETO2*	2	7.66E-06	0.000364742	0.54
254	ENSG00000066279	*ASPM*	2	7.66E-06	0.000364742	0.56
255	ENSG00000170779	*CDCA4*	2	7.66E-06	0.000364742	0.56
256	ENSG00000173692	*PSMD1*	2	7.66E-06	0.000364742	0.56
257	ENSG00000106853	*PTGR1*	2	7.66E-06	0.000364742	0.57
258	ENSG00000139842	*CUL4A*	2	7.66E-06	0.000364742	0.57
259	ENSG00000168496	*FEN1*	2	7.66E-06	0.000364742	0.57
260	ENSG00000169607	*CKAP2L*	2	7.66E-06	0.000364742	0.57
261	ENSG00000182871	*COL18A1*	2	7.66E-06	0.000364742	0.57
262	ENSG00000136122	*BORA*	2	7.66E-06	0.000364742	0.58
263	ENSG00000144583	*MARCHF4*	2	7.66E-06	0.000364742	0.58
264	ENSG00000101003	*GINS1*	2	7.66E-06	0.000364742	0.59
265	ENSG00000134057	*CCNB1*	2	7.66E-06	0.000364742	0.59
266	ENSG00000029993	*HMGB3*	2	7.66E-06	0.000364742	0.6
267	ENSG00000115648	*MLPH*	2	7.66E-06	0.000364742	0.6
268	ENSG00000138160	*KIF11*	2	7.66E-06	0.000364742	0.6
269	ENSG00000175063	*UBE2C*	2	7.66E-06	0.000364742	0.6
270	ENSG00000112984	*KIF20A*	2	7.66E-06	0.000364742	0.61
271	ENSG00000154127	*UBASH3B*	2	7.66E-06	0.000364742	0.61
272	ENSG00000169908	*TM4SF1*	2	7.66E-06	0.000364742	0.61
273	ENSG00000013810	*TACC3*	2	7.66E-06	0.000364742	0.64
274	ENSG00000165891	*E2F7*	2	7.66E-06	0.000364742	0.64
275	ENSG00000118193	*KIF14*	2	7.66E-06	0.000364742	0.65
276	ENSG00000148843	*PDCD11*	2	7.66E-06	0.000364742	0.7
277	ENSG00000163938	*GNL3*	2	7.66E-06	0.000364742	0.7
278	ENSG00000073111	*MCM2*	2	7.66E-06	0.000364742	0.71
279	ENSG00000130038	*CRACR2A*	2	7.66E-06	0.000364742	0.71

280	ENSG00000121211	*MND1*	2	7.66E-06	0.000364742	0.72
281	ENSG00000101057	*MYBL2*	2	7.66E-06	0.000364742	0.73
282	ENSG00000104365	*IKBKB*	2	7.66E-06	0.000364742	0.73
283	ENSG00000161800	*RACGAP1*	2	7.66E-06	0.000364742	0.74
284	ENSG00000108829	*LRRC59*	2	7.66E-06	0.000364742	0.76
285	ENSG00000079246	*XRCC5*	2	7.66E-06	0.000364742	0.77
286	ENSG00000128944	*KNSTRN*	2	7.66E-06	0.000364742	0.77
287	ENSG00000054356	*PTPRN*	2	7.66E-06	0.000364742	0.8
288	ENSG00000111665	*CDCA3*	2	7.66E-06	0.000364742	0.8
289	ENSG00000135316	*SYNCRIP*	2	7.66E-06	0.000364742	0.8
290	ENSG00000182481	*KPNA2*	2	7.66E-06	0.000364742	0.8
291	ENSG00000156261	*CCT8*	2	7.66E-06	0.000364742	0.86
292	ENSG00000184445	*KNTC1*	2	7.66E-06	0.000364742	0.86
293	ENSG00000261652	*C15orf65*	2	7.66E-06	0.000364742	0.86
294	ENSG00000100280	*AP1B1*	2	7.66E-06	0.000364742	0.92
295	ENSG00000125166	*GOT2*	2	7.66E-06	0.000364742	0.94
296	ENSG00000228716	*DHFR*	2	7.66E-06	0.000364742	0.94
297	ENSG00000126787	*DLGAP5*	2	7.66E-06	0.000364742	0.99
298	ENSG00000115687	*PASK*	2	7.66E-06	0.000364742	1
299	ENSG00000101412	*E2F1*	2	7.66E-06	0.000364742	1.02
300	ENSG00000102572	*STK24*	2	7.66E-06	0.000364742	1.02
301	ENSG00000115325	*DOK1*	2	7.66E-06	0.000364742	1.06
302	ENSG00000225968	*ELFN1*	2	7.66E-06	0.000364742	1.08
303	ENSG00000128973	*CLN6*	2	7.66E-06	0.000364742	1.09
304	ENSG00000163507	*CIP2A*	2	7.66E-06	0.000364742	1.09
305	ENSG00000204899	*MZT1*	2	7.66E-06	0.000364742	1.13
306	ENSG00000123975	*CKS2*	2	7.66E-06	0.000364742	1.15
307	ENSG00000154920	*EME1*	2	7.66E-06	0.000364742	1.21
308	ENSG00000130669	*PAK4*	2	7.66E-06	0.000364742	1.23
309	ENSG00000163535	*SGO2*	2	7.66E-06	0.000364742	1.27
310	ENSG00000067533	*RRP15*	2	7.66E-06	0.000364742	1.3
311	ENSG00000164611	*PTTG1*	2	7.66E-06	0.000364742	1.37
312	ENSG00000146670	*CDCA5*	2	7.66E-06	0.000364742	1.49
313	ENSG00000115165	*CYTIP*	2	7.66E-06	0.000364742	1.52
314	ENSG00000114405	*C3orf14*	2	7.66E-06	0.000364742	1.64
315	ENSG00000166801	*FAM111A*	2	7.66E-06	0.000364742	1.71
316	ENSG00000106477	*CEP41*	2	7.66E-06	0.000364742	1.74
317	ENSG00000162063	*CCNF*	2	7.66E-06	0.000364742	2.01
318	ENSG00000120694	*HSPH1*	2	7.66E-06	0.000364742	2.15
319	ENSG00000075188	*NUP37*	2	7.66E-06	0.000364742	4.3
320	ENSG00000079462	*PAFAH1B3*	3	0.000116806	0.002904762	0
321	ENSG00000131738	*KRT33B*	3	0.000116806	0.002904762	0
322	ENSG00000132429	*POPDC3*	3	0.000116806	0.002904762	0
323	ENSG00000137776	*SLTM*	3	0.000116806	0.002904762	0
324	ENSG00000142731	*PLK4*	3	0.000116806	0.002904762	0
325	ENSG00000187957	*DNER*	3	0.000116806	0.002904762	0
326	ENSG00000196141	*SPATS2L*	3	0.000116806	0.002904762	0
327	ENSG00000241258	*CRCP*	3	0.000116806	0.002904762	0
328	ENSG00000103342	*GSPT1*	3	0.000116806	0.002904762	0.01
329	ENSG00000113739	*STC2*	3	0.000116806	0.002904762	0.01
330	ENSG00000135052	*GOLM1*	3	0.000116806	0.002904762	0.01
331	ENSG00000136518	*ACTL6A*	3	0.000116806	0.002904762	0.01
332	ENSG00000140905	*GCSH*	3	0.000116806	0.002904762	0.01
333	ENSG00000172819	*RARG*	3	0.000116806	0.002904762	0.01
334	ENSG00000184368	*MAP7D2*	3	0.000116806	0.002904762	0.01
335	ENSG00000065548	*ZC3H15*	3	0.000116806	0.002904762	0.02
336	ENSG00000069849	*ATP1B3*	3	0.000116806	0.002904762	0.02
337	ENSG00000126581	*BECN1*	3	0.000116806	0.002904762	0.02
338	ENSG00000140406	*TLNRD1*	3	0.000116806	0.002904762	0.02
339	ENSG00000188342	*GTF2F2*	3	0.000116806	0.002904762	0.02
340	ENSG00000204427	*ABHD16A*	3	0.000116806	0.002904762	0.02
341	ENSG00000170270	*GON7*	3	0.000116806	0.002904762	0.03
342	ENSG00000096384	*HSP90AB1*	3	0.000116806	0.002904762	0.04
343	ENSG00000105323	*HNRNPUL1*	3	0.000116806	0.002904762	0.04
344	ENSG00000159147	*DONSON*	3	0.000116806	0.002904762	0.04
345	ENSG00000115844	*DLX2*	3	0.000116806	0.002904762	0.05
346	ENSG00000142864	*SERBP1*	3	0.000116806	0.002904762	0.06
347	ENSG00000185414	*MRPL30*	3	0.000116806	0.002904762	0.06
348	ENSG00000100418	*DESI1*	3	0.000116806	0.002904762	0.08
349	ENSG00000104356	*POP1*	3	0.000116806	0.002904762	0.08
350	ENSG00000175110	*MRPS22*	3	0.000116806	0.002904762	0.08
351	ENSG00000125398	*SOX9*	3	0.000116806	0.002904762	0.09
352	ENSG00000130052	*STARD8*	3	0.000116806	0.002904762	0.09
353	ENSG00000178878	*APOLD1*	3	0.000116806	0.002904762	0.09
354	ENSG00000053900	*ANAPC4*	3	0.000116806	0.002904762	0.1
355	ENSG00000109685	*NSD2*	3	0.000116806	0.002904762	0.1
356	ENSG00000118939	*UCHL3*	3	0.000116806	0.002904762	0.1
357	ENSG00000127948	*POR*	3	0.000116806	0.002904762	0.1
358	ENSG00000204520	*MICA*	3	0.000116806	0.002904762	0.1
359	ENSG00000004866	*ST7*	3	0.000116806	0.002904762	0.11
360	ENSG00000100519	*PSMC6*	3	0.000116806	0.002904762	0.11
361	ENSG00000135723	*FHOD1*	3	0.000116806	0.002904762	0.11
362	ENSG00000149503	*INCENP*	3	0.000116806	0.002904762	0.11
363	ENSG00000196550	*FAM72A*	3	0.000116806	0.002904762	0.11
364	ENSG00000173848	*NET1*	3	0.000116806	0.002904762	0.12
365	ENSG00000125885	*MCM8*	3	0.000116806	0.002904762	0.13
366	ENSG00000100522	*GNPNAT1*	3	0.000116806	0.002904762	0.15
367	ENSG00000146918	*NCAPG2*	3	0.000116806	0.002904762	0.15
368	ENSG00000149480	*MTA2*	3	0.000116806	0.002904762	0.16
369	ENSG00000160072	*ATAD3B*	3	0.000116806	0.002904762	0.16
370	ENSG00000166401	*SERPINB8*	3	0.000116806	0.002904762	0.16
371	ENSG00000206053	*JPT2*	3	0.000116806	0.002904762	0.16
372	ENSG00000278259	*MYO19*	3	0.000116806	0.002904762	0.16
373	ENSG00000177663	*IL17RA*	3	0.000116806	0.002904762	0.17

374	ENSG00000086827	*ZW10*	3	0.000116806	0.002904762	0.18
375	ENSG00000154328	*NEIL2*	3	0.000116806	0.002904762	0.18
376	ENSG00000032389	*EIPR1*	3	0.000116806	0.002904762	0.2
377	ENSG00000107554	*DNMBP*	3	0.000116806	0.002904762	0.2
378	ENSG00000155959	*VBP1*	3	0.000116806	0.002904762	0.2
379	ENSG00000197299	*BLM*	3	0.000116806	0.002904762	0.2
380	ENSG00000111231	*GPN3*	3	0.000116806	0.002904762	0.21
381	ENSG00000114850	*SSR3*	3	0.000116806	0.002904762	0.21
382	ENSG00000160211	*G6PD*	3	0.000116806	0.002904762	0.21
383	ENSG00000173674	*EIF1AX*	3	0.000116806	0.002904762	0.22
384	ENSG00000177426	*TGIF1*	3	0.000116806	0.002904762	0.22
385	ENSG00000204267	*TAP2*	3	0.000116806	0.002904762	0.22
386	ENSG00000122565	*CBX3*	3	0.000116806	0.002904762	0.23
387	ENSG00000166813	*KIF7*	3	0.000116806	0.002904762	0.24
388	ENSG00000185480	*PARPBP*	3	0.000116806	0.002904762	0.24
389	ENSG00000012048	*BRCA1*	3	0.000116806	0.002904762	0.25
390	ENSG00000107611	*CUBN*	3	0.000116806	0.002904762	0.25
391	ENSG00000164087	*POC1A*	3	0.000116806	0.002904762	0.25
392	ENSG00000176102	*CSTF3*	3	0.000116806	0.002904762	0.25
393	ENSG00000094916	*CBX5*	3	0.000116806	0.002904762	0.26
394	ENSG00000111142	*METAP2*	3	0.000116806	0.002904762	0.26
395	ENSG00000111252	*SH2B3*	3	0.000116806	0.002904762	0.26
396	ENSG00000136699	*SMPD4*	3	0.000116806	0.002904762	0.26
397	ENSG00000108883	*EFTUD2*	3	0.000116806	0.002904762	0.27
398	ENSG00000123416	*TUBA1B*	3	0.000116806	0.002904762	0.27
399	ENSG00000134287	*ARF3*	3	0.000116806	0.002904762	0.28
400	ENSG00000164024	*METAP1*	3	0.000116806	0.002904762	0.28
401	ENSG00000271605	*MILR1*	3	0.000116806	0.002904762	0.28
402	ENSG00000064393	*HIPK2*	3	0.000116806	0.002904762	0.29
403	ENSG00000145386	*CCNA2*	3	0.000116806	0.002904762	0.29
404	ENSG00000162607	*USP1*	3	0.000116806	0.002904762	0.29
405	ENSG00000164045	*CDC25A*	3	0.000116806	0.002904762	0.29
406	ENSG00000177542	*SLC25A22*	3	0.000116806	0.002904762	0.29
407	ENSG00000121390	*PSPC1*	3	0.000116806	0.002904762	0.3
408	ENSG00000177731	*FLII*	3	0.000116806	0.002904762	0.3
409	ENSG00000278535	*DHRS11*	3	0.000116806	0.002904762	0.3
410	ENSG00000074527	*NTN4*	3	0.000116806	0.002904762	0.31
411	ENSG00000092470	*WDR76*	3	0.000116806	0.002904762	0.31
412	ENSG00000160877	*NACC1*	3	0.000116806	0.002904762	0.31
413	ENSG00000054282	*SDCCAG8*	3	0.000116806	0.002904762	0.32
414	ENSG00000164934	*DCAF13*	3	0.000116806	0.002904762	0.32
415	ENSG00000088826	*SMOX*	3	0.000116806	0.002904762	0.33
416	ENSG00000092201	*SUPT16H*	3	0.000116806	0.002904762	0.33
417	ENSG00000136897	*MRPL50*	3	0.000116806	0.002904762	0.33
418	ENSG00000184007	*PTP4A2*	3	0.000116806	0.002904762	0.33
419	ENSG00000188976	*NOC2L*	3	0.000116806	0.002904762	0.33
420	ENSG00000041802	*LSG1*	3	0.000116806	0.002904762	0.34
421	ENSG00000100749	*VRK1*	3	0.000116806	0.002904762	0.34
422	ENSG00000112118	*MCM3*	3	0.000116806	0.002904762	0.34
423	ENSG00000112742	*TTK*	3	0.000116806	0.002904762	0.34
424	ENSG00000123219	*CENPK*	3	0.000116806	0.002904762	0.34
425	ENSG00000145337	*PYURF*	3	0.000116806	0.002904762	0.34
426	ENSG00000175352	*NRIP3*	3	0.000116806	0.002904762	0.34
427	ENSG00000014216	*CAPN1*	3	0.000116806	0.002904762	0.35
428	ENSG00000028116	*VRK2*	3	0.000116806	0.002904762	0.35
429	ENSG00000133119	*RFC3*	3	0.000116806	0.002904762	0.35
430	ENSG00000166562	*SEC11C*	3	0.000116806	0.002904762	0.35
431	ENSG00000188610	*FAM72B*	3	0.000116806	0.002904762	0.35
432	ENSG00000138430	*OLA1*	3	0.000116806	0.002904762	0.36
433	ENSG00000147100	*SLC16A2*	3	0.000116806	0.002904762	0.36
434	ENSG00000150551	*LYPD1*	3	0.000116806	0.002904762	0.36
435	ENSG00000166913	*YWHAB*	3	0.000116806	0.002904762	0.36
436	ENSG00000243725	*TTC4*	3	0.000116806	0.002904762	0.36
437	ENSG00000107949	*BCCIP*	3	0.000116806	0.002904762	0.37
438	ENSG00000108511	*HOXB6*	3	0.000116806	0.002904762	0.37
439	ENSG00000143476	*DTL*	3	0.000116806	0.002904762	0.37
440	ENSG00000149262	*INTS4*	3	0.000116806	0.002904762	0.37
441	ENSG00000176619	*LMNB2*	3	0.000116806	0.002904762	0.37
442	ENSG00000273841	*TAF9*	3	0.000116806	0.002904762	0.37
443	ENSG00000080824	*HSP90AA1*	3	0.000116806	0.002904762	0.38
444	ENSG00000157111	*TMEM171*	3	0.000116806	0.002904762	0.38
445	ENSG00000164924	*YWHAZ*	3	0.000116806	0.002904762	0.38
446	ENSG00000175792	*RUVBL1*	3	0.000116806	0.002904762	0.38
447	ENSG00000189057	*FAM111B*	3	0.000116806	0.002904762	0.38
448	ENSG00000059691	*GATB*	3	0.000116806	0.002904762	0.39
449	ENSG00000075702	*WDR62*	3	0.000116806	0.002904762	0.39
450	ENSG00000100479	*POLE2*	3	0.000116806	0.002904762	0.39
451	ENSG00000175643	*RMI2*	3	0.000116806	0.002904762	0.39
452	ENSG00000091140	*DLD*	3	0.000116806	0.002904762	0.4
453	ENSG00000091483	*FH*	3	0.000116806	0.002904762	0.4
454	ENSG00000125319	*C17orf53*	3	0.000116806	0.002904762	0.4
455	ENSG00000127920	*GNG11*	3	0.000116806	0.002904762	0.4
456	ENSG00000128606	*LRRC17*	3	0.000116806	0.002904762	0.4
457	ENSG00000135047	*CTSL*	3	0.000116806	0.002904762	0.4
458	ENSG00000166197	*NOLC1*	3	0.000116806	0.002904762	0.41
459	ENSG00000121621	*KIF18A*	3	0.000116806	0.002904762	0.42
460	ENSG00000130340	*SNX9*	3	0.000116806	0.002904762	0.42
461	ENSG00000162618	*ADGRL4*	3	0.000116806	0.002904762	0.42
462	ENSG00000187699	*C2orf88*	3	0.000116806	0.002904762	0.42
463	ENSG00000066117	*SMARCD1*	3	0.000116806	0.002904762	0.43
464	ENSG00000100813	*ACIN1*	3	0.000116806	0.002904762	0.44
465	ENSG00000159055	*MIS18A*	3	0.000116806	0.002904762	0.44
466	ENSG00000198331	*HYLS1*	3	0.000116806	0.002904762	0.44
467	ENSG00000177479	*ARIH2*	3	0.000116806	0.002904762	0.45

468	ENSG00000043143	*JADE2*	3	0.000116806	0.002904762	0.46
469	ENSG00000061794	*MRPS35*	3	0.000116806	0.002904762	0.46
470	ENSG00000101945	*SUV39H1*	3	0.000116806	0.002904762	0.46
471	ENSG00000109332	*UBE2D3*	3	0.000116806	0.002904762	0.46
472	ENSG00000125347	*IRF1*	3	0.000116806	0.002904762	0.46
473	ENSG00000139620	*KANSL2*	3	0.000116806	0.002904762	0.47
474	ENSG00000228672	*PROB1*	3	0.000116806	0.002904762	0.47
475	ENSG00000235162	*C12orf75*	3	0.000116806	0.002904762	0.47
476	ENSG00000174238	*PITPNA*	3	0.000116806	0.002904762	0.48
477	ENSG00000103326	*CAPN15*	3	0.000116806	0.002904762	0.49
478	ENSG00000134690	*CDCA8*	3	0.000116806	0.002904762	0.49
479	ENSG00000162959	*MEMO1*	3	0.000116806	0.002904762	0.49
480	ENSG00000196155	*PLEKHG4*	3	0.000116806	0.002904762	0.49
481	ENSG00000086189	*DIMT1*	3	0.000116806	0.002904762	0.5
482	ENSG00000087263	*OGFOD1*	3	0.000116806	0.002904762	0.5
483	ENSG00000142002	*DPP9*	3	0.000116806	0.002904762	0.5
484	ENSG00000142230	*SAE1*	3	0.000116806	0.002904762	0.5
485	ENSG00000147419	*CCDC25*	3	0.000116806	0.002904762	0.5
486	ENSG00000101638	*ST8SIA5*	3	0.000116806	0.002904762	0.51
487	ENSG00000115163	*CENPA*	3	0.000116806	0.002904762	0.51
488	ENSG00000124126	*PREX1*	3	0.000116806	0.002904762	0.51
489	ENSG00000058056	*USP13*	3	0.000116806	0.002904762	0.52
490	ENSG00000197451	*HNRNPAB*	3	0.000116806	0.002904762	0.52
491	ENSG00000205208	*C4orf46*	3	0.000116806	0.002904762	0.52
492	ENSG00000212724	*KRTAP2-3*	3	0.000116806	0.002904762	0.52
493	ENSG00000284969	*n_a*	3	0.000116806	0.002904762	0.52
494	ENSG00000101868	*POLA1*	3	0.000116806	0.002904762	0.53
495	ENSG00000157193	*LRP8*	3	0.000116806	0.002904762	0.53
496	ENSG00000168273	*SMIM4*	3	0.000116806	0.002904762	0.53
497	ENSG00000182585	*EPGN*	3	0.000116806	0.002904762	0.53
498	ENSG00000141349	*G6PC3*	3	0.000116806	0.002904762	0.54
499	ENSG00000176170	*n_a*	3	0.000116806	0.002904762	0.54
500	ENSG00000058804	*NDC1*	3	0.000116806	0.002904762	0.55
501	ENSG00000130638	*ATXN10*	3	0.000116806	0.002904762	0.55
502	ENSG00000137868	*STRA6*	3	0.000116806	0.002904762	0.55
503	ENSG00000110031	*LPXN*	3	0.000116806	0.002904762	0.56
504	ENSG00000116761	*CTH*	3	0.000116806	0.002904762	0.56
505	ENSG00000154473	*BUB3*	3	0.000116806	0.002904762	0.56
506	ENSG00000204394	*VARS*	3	0.000116806	0.002904762	0.56
507	ENSG00000047634	*SCML1*	3	0.000116806	0.002904762	0.57
508	ENSG00000083099	*LYRM2*	3	0.000116806	0.002904762	0.57
509	ENSG00000112541	*PDE10A*	3	0.000116806	0.002904762	0.57
510	ENSG00000124207	*CSE1L*	3	0.000116806	0.002904762	0.57
511	ENSG00000138092	*CENPO*	3	0.000116806	0.002904762	0.57
512	ENSG00000187688	*TRPV2*	3	0.000116806	0.002904762	0.57
513	ENSG00000262655	*SPON1*	3	0.000116806	0.002904762	0.57
514	ENSG00000093217	*XYLB*	3	0.000116806	0.002904762	0.58
515	ENSG00000114346	*ECT2*	3	0.000116806	0.002904762	0.58
516	ENSG00000135318	*NT5E*	3	0.000116806	0.002904762	0.58
517	ENSG00000221944	*TIGD1*	3	0.000116806	0.002904762	0.58
518	ENSG00000079785	*DDX1*	3	0.000116806	0.002904762	0.59
519	ENSG00000080573	*COL5A3*	3	0.000116806	0.002904762	0.59
520	ENSG00000115758	*ODC1*	3	0.000116806	0.002904762	0.59
521	ENSG00000130826	*DKC1*	3	0.000116806	0.002904762	0.59
522	ENSG00000117593	*DARS2*	3	0.000116806	0.002904762	0.6
523	ENSG00000130985	*UBA1*	3	0.000116806	0.002904762	0.6
524	ENSG00000161204	*ABCF3*	3	0.000116806	0.002904762	0.6
525	ENSG00000183814	*LIN9*	3	0.000116806	0.002904762	0.6
526	ENSG00000065150	*IPO5*	3	0.000116806	0.002904762	0.61
527	ENSG00000123178	*SPRYD7*	3	0.000116806	0.002904762	0.61
528	ENSG00000152784	*PRDM8*	3	0.000116806	0.002904762	0.61
529	ENSG00000173153	*ESRRA*	3	0.000116806	0.002904762	0.61
530	ENSG00000214357	*NEURL1B*	3	0.000116806	0.002904762	0.61
531	ENSG00000010292	*NCAPD2*	3	0.000116806	0.002904762	0.62
532	ENSG00000091651	*ORC6*	3	0.000116806	0.002904762	0.62
533	ENSG00000166226	*CCT2*	3	0.000116806	0.002904762	0.62
534	ENSG00000077721	*UBE2A*	3	0.000116806	0.002904762	0.63
535	ENSG00000011304	*PTBP1*	3	0.000116806	0.002904762	0.64
536	ENSG00000081051	*AFP*	3	0.000116806	0.002904762	0.64
537	ENSG00000104907	*TRMT1*	3	0.000116806	0.002904762	0.64
538	ENSG00000167513	*CDT1*	3	0.000116806	0.002904762	0.64
539	ENSG00000095319	*NUP188*	3	0.000116806	0.002904762	0.65
540	ENSG00000105486	*LIG1*	3	0.000116806	0.002904762	0.65
541	ENSG00000133134	*BEX2*	3	0.000116806	0.002904762	0.65
542	ENSG00000139354	*GAS2L3*	3	0.000116806	0.002904762	0.65
543	ENSG00000065923	*SLC9A7*	3	0.000116806	0.002904762	0.66
544	ENSG00000090061	*CCNK*	3	0.000116806	0.002904762	0.66
545	ENSG00000110274	*CEP164*	3	0.000116806	0.002904762	0.66
546	ENSG00000140740	*UQCRC2*	3	0.000116806	0.002904762	0.66
547	ENSG00000057252	*SOAT1*	3	0.000116806	0.002904762	0.67
548	ENSG00000127586	*CHTF18*	3	0.000116806	0.002904762	0.67
549	ENSG00000149948	*HMGA2*	3	0.000116806	0.002904762	0.68
550	ENSG00000145907	*G3BP1*	3	0.000116806	0.002904762	0.69
551	ENSG00000100664	*EIF5*	3	0.000116806	0.002904762	0.7
552	ENSG00000138180	*CEP55*	3	0.000116806	0.002904762	0.7
553	ENSG00000164283	*ESM1*	3	0.000116806	0.002904762	0.7
554	ENSG00000103995	*CEP152*	3	0.000116806	0.002904762	0.71
555	ENSG00000145833	*DDX46*	3	0.000116806	0.002904762	0.71
556	ENSG00000108691	*CCL2*	3	0.000116806	0.002904762	0.73
557	ENSG00000146731	*CCT6A*	3	0.000116806	0.002904762	0.73
558	ENSG00000131462	*TUBG1*	3	0.000116806	0.002904762	0.74
559	ENSG00000148634	*HERC4*	3	0.000116806	0.002904762	0.74
560	ENSG00000154319	*FAM167A*	3	0.000116806	0.002904762	0.74
561	ENSG00000197046	*SIGLEC15*	3	0.000116806	0.002904762	0.74

562	ENSG00000057019	*DCBLD2*	3	0.000116806	0.002904762	0.75
563	ENSG00000111058	*ACSS3*	3	0.000116806	0.002904762	0.75
564	ENSG00000115290	*GRB14*	3	0.000116806	0.002904762	0.75
565	ENSG00000130270	*ATP8B3*	3	0.000116806	0.002904762	0.75
566	ENSG00000138778	*CENPE*	3	0.000116806	0.002904762	0.75
567	ENSG00000161888	*SPC24*	3	0.000116806	0.002904762	0.76
568	ENSG00000106462	*EZH2*	3	0.000116806	0.002904762	0.78
569	ENSG00000164318	*EGFLAM*	3	0.000116806	0.002904762	0.78
570	ENSG00000198824	*CHAMP1*	3	0.000116806	0.002904762	0.78
571	ENSG00000079435	*LIPE*	3	0.000116806	0.002904762	0.79
572	ENSG00000144040	*SFXN5*	3	0.000116806	0.002904762	0.8
573	ENSG00000164543	*STK17A*	3	0.000116806	0.002904762	0.8
574	ENSG00000211460	*TSN*	3	0.000116806	0.002904762	0.8
575	ENSG00000154928	*EPHB1*	3	0.000116806	0.002904762	0.83
576	ENSG00000078618	*NRDC*	3	0.000116806	0.002904762	0.87
577	ENSG00000121152	*NCAPH*	3	0.000116806	0.002904762	0.87
578	ENSG00000136875	*PRPF4*	3	0.000116806	0.002904762	0.91
579	ENSG00000138035	*PNPT1*	3	0.000116806	0.002904762	0.92
580	ENSG00000144554	*FANCD2*	3	0.000116806	0.002904762	0.94
581	ENSG00000104147	*OIP5*	3	0.000116806	0.002904762	0.97
582	ENSG00000197208	*SLC22A4*	3	0.000116806	0.002904762	0.97
583	ENSG00000132603	*NIP7*	3	0.000116806	0.002904762	0.99
584	ENSG00000170144	*HNRNPA3*	3	0.000116806	0.002904762	1.02
585	ENSG00000109084	*TMEM97*	3	0.000116806	0.002904762	1.06
586	ENSG00000176834	*VSIG10*	3	0.000116806	0.002904762	1.07
587	ENSG00000117632	*STMN1*	3	0.000116806	0.002904762	1.09
588	ENSG00000070950	*RAD18*	3	0.000116806	0.002904762	1.1
589	ENSG00000138678	*GPAT3*	3	0.000116806	0.002904762	1.11
590	ENSG00000178726	*THBD*	3	0.000116806	0.002904762	1.12
591	ENSG00000136114	*THSD1*	3	0.000116806	0.002904762	1.14
592	ENSG00000051180	*RAD51*	3	0.000116806	0.002904762	1.2
593	ENSG00000094804	*CDC6*	3	0.000116806	0.002904762	1.23
594	ENSG00000142867	*BCL10*	3	0.000116806	0.002904762	1.23
595	ENSG00000180730	*SHISA2*	3	0.000116806	0.002904762	1.24
596	ENSG00000118777	*ABCG2*	3	0.000116806	0.002904762	1.38
597	ENSG00000133216	*EPHB2*	3	0.000116806	0.002904762	1.47
598	ENSG00000144034	*TPRKB*	3	0.000116806	0.002904762	1.47
599	ENSG00000058085	*LAMC2*	3	0.000116806	0.002904762	1.53
600	ENSG00000185862	*EVI2B*	3	0.000116806	0.002904762	1.57
601	ENSG00000144118	*RALB*	3	0.000116806	0.002904762	1.67
602	ENSG00000163376	*KBTBD8*	3	0.000116806	0.002904762	1.75
603	ENSG00000140105	*WARS*	3	0.000116806	0.002904762	1.83
604	ENSG00000197594	*ENPP1*	3	0.000116806	0.002904762	1.88
605	ENSG00000161381	*PLXDC1*	3	0.000116806	0.002904762	1.96
606	ENSG00000197632	*SERPINB2*	3	0.000116806	0.002904762	1.96
607	ENSG00000006468	*ETV1*	3	0.000116806	0.002904762	2.04
608	ENSG00000160886	*LY6K*	3	0.000116806	0.002904762	2.05
609	ENSG00000181195	*PENK*	3	0.000116806	0.002904762	2.3
610	ENSG00000121289	*CEP89*	3	0.000116806	0.002904762	2.45
611	ENSG00000064419	*TNPO3*	4	0.001232527	0.017830102	0
612	ENSG00000082153	*BZW1*	4	0.001232527	0.017830102	0
613	ENSG00000101019	*UQCC1*	4	0.001232527	0.017830102	0
614	ENSG00000109606	*DHX15*	4	0.001232527	0.017830102	0
615	ENSG00000115875	*SRSF7*	4	0.001232527	0.017830102	0
616	ENSG00000116459	*ATP5PB*	4	0.001232527	0.017830102	0
617	ENSG00000129810	*SGO1*	4	0.001232527	0.017830102	0
618	ENSG00000134186	*PRPF38B*	4	0.001232527	0.017830102	0
619	ENSG00000138675	*FGF5*	4	0.001232527	0.017830102	0
620	ENSG00000138698	*RAP1GDS1*	4	0.001232527	0.017830102	0
621	ENSG00000143816	*WNT9A*	4	0.001232527	0.017830102	0
622	ENSG00000144355	*DLX1*	4	0.001232527	0.017830102	0
623	ENSG00000154736	*ADAMTS5*	4	0.001232527	0.017830102	0
624	ENSG00000167491	*GATAD2A*	4	0.001232527	0.017830102	0
625	ENSG00000174791	*RIN1*	4	0.001232527	0.017830102	0
626	ENSG00000175505	*CLCF1*	4	0.001232527	0.017830102	0
627	ENSG00000175573	*C11orf68*	4	0.001232527	0.017830102	0
628	ENSG00000187555	*USP7*	4	0.001232527	0.017830102	0
629	ENSG00000196678	*ERI2*	4	0.001232527	0.017830102	0
630	ENSG00000197142	*ACSL5*	4	0.001232527	0.017830102	0
631	ENSG00000198763	*MT-ND2*	4	0.001232527	0.017830102	0
632	ENSG00000205683	*DPF3*	4	0.001232527	0.017830102	0
633	ENSG00000050438	*SLC4A8*	4	0.001232527	0.017830102	0.01
634	ENSG00000093144	*ECHDC1*	4	0.001232527	0.017830102	0.01
635	ENSG00000102802	*MEDAG*	4	0.001232527	0.017830102	0.01
636	ENSG00000113575	*PPP2CA*	4	0.001232527	0.017830102	0.01
637	ENSG00000130119	*GNL3L*	4	0.001232527	0.017830102	0.01
638	ENSG00000134697	*GNL2*	4	0.001232527	0.017830102	0.01
639	ENSG00000136527	*TRA2B*	4	0.001232527	0.017830102	0.01
640	ENSG00000140263	*SORD*	4	0.001232527	0.017830102	0.01
641	ENSG00000147164	*SNX12*	4	0.001232527	0.017830102	0.01
642	ENSG00000156535	*CD109*	4	0.001232527	0.017830102	0.01
643	ENSG00000160818	*GPATCH4*	4	0.001232527	0.017830102	0.01
644	ENSG00000108854	*SMURF2*	4	0.001232527	0.017830102	0.02
645	ENSG00000120526	*NUDCD1*	4	0.001232527	0.017830102	0.02
646	ENSG00000132467	*UTP3*	4	0.001232527	0.017830102	0.02
647	ENSG00000151465	*CDC123*	4	0.001232527	0.017830102	0.02
648	ENSG00000100106	*TRIOBP*	4	0.001232527	0.017830102	0.03
649	ENSG00000109689	*STIM2*	4	0.001232527	0.017830102	0.03
650	ENSG00000164104	*HMGB2*	4	0.001232527	0.017830102	0.03
651	ENSG00000167977	*KCTD5*	4	0.001232527	0.017830102	0.03
652	ENSG00000189091	*SF3B3*	4	0.001232527	0.017830102	0.03
653	ENSG00000196793	*ZNF239*	4	0.001232527	0.017830102	0.03
654	ENSG00000072135	*PTPN18*	4	0.001232527	0.017830102	0.04
655	ENSG00000100558	*PLEK2*	4	0.001232527	0.017830102	0.04

656	ENSG00000174738	*NR1D2*	4	0.001232527	0.017830102	0.04
657	ENSG00000185760	*KCNQ5*	4	0.001232527	0.017830102	0.04
658	ENSG00000128340	*RAC2*	4	0.001232527	0.017830102	0.05
659	ENSG00000130066	*SAT1*	4	0.001232527	0.017830102	0.05
660	ENSG00000133030	*MPRIP*	4	0.001232527	0.017830102	0.05
661	ENSG00000133226	*SRRM1*	4	0.001232527	0.017830102	0.05
662	ENSG00000168237	*GLYCTK*	4	0.001232527	0.017830102	0.05
663	ENSG00000170037	*CNTROB*	4	0.001232527	0.017830102	0.05
664	ENSG00000171492	*LRRC8D*	4	0.001232527	0.017830102	0.05
665	ENSG00000178295	*GEN1*	4	0.001232527	0.017830102	0.05
666	ENSG00000182973	*CNOT10*	4	0.001232527	0.017830102	0.05
667	ENSG00000100629	*CEP128*	4	0.001232527	0.017830102	0.06
668	ENSG00000101310	*SEC23B*	4	0.001232527	0.017830102	0.06
669	ENSG00000128059	*PPAT*	4	0.001232527	0.017830102	0.07
670	ENSG00000137825	*ITPKA*	4	0.001232527	0.017830102	0.07
671	ENSG00000180332	*KCTD4*	4	0.001232527	0.017830102	0.07
672	ENSG00000065427	*KARS*	4	0.001232527	0.017830102	0.08
673	ENSG00000160208	*RRP1B*	4	0.001232527	0.017830102	0.08
674	ENSG00000162825	*NBPF20*	4	0.001232527	0.017830102	0.08
675	ENSG00000180817	*PPA1*	4	0.001232527	0.017830102	0.08
676	ENSG00000206560	*ANKRD28*	4	0.001232527	0.017830102	0.08
677	ENSG00000003436	*TFPI*	4	0.001232527	0.017830102	0.09
678	ENSG00000117155	*SSX2IP*	4	0.001232527	0.017830102	0.09
679	ENSG00000093000	*NUP50*	4	0.001232527	0.017830102	0.1
680	ENSG00000102384	*CENPI*	4	0.001232527	0.017830102	0.1
681	ENSG00000102393	*GLA*	4	0.001232527	0.017830102	0.1
682	ENSG00000104332	*SFRP1*	4	0.001232527	0.017830102	0.1
683	ENSG00000038210	*PI4K2B*	4	0.001232527	0.017830102	0.11
684	ENSG00000102230	*PCYT1B*	4	0.001232527	0.017830102	0.11
685	ENSG00000111335	*OAS2*	4	0.001232527	0.017830102	0.11
686	ENSG00000167850	*CD300C*	4	0.001232527	0.017830102	0.11
687	ENSG00000102054	*RBBP7*	4	0.001232527	0.017830102	0.12
688	ENSG00000108064	*TFAM*	4	0.001232527	0.017830102	0.12
689	ENSG00000130830	*MPP1*	4	0.001232527	0.017830102	0.12
690	ENSG00000166477	*LEO1*	4	0.001232527	0.017830102	0.12
691	ENSG00000064199	*SPA17*	4	0.001232527	0.017830102	0.13
692	ENSG00000109321	*AREG*	4	0.001232527	0.017830102	0.13
693	ENSG00000124571	*XPO5*	4	0.001232527	0.017830102	0.13
694	ENSG00000144231	*POLR2D*	4	0.001232527	0.017830102	0.13
695	ENSG00000175806	*MSRA*	4	0.001232527	0.017830102	0.13
696	ENSG00000196584	*XRCC2*	4	0.001232527	0.017830102	0.13
697	ENSG00000068489	*PRR11*	4	0.001232527	0.017830102	0.14
698	ENSG00000128594	*LRRC4*	4	0.001232527	0.017830102	0.14
699	ENSG00000136450	*SRSF1*	4	0.001232527	0.017830102	0.15
700	ENSG00000158006	*PAFAH2*	4	0.001232527	0.017830102	0.15
701	ENSG00000170445	*HARS*	4	0.001232527	0.017830102	0.15
702	ENSG00000018699	*TTC27*	4	0.001232527	0.017830102	0.16
703	ENSG00000103489	*XYLT1*	4	0.001232527	0.017830102	0.16
704	ENSG00000131844	*MCCC2*	4	0.001232527	0.017830102	0.16
705	ENSG00000205476	*CCDC85C*	4	0.001232527	0.017830102	0.16
706	ENSG00000062485	*CS*	4	0.001232527	0.017830102	0.17
707	ENSG00000065183	*WDR3*	4	0.001232527	0.017830102	0.17
708	ENSG00000146242	*TPBG*	4	0.001232527	0.017830102	0.17
709	ENSG00000172244	*C5orf34*	4	0.001232527	0.017830102	0.17
710	ENSG00000078304	*PPP2R5C*	4	0.001232527	0.017830102	0.18
711	ENSG00000132383	*RPA1*	4	0.001232527	0.017830102	0.18
712	ENSG00000151789	*ZNF385D*	4	0.001232527	0.017830102	0.18
713	ENSG00000156802	*ATAD2*	4	0.001232527	0.017830102	0.18
714	ENSG00000171566	*PLRG1*	4	0.001232527	0.017830102	0.18
715	ENSG00000091844	*RGS17*	4	0.001232527	0.017830102	0.19
716	ENSG00000105127	*AKAP8*	4	0.001232527	0.017830102	0.19
717	ENSG00000132313	*MRPL35*	4	0.001232527	0.017830102	0.19
718	ENSG00000168038	*ULK4*	4	0.001232527	0.017830102	0.19
719	ENSG00000181938	*GINS3*	4	0.001232527	0.017830102	0.19
720	ENSG00000184205	*TSPYL2*	4	0.001232527	0.017830102	0.19
721	ENSG00000187325	*TAF9B*	4	0.001232527	0.017830102	0.19
722	ENSG00000197275	*RAD54B*	4	0.001232527	0.017830102	0.19
723	ENSG00000071626	*DAZAP1*	4	0.001232527	0.017830102	0.2
724	ENSG00000111605	*CPSF6*	4	0.001232527	0.017830102	0.2
725	ENSG00000136891	*TEX10*	4	0.001232527	0.017830102	0.2
726	ENSG00000164654	*MIOS*	4	0.001232527	0.017830102	0.2
727	ENSG00000176401	*EID2B*	4	0.001232527	0.017830102	0.2
728	ENSG00000183955	*KMT5A*	4	0.001232527	0.017830102	0.2
729	ENSG00000242265	*PEG10*	4	0.001232527	0.017830102	0.2
730	ENSG00000103591	*AAGAB*	4	0.001232527	0.017830102	0.21
731	ENSG00000146733	*PSPH*	4	0.001232527	0.017830102	0.21
732	ENSG00000165506	*DNAAF2*	4	0.001232527	0.017830102	0.21
733	ENSG00000177889	*UBE2N*	4	0.001232527	0.017830102	0.21
734	ENSG00000055483	*USP36*	4	0.001232527	0.017830102	0.22
735	ENSG00000119685	*TTLL5*	4	0.001232527	0.017830102	0.22
736	ENSG00000140526	*ABHD2*	4	0.001232527	0.017830102	0.22
737	ENSG00000170545	*SMAGP*	4	0.001232527	0.017830102	0.22
738	ENSG00000224578	*HNRNPA1P48*	4	0.001232527	0.017830102	0.22
739	ENSG00000078140	*UBE2K*	4	0.001232527	0.017830102	0.23
740	ENSG00000126216	*TUBGCP3*	4	0.001232527	0.017830102	0.23
741	ENSG00000101000	*PROCR*	4	0.001232527	0.017830102	0.24
742	ENSG00000136261	*BZW2*	4	0.001232527	0.017830102	0.24
743	ENSG00000151287	*TEX30*	4	0.001232527	0.017830102	0.24
744	ENSG00000023572	*GLRX2*	4	0.001232527	0.017830102	0.25
745	ENSG00000095203	*EPB41L4B*	4	0.001232527	0.017830102	0.25
746	ENSG00000112576	*CCND3*	4	0.001232527	0.017830102	0.25
747	ENSG00000116489	*CAPZA1*	4	0.001232527	0.017830102	0.25
748	ENSG00000119487	*MAPKAP1*	4	0.001232527	0.017830102	0.25
749	ENSG00000124596	*OARD1*	4	0.001232527	0.017830102	0.25

750	ENSG00000166979	*EVA1C*	4	0.001232527	0.017830102	0.25
751	ENSG00000204767	*INSYN2B*	4	0.001232527	0.017830102	0.25
752	ENSG00000119865	*CNRIP1*	4	0.001232527	0.017830102	0.26
753	ENSG00000124193	*SRSF6*	4	0.001232527	0.017830102	0.26
754	ENSG00000124541	*RRP36*	4	0.001232527	0.017830102	0.26
755	ENSG00000157064	*NMNAT2*	4	0.001232527	0.017830102	0.26
756	ENSG00000171320	*ESCO2*	4	0.001232527	0.017830102	0.26
757	ENSG00000023171	*GRAMD1B*	4	0.001232527	0.017830102	0.27
758	ENSG00000026559	*KCNG1*	4	0.001232527	0.017830102	0.27
759	ENSG00000088247	*KHSRP*	4	0.001232527	0.017830102	0.27
760	ENSG00000106603	*COA1*	4	0.001232527	0.017830102	0.27
761	ENSG00000108561	*C1QBP*	4	0.001232527	0.017830102	0.27
762	ENSG00000133706	*LARS*	4	0.001232527	0.017830102	0.27
763	ENSG00000143867	*OSR1*	4	0.001232527	0.017830102	0.27
764	ENSG00000147459	*DOCK5*	4	0.001232527	0.017830102	0.27
765	ENSG00000155561	*NUP205*	4	0.001232527	0.017830102	0.27
766	ENSG00000101158	*NELFCD*	4	0.001232527	0.017830102	0.28
767	ENSG00000107815	*TWNK*	4	0.001232527	0.017830102	0.28
768	ENSG00000112029	*FBXO5*	4	0.001232527	0.017830102	0.28
769	ENSG00000137547	*MRPL15*	4	0.001232527	0.017830102	0.28
770	ENSG00000139746	*RBM26*	4	0.001232527	0.017830102	0.28
771	ENSG00000148677	*ANKRD1*	4	0.001232527	0.017830102	0.28
772	ENSG00000148841	*ITPRIP*	4	0.001232527	0.017830102	0.28
773	ENSG00000158156	*XKR8*	4	0.001232527	0.017830102	0.28
774	ENSG00000169895	*SYAP1*	4	0.001232527	0.017830102	0.28
775	ENSG00000014138	*POLA2*	4	0.001232527	0.017830102	0.29
776	ENSG00000155438	*NIFK*	4	0.001232527	0.017830102	0.29
777	ENSG00000196954	*CASP4*	4	0.001232527	0.017830102	0.29
778	ENSG00000204856	*FAM216A*	4	0.001232527	0.017830102	0.29
779	ENSG00000085662	*AKR1B1*	4	0.001232527	0.017830102	0.3
780	ENSG00000125863	*MKKS*	4	0.001232527	0.017830102	0.3
781	ENSG00000134480	*CCNH*	4	0.001232527	0.017830102	0.3
782	ENSG00000141367	*CLTC*	4	0.001232527	0.017830102	0.3
783	ENSG00000146678	*IGFBP1*	4	0.001232527	0.017830102	0.3
784	ENSG00000100714	*MTHFD1*	4	0.001232527	0.017830102	0.31
785	ENSG00000136840	*ST6GALNAC4*	4	0.001232527	0.017830102	0.31
786	ENSG00000139645	*ANKRD52*	4	0.001232527	0.017830102	0.31
787	ENSG00000151247	*EIF4E*	4	0.001232527	0.017830102	0.31
788	ENSG00000164898	*FMC1*	4	0.001232527	0.017830102	0.31
789	ENSG00000035499	*DEPDC1B*	4	0.001232527	0.017830102	0.32
790	ENSG00000112578	*BYSL*	4	0.001232527	0.017830102	0.32
791	ENSG00000125630	*POLR1B*	4	0.001232527	0.017830102	0.32
792	ENSG00000125827	*TMX4*	4	0.001232527	0.017830102	0.32
793	ENSG00000130429	*ARPC1B*	4	0.001232527	0.017830102	0.32
794	ENSG00000140961	*OSGIN1*	4	0.001232527	0.017830102	0.32
795	ENSG00000080608	*PUM3*	4	0.001232527	0.017830102	0.33
796	ENSG00000109674	*NEIL3*	4	0.001232527	0.017830102	0.33
797	ENSG00000120053	*GOT1*	4	0.001232527	0.017830102	0.33
798	ENSG00000151665	*PIGF*	4	0.001232527	0.017830102	0.33
799	ENSG00000171497	*PPID*	4	0.001232527	0.017830102	0.33
800	ENSG00000174842	*GLMN*	4	0.001232527	0.017830102	0.33
801	ENSG00000197961	*ZNF121*	4	0.001232527	0.017830102	0.33
802	ENSG00000005022	*SLC25A5*	4	0.001232527	0.017830102	0.34
803	ENSG00000070831	*CDC42*	4	0.001232527	0.017830102	0.34
804	ENSG00000101670	*LIPG*	4	0.001232527	0.017830102	0.34
805	ENSG00000182667	*NTM*	4	0.001232527	0.017830102	0.34
806	ENSG00000072501	*SMC1A*	4	0.001232527	0.017830102	0.35
807	ENSG00000080839	*RBL1*	4	0.001232527	0.017830102	0.35
808	ENSG00000103876	*FAH*	4	0.001232527	0.017830102	0.35
809	ENSG00000104687	*GSR*	4	0.001232527	0.017830102	0.35
810	ENSG00000151617	*EDNRA*	4	0.001232527	0.017830102	0.35
811	ENSG00000156017	*CARNMT1*	4	0.001232527	0.017830102	0.35
812	ENSG00000157227	*MMP14*	4	0.001232527	0.017830102	0.35
813	ENSG00000176871	*WSB2*	4	0.001232527	0.017830102	0.35
814	ENSG00000181467	*RAP2B*	4	0.001232527	0.017830102	0.35
815	ENSG00000204516	*MICB*	4	0.001232527	0.017830102	0.35
816	ENSG00000214367	*HAUS3*	4	0.001232527	0.017830102	0.35
817	ENSG00000064607	*SUGP2*	4	0.001232527	0.017830102	0.36
818	ENSG00000179041	*RRS1*	4	0.001232527	0.017830102	0.36
819	ENSG00000182963	*GJC1*	4	0.001232527	0.017830102	0.36
820	ENSG00000005189	*REXO5*	4	0.001232527	0.017830102	0.37
821	ENSG00000035681	*NSMAF*	4	0.001232527	0.017830102	0.37
822	ENSG00000102699	*PARP4*	4	0.001232527	0.017830102	0.37
823	ENSG00000106554	*CHCHD3*	4	0.001232527	0.017830102	0.37
824	ENSG00000138347	*MYPN*	4	0.001232527	0.017830102	0.37
825	ENSG00000168487	*BMP1*	4	0.001232527	0.017830102	0.37
826	ENSG00000169213	*RAB3B*	4	0.001232527	0.017830102	0.37
827	ENSG00000170917	*NUDT6*	4	0.001232527	0.017830102	0.37
828	ENSG00000180044	*C3orf80*	4	0.001232527	0.017830102	0.37
829	ENSG00000213066	*FGFR1OP*	4	0.001232527	0.017830102	0.37
830	ENSG00000122483	*CCDC18*	4	0.001232527	0.017830102	0.38
831	ENSG00000135363	*LMO2*	4	0.001232527	0.017830102	0.38
832	ENSG00000146376	*ARHGAP18*	4	0.001232527	0.017830102	0.38
833	ENSG00000154589	*LY96*	4	0.001232527	0.017830102	0.38
834	ENSG00000174780	*SRP72*	4	0.001232527	0.017830102	0.38
835	ENSG00000113356	*POLR3G*	4	0.001232527	0.017830102	0.39
836	ENSG00000271503	*CCL5*	4	0.001232527	0.017830102	0.39
837	ENSG00000116711	*PLA2G4A*	4	0.001232527	0.017830102	0.4
838	ENSG00000161057	*PSMC2*	4	0.001232527	0.017830102	0.4
839	ENSG00000182903	*ZNF721*	4	0.001232527	0.017830102	0.4
840	ENSG00000184110	*EIF3C*	4	0.001232527	0.017830102	0.4
841	ENSG00000198554	*WDHD1*	4	0.001232527	0.017830102	0.4
842	ENSG00000117308	*GALE*	4	0.001232527	0.017830102	0.41
843	ENSG00000130816	*DNMT1*	4	0.001232527	0.017830102	0.41

844	ENSG00000141682	*PMAIP1*	4	0.001232527	0.017830102	0.41
845	ENSG00000148459	*PDSS1*	4	0.001232527	0.017830102	0.41
846	ENSG00000163808	*KIF15*	4	0.001232527	0.017830102	0.41
847	ENSG00000165487	*MICU2*	4	0.001232527	0.017830102	0.41
848	ENSG00000263465	*SRSF8*	4	0.001232527	0.017830102	0.41
849	ENSG00000101574	*METTL4*	4	0.001232527	0.017830102	0.42
850	ENSG00000103423	*DNAJA3*	4	0.001232527	0.017830102	0.42
851	ENSG00000123374	*CDK2*	4	0.001232527	0.017830102	0.42
852	ENSG00000130935	*NOL11*	4	0.001232527	0.017830102	0.42
853	ENSG00000132305	*IMMT*	4	0.001232527	0.017830102	0.42
854	ENSG00000138346	*DNA2*	4	0.001232527	0.017830102	0.42
855	ENSG00000185347	*TEDC1*	4	0.001232527	0.017830102	0.42
856	ENSG00000101096	*NFATC2*	4	0.001232527	0.017830102	0.43
857	ENSG00000105926	*MPP6*	4	0.001232527	0.017830102	0.43
858	ENSG00000107566	*ERLIN1*	4	0.001232527	0.017830102	0.43
859	ENSG00000151466	*SCLT1*	4	0.001232527	0.017830102	0.43
860	ENSG00000158270	*COLEC12*	4	0.001232527	0.017830102	0.43
861	ENSG00000162302	*RPS6KA4*	4	0.001232527	0.017830102	0.43
862	ENSG00000115816	*CEBPZ*	4	0.001232527	0.017830102	0.44
863	ENSG00000124802	*EEF1E1*	4	0.001232527	0.017830102	0.44
864	ENSG00000132361	*CLUH*	4	0.001232527	0.017830102	0.44
865	ENSG00000141560	*FN3KRP*	4	0.001232527	0.017830102	0.44
866	ENSG00000149636	*DSN1*	4	0.001232527	0.017830102	0.44
867	ENSG00000159202	*UBE2Z*	4	0.001232527	0.017830102	0.44
868	ENSG00000006606	*CCL26*	4	0.001232527	0.017830102	0.45
869	ENSG00000095002	*MSH2*	4	0.001232527	0.017830102	0.45
870	ENSG00000105063	*PPP6R1*	4	0.001232527	0.017830102	0.45
871	ENSG00000114270	*COL7A1*	4	0.001232527	0.017830102	0.45
872	ENSG00000126368	*NR1D1*	4	0.001232527	0.017830102	0.45
873	ENSG00000141385	*AFG3L2*	4	0.001232527	0.017830102	0.45
874	ENSG00000157870	*PRXL2B*	4	0.001232527	0.017830102	0.45
875	ENSG00000162062	*TEDC2*	4	0.001232527	0.017830102	0.45
876	ENSG00000164161	*HHIP*	4	0.001232527	0.017830102	0.45
877	ENSG00000048162	*NOP16*	4	0.001232527	0.017830102	0.46
878	ENSG00000119661	*DNAL1*	4	0.001232527	0.017830102	0.46
879	ENSG00000126215	*XRCC3*	4	0.001232527	0.017830102	0.46
880	ENSG00000187741	*FANCA*	4	0.001232527	0.017830102	0.46
881	ENSG00000029153	*ARNTL2*	4	0.001232527	0.017830102	0.47
882	ENSG00000125965	*GDF5*	4	0.001232527	0.017830102	0.47
883	ENSG00000129596	*CDO1*	4	0.001232527	0.017830102	0.47
884	ENSG00000130520	*n_a*	4	0.001232527	0.017830102	0.47
885	ENSG00000175040	*CHST2*	4	0.001232527	0.017830102	0.47
886	ENSG00000258366	*RTEL1*	4	0.001232527	0.017830102	0.47
887	ENSG00000097046	*CDC7*	4	0.001232527	0.017830102	0.48
888	ENSG00000100528	*CNIH1*	4	0.001232527	0.017830102	0.48
889	ENSG00000128708	*HAT1*	4	0.001232527	0.017830102	0.48
890	ENSG00000164134	*NAA15*	4	0.001232527	0.017830102	0.48
891	ENSG00000181649	*PHLDA2*	4	0.001232527	0.017830102	0.48
892	ENSG00000185112	*FAM43A*	4	0.001232527	0.017830102	0.48
893	ENSG00000186575	*NF2*	4	0.001232527	0.017830102	0.48
894	ENSG00000213190	*MLLT11*	4	0.001232527	0.017830102	0.48
895	ENSG00000232119	*MCTS1*	4	0.001232527	0.017830102	0.48
896	ENSG00000085760	*MTIF2*	4	0.001232527	0.017830102	0.49
897	ENSG00000111445	*RFC5*	4	0.001232527	0.017830102	0.49
898	ENSG00000139291	*TMEM19*	4	0.001232527	0.017830102	0.49
899	ENSG00000174611	*KY*	4	0.001232527	0.017830102	0.49
900	ENSG00000260456	*C16orf95*	4	0.001232527	0.017830102	0.49
901	ENSG00000119689	*DLST*	4	0.001232527	0.017830102	0.5
902	ENSG00000135111	*TBX3*	4	0.001232527	0.017830102	0.5
903	ENSG00000137055	*PLAA*	4	0.001232527	0.017830102	0.5
904	ENSG00000147224	*PRPS1*	4	0.001232527	0.017830102	0.5
905	ENSG00000161692	*DBF4B*	4	0.001232527	0.017830102	0.5
906	ENSG00000183840	*GPR39*	4	0.001232527	0.017830102	0.5
907	ENSG00000196581	*AJAP1*	4	0.001232527	0.017830102	0.5
908	ENSG00000163297	*ANTXR2*	4	0.001232527	0.017830102	0.51
909	ENSG00000171951	*SCG2*	4	0.001232527	0.017830102	0.51
910	ENSG00000125148	*MT2A*	4	0.001232527	0.017830102	0.52
911	ENSG00000001036	*FUCA2*	4	0.001232527	0.017830102	0.53
912	ENSG00000006634	*DBF4*	4	0.001232527	0.017830102	0.53
913	ENSG00000090372	*STRN4*	4	0.001232527	0.017830102	0.53
914	ENSG00000091409	*ITGA6*	4	0.001232527	0.017830102	0.53
915	ENSG00000134884	*ARGLU1*	4	0.001232527	0.017830102	0.53
916	ENSG00000197321	*SVIL*	4	0.001232527	0.017830102	0.53
917	ENSG00000132646	*PCNA*	4	0.001232527	0.017830102	0.54
918	ENSG00000127423	*AUNIP*	4	0.001232527	0.017830102	0.55
919	ENSG00000163399	*ATP1A1*	4	0.001232527	0.017830102	0.55
920	ENSG00000101224	*CDC25B*	4	0.001232527	0.017830102	0.56
921	ENSG00000107798	*LIPA*	4	0.001232527	0.017830102	0.56
922	ENSG00000156103	*MMP16*	4	0.001232527	0.017830102	0.56
923	ENSG00000211445	*GPX3*	4	0.001232527	0.017830102	0.56
924	ENSG00000111331	*OAS3*	4	0.001232527	0.017830102	0.57
925	ENSG00000168785	*TSPAN5*	4	0.001232527	0.017830102	0.57
926	ENSG00000139344	*AMDHD1*	4	0.001232527	0.017830102	0.58
927	ENSG00000110876	*SELPLG*	4	0.001232527	0.017830102	0.59
928	ENSG00000221829	*FANCG*	4	0.001232527	0.017830102	0.59
929	ENSG00000119787	*ATL2*	4	0.001232527	0.017830102	0.6
930	ENSG00000198015	*MRPL42*	4	0.001232527	0.017830102	0.6
931	ENSG00000156298	*TSPAN7*	4	0.001232527	0.017830102	0.61
932	ENSG00000183287	*CCBE1*	4	0.001232527	0.017830102	0.61
933	ENSG00000135521	*LTV1*	4	0.001232527	0.017830102	0.62
934	ENSG00000066926	*FECH*	4	0.001232527	0.017830102	0.63
935	ENSG00000120647	*CCDC77*	4	0.001232527	0.017830102	0.63
936	ENSG00000134070	*IRAK2*	4	0.001232527	0.017830102	0.63
937	ENSG00000144395	*CCDC150*	4	0.001232527	0.017830102	0.64

938	ENSG00000130695	*CEP85*	4	0.001232527	0.017830102	0.65
939	ENSG00000133313	*CNDP2*	4	0.001232527	0.017830102	0.65
940	ENSG00000186469	*GNG2*	4	0.001232527	0.017830102	0.66
941	ENSG00000007968	*E2F2*	4	0.001232527	0.017830102	0.67
942	ENSG00000185900	*POMK*	4	0.001232527	0.017830102	0.67
943	ENSG00000166508	*MCM7*	4	0.001232527	0.017830102	0.68
944	ENSG00000128342	*LIF*	4	0.001232527	0.017830102	0.7
945	ENSG00000131153	*GINS2*	4	0.001232527	0.017830102	0.71
946	ENSG00000079313	*REXO1*	4	0.001232527	0.017830102	0.73
947	ENSG00000168883	*USP39*	4	0.001232527	0.017830102	0.73
948	ENSG00000188917	*TRMT2B*	4	0.001232527	0.017830102	0.73
949	ENSG00000255508	*n_a*	4	0.001232527	0.017830102	0.73
950	ENSG00000011485	*PPP5C*	4	0.001232527	0.017830102	0.74
951	ENSG00000065802	*ASB1*	4	0.001232527	0.017830102	0.74
952	ENSG00000165271	*NOL6*	4	0.001232527	0.017830102	0.74
953	ENSG00000258484	*SPESP1*	4	0.001232527	0.017830102	0.74
954	ENSG00000012963	*UBR7*	4	0.001232527	0.017830102	0.75
955	ENSG00000182584	*ACTL10*	4	0.001232527	0.017830102	0.75
956	ENSG00000171302	*CANT1*	4	0.001232527	0.017830102	0.76
957	ENSG00000085999	*RAD54L*	4	0.001232527	0.017830102	0.77
958	ENSG00000092445	*TYRO3*	4	0.001232527	0.017830102	0.79
959	ENSG00000139514	*SLC7A1*	4	0.001232527	0.017830102	0.8
960	ENSG00000143942	*CHAC2*	4	0.001232527	0.017830102	0.8
961	ENSG00000089127	*OAS1*	4	0.001232527	0.017830102	0.81
962	ENSG00000171793	*CTPS1*	4	0.001232527	0.017830102	0.82
963	ENSG00000178999	*AURKB*	4	0.001232527	0.017830102	0.82
964	ENSG00000143512	*HHIPL2*	4	0.001232527	0.017830102	0.83
965	ENSG00000188581	*KRTAP1-1*	4	0.001232527	0.017830102	0.83
966	ENSG00000011007	*ELOA*	4	0.001232527	0.017830102	0.84
967	ENSG00000097021	*ACOT7*	4	0.001232527	0.017830102	0.85
968	ENSG00000080493	*SLC4A4*	4	0.001232527	0.017830102	0.86
969	ENSG00000105968	*H2AFV*	4	0.001232527	0.017830102	0.86
970	ENSG00000134569	*LRP4*	4	0.001232527	0.017830102	0.86
971	ENSG00000144635	*DYNC1LI1*	4	0.001232527	0.017830102	0.86
972	ENSG00000156709	*AIFM1*	4	0.001232527	0.017830102	0.86
973	ENSG00000080986	*NDC80*	4	0.001232527	0.017830102	0.87
974	ENSG00000135324	*MRAP2*	4	0.001232527	0.017830102	0.89
975	ENSG00000116741	*RGS2*	4	0.001232527	0.017830102	0.92
976	ENSG00000115919	*KYNU*	4	0.001232527	0.017830102	0.96
977	ENSG00000164163	*ABCE1*	4	0.001232527	0.017830102	0.96
978	ENSG00000075131	*TIPIN*	4	0.001232527	0.017830102	0.98
979	ENSG00000126088	*UROD*	4	0.001232527	0.017830102	0.98
980	ENSG00000255112	*CHMP1B*	4	0.001232527	0.017830102	0.99
981	ENSG00000108788	*MLX*	4	0.001232527	0.017830102	1
982	ENSG00000163002	*NUP35*	4	0.001232527	0.017830102	1.01
983	ENSG00000101407	*TTI1*	4	0.001232527	0.017830102	1.03
984	ENSG00000127564	*PKMYT1*	4	0.001232527	0.017830102	1.03
985	ENSG00000118503	*TNFAIP3*	4	0.001232527	0.017830102	1.04
986	ENSG00000160949	*TONSL*	4	0.001232527	0.017830102	1.05
987	ENSG00000005302	*MSL3*	4	0.001232527	0.017830102	1.07
988	ENSG00000125375	*DMAC2L*	4	0.001232527	0.017830102	1.1
989	ENSG00000103187	*COTL1*	4	0.001232527	0.017830102	1.13
990	ENSG00000131019	*ULBP3*	4	0.001232527	0.017830102	1.13
991	ENSG00000148840	*PPRC1*	4	0.001232527	0.017830102	1.17
992	ENSG00000069431	*ABCC9*	4	0.001232527	0.017830102	1.19
993	ENSG00000163931	*TKT*	4	0.001232527	0.017830102	1.19
994	ENSG00000136158	*SPRY2*	4	0.001232527	0.017830102	1.21
995	ENSG00000171357	*LURAP1*	4	0.001232527	0.017830102	1.25
996	ENSG00000183421	*RIPK4*	4	0.001232527	0.017830102	1.25
997	ENSG00000119335	*SET*	4	0.001232527	0.017830102	1.27
998	ENSG00000048342	*CC2D2A*	4	0.001232527	0.017830102	1.29
999	ENSG00000155363	*MOV10*	4	0.001232527	0.017830102	1.29
1000	ENSG00000077152	*UBE2T*	4	0.001232527	0.017830102	1.32
1001	ENSG00000106397	*PLOD3*	4	0.001232527	0.017830102	1.37
1002	ENSG00000111581	*NUP107*	4	0.001232527	0.017830102	1.44
1003	ENSG00000198130	*HIBCH*	4	0.001232527	0.017830102	1.47
1004	ENSG00000183891	*TTC32*	4	0.001232527	0.017830102	1.51
1005	ENSG00000002745	*WNT16*	4	0.001232527	0.017830102	1.56
1006	ENSG00000108424	*KPNB1*	4	0.001232527	0.017830102	1.58
1007	ENSG00000156345	*CDK20*	4	0.001232527	0.017830102	1.58
1008	ENSG00000114757	*PEX5L*	4	0.001232527	0.017830102	1.6
1009	ENSG00000100983	*GSS*	4	0.001232527	0.017830102	1.75
1010	ENSG00000214595	*EML6*	4	0.001232527	0.017830102	1.76
1011	ENSG00000115310	*RTN4*	4	0.001232527	0.017830102	1.93
1012	ENSG00000156136	*DCK*	4	0.001232527	0.017830102	1.95
1013	ENSG00000154277	*UCHL1*	4	0.001232527	0.017830102	2.26
1014	ENSG00000172590	*MRPL52*	4	0.001232527	0.017830102	2.27
1015	ENSG00000183048	*SLC25A10*	4	0.001232527	0.017830102	2.29
1016	ENSG00000147408	*CSGALNACT1*	4	0.001232527	0.017830102	2.43
1017	ENSG00000177917	*ARL6IP6*	4	0.001232527	0.017830102	2.85
1018	ENSG00000163781	*TOPBP1*	4	0.001232527	0.017830102	2.89
1019	ENSG00000110721	*CHKA*	4	0.001232527	0.017830102	2.96
1020	ENSG00000124882	*EREG*	4	0.001232527	0.017830102	3.7
1021	ENSG00000108932	*SLC16A6*	4	0.001232527	0.017830102	4.4
1022	ENSG00000172296	*SPTLC3*	4	0.001232527	0.017830102	4.65
1023	ENSG00000116690	*PRG4*	4	0.001232527	0.017830102	4.77
1024	ENSG00000132541	*RIDA*	4	0.001232527	0.017830102	5.23
1025	ENSG00000119917	*IFIT3*	4	0.001232527	0.017830102	5.82
1026	ENSG00000169429	*CXCL8*	4	0.001232527	0.017830102	24.95
