# Supplementary material for: Decoding the differentiation of mesenchymal stem cells into mesangial cells at the transcriptomic level
Source: BMC Genomics. 2020 Jul 7;21:467. doi: 10.1186/s12864-020-06868-5 (PMC7339572; doi:10.1186/s12864-020-06868-5)
Supplement: Supplementary file 5 — Additional file 4. Monotonic ascending pattern genes with DE ≤ 4. [file 12864_2020_6868_MOESM4_ESM.pdf]

Monotonic Ascending pattern genes with DE≤4

	Ensembl Gene ID	Gene Names	DE	p.value	q.value	SVDE
1	ENSG00000025423	*HSD17B6*	0	0	0	0
2	ENSG00000072110	*ACTN1*	0	0	0	0
3	ENSG00000076716	*GPC4*	0	0	0	0
4	ENSG00000091986	*CCDC80*	0	0	0	0
5	ENSG00000099889	*ARVCF*	0	0	0	0
6	ENSG00000106799	*TGFBR1*	0	0	0	0
7	ENSG00000111799	*COL12A1*	0	0	0	0
8	ENSG00000115380	*EFEMP1*	0	0	0	0
9	ENSG00000118523	*CCN2*	0	0	0	0
10	ENSG00000119280	*C1orf198*	0	0	0	0
11	ENSG00000121989	*ACVR2A*	0	0	0	0
12	ENSG00000124212	*PTGIS*	0	0	0	0
13	ENSG00000124766	*SOX4*	0	0	0	0
14	ENSG00000162591	*MEGF6*	0	0	0	0
15	ENSG00000163431	*LMOD1*	0	0	0	0
16	ENSG00000165124	*SVEP1*	0	0	0	0
17	ENSG00000172037	*LAMB2*	0	0	0	0
18	ENSG00000172986	*GXYLT2*	0	0	0	0
19	ENSG00000183723	*CMTM4*	0	0	0	0
20	ENSG00000197381	*ADARB1*	0	0	0	0
21	ENSG00000187955	*COL14A1*	0	0	0	0.01
22	ENSG00000196924	*FLNA*	0	0	0	0.01
23	ENSG00000171388	*APLN*	0	0	0	0.02
24	ENSG00000171522	*PTGER4*	0	0	0	0.02
25	ENSG00000106823	*ECM2*	0	0	0	0.03
26	ENSG00000077157	*PPP1R12B*	0	0	0	0.04
27	ENSG00000274180	*NATD1*	0	0	0	0.04
28	ENSG00000154721	*JAM2*	0	0	0	0.05
29	ENSG00000072952	*MRVI1*	0	0	0	0.06
30	ENSG00000082512	*TRAF5*	0	0	0	0.06
31	ENSG00000060718	*COL11A1*	0	0	0	0.07
32	ENSG00000140092	*FBLN5*	0	0	0	0.07
33	ENSG00000175274	*TP53I11*	0	0	0	0.09
34	ENSG00000204381	*LAYN*	0	0	0	0.09
35	ENSG00000123095	*BHLHE41*	0	0	0	0.12
36	ENSG00000204580	*DDR1*	0	0	0	0.16
37	ENSG00000126803	*HSPA2*	0	0	0	0.2
38	ENSG00000140391	*TSPAN3*	0	0	0	0.24
39	ENSG00000139329	*LUM*	0	0	0	0.25
40	ENSG00000149596	*JPH2*	0	0	0	0.26
41	ENSG00000167244	*IGF2*	0	0	0	0.31
42	ENSG00000136156	*ITM2B*	0	0	0	0.32
43	ENSG00000253276	*CCDC71L*	0	0	0	0.32
44	ENSG00000100439	*ABHD4*	0	0	0	0.33
45	ENSG00000125730	*C3*	0	0	0	0.5
46	ENSG00000022267	*FHL1*	0	0	0	0.53
47	ENSG00000136048	*DRAM1*	0	0	0	0.65
48	ENSG00000152217	*SETBP1*	0	0	0	1.03
49	ENSG00000122642	*FKBP9*	0	0	0	1.27
50	ENSG00000139112	*GABARAPL1*	0	0	0	1.52
51	ENSG00000124225	*PMEPA1*	1	6.38E-07	8.93E-05	0
52	ENSG00000185561	*TLCD2*	1	6.38E-07	8.93E-05	0
53	ENSG00000104936	*DMPK*	1	6.38E-07	8.93E-05	0.01
54	ENSG00000135549	*PKIB*	1	6.38E-07	8.93E-05	0.01
55	ENSG00000183087	*GAS6*	1	6.38E-07	8.93E-05	0.01
56	ENSG00000101280	*ANGPT4*	1	6.38E-07	8.93E-05	0.03
57	ENSG00000171812	*COL8A2*	1	6.38E-07	8.93E-05	0.03
58	ENSG00000115461	*IGFBP5*	1	6.38E-07	8.93E-05	0.05
59	ENSG00000169871	*TRIM56*	1	6.38E-07	8.93E-05	0.05
60	ENSG00000109472	*CPE*	1	6.38E-07	8.93E-05	0.07
61	ENSG00000146477	*SLC22A3*	1	6.38E-07	8.93E-05	0.09
62	ENSG00000064666	*CNN2*	1	6.38E-07	8.93E-05	0.12
63	ENSG00000177363	*LRRN4CL*	1	6.38E-07	8.93E-05	0.12
64	ENSG00000197122	*SRC*	1	6.38E-07	8.93E-05	0.16
65	ENSG00000257335	*MGAM*	1	6.38E-07	8.93E-05	0.18
66	ENSG00000144476	*ACKR3*	1	6.38E-07	8.93E-05	0.21
67	ENSG00000068971	*PPP2R5B*	1	6.38E-07	8.93E-05	0.24
68	ENSG00000113140	*SPARC*	1	6.38E-07	8.93E-05	0.25
69	ENSG00000160285	*LSS*	1	6.38E-07	8.93E-05	0.25
70	ENSG00000120837	*NFYB*	1	6.38E-07	8.93E-05	0.27
71	ENSG00000135926	*TMBIM1*	1	6.38E-07	8.93E-05	0.3
72	ENSG00000139083	*ETV6*	1	6.38E-07	8.93E-05	0.3
73	ENSG00000164099	*PRSS12*	1	6.38E-07	8.93E-05	0.32
74	ENSG00000241399	*CD302*	1	6.38E-07	8.93E-05	0.34
75	ENSG00000142303	*ADAMTS10*	1	6.38E-07	8.93E-05	0.35
76	ENSG00000162458	*FBLIM1*	1	6.38E-07	8.93E-05	0.35
77	ENSG00000153814	*JAZF1*	1	6.38E-07	8.93E-05	0.36
78	ENSG00000254087	*LYN*	1	6.38E-07	8.93E-05	0.36
79	ENSG00000171603	*CLSTN1*	1	6.38E-07	8.93E-05	0.38
80	ENSG00000159423	*ALDH4A1*	1	6.38E-07	8.93E-05	0.39
81	ENSG00000117394	*SLC2A1*	1	6.38E-07	8.93E-05	0.4
82	ENSG00000283154	*IQCJ-SCHIP1*	1	6.38E-07	8.93E-05	0.4
83	ENSG00000089472	*HEPH*	1	6.38E-07	8.93E-05	0.42
84	ENSG00000132003	*ZSWIM4*	1	6.38E-07	8.93E-05	0.43
85	ENSG00000146197	*SCUBE3*	1	6.38E-07	8.93E-05	0.43
86	ENSG00000101825	*MXRA5*	1	6.38E-07	8.93E-05	0.48
87	ENSG00000172201	*ID4*	1	6.38E-07	8.93E-05	0.48
88	ENSG00000081052	*COL4A4*	1	6.38E-07	8.93E-05	0.49
89	ENSG00000162512	*SDC3*	1	6.38E-07	8.93E-05	0.49
90	ENSG00000116774	*OLFML3*	1	6.38E-07	8.93E-05	0.5
91	ENSG00000144711	*IQSEC1*	1	6.38E-07	8.93E-05	0.51

92	ENSG00000171992	*SYNPO*	1	6.38E-07	8.93E-05	0.51
93	ENSG00000186635	*ARAP1*	1	6.38E-07	8.93E-05	0.52
94	ENSG00000182326	*C1S*	1	6.38E-07	8.93E-05	0.54
95	ENSG00000143126	*CELSR2*	1	6.38E-07	8.93E-05	0.55
96	ENSG00000079950	*STX7*	1	6.38E-07	8.93E-05	0.56
97	ENSG00000173599	*PC*	1	6.38E-07	8.93E-05	0.57
98	ENSG00000133619	*KRBA1*	1	6.38E-07	8.93E-05	0.61
99	ENSG00000137809	*ITGA11*	1	6.38E-07	8.93E-05	0.62
100	ENSG00000088756	*ARHGAP28*	1	6.38E-07	8.93E-05	0.63
101	ENSG00000066468	*FGFR2*	1	6.38E-07	8.93E-05	0.82
102	ENSG00000196549	*MME*	1	6.38E-07	8.93E-05	0.98
103	ENSG00000185565	*LSAMP*	1	6.38E-07	8.93E-05	1.04
104	ENSG00000110328	*GALNT18*	1	6.38E-07	8.93E-05	1.07
105	ENSG00000121898	*CPXM2*	1	6.38E-07	8.93E-05	1.19
106	ENSG00000150687	*PRSS23*	1	6.38E-07	8.93E-05	1.52
107	ENSG00000124785	*NRN1*	1	6.38E-07	8.93E-05	1.69
108	ENSG00000100100	*PIK3IP1*	1	6.38E-07	8.93E-05	1.89
109	ENSG00000214717	*ZBED1*	2	8.94E-06	0.000522388	0.32
110	ENSG00000076067	*RBMS2*	2	8.94E-06	0.000522388	0
111	ENSG00000118898	*PPL*	2	8.94E-06	0.000522388	0
112	ENSG00000124006	*OBSL1*	2	8.94E-06	0.000522388	0
113	ENSG00000125629	*INSIG2*	2	8.94E-06	0.000522388	0
114	ENSG00000130821	*SLC6A8*	2	8.94E-06	0.000522388	0
115	ENSG00000135424	*ITGA7*	2	8.94E-06	0.000522388	0
116	ENSG00000137094	*DNAJB5*	2	8.94E-06	0.000522388	0
117	ENSG00000137124	*ALDH1B1*	2	8.94E-06	0.000522388	0
118	ENSG00000172403	*SYNPO2*	2	8.94E-06	0.000522388	0
119	ENSG00000173281	*PPP1R3B*	2	8.94E-06	0.000522388	0
120	ENSG00000179403	*VWA1*	2	8.94E-06	0.000522388	0
121	ENSG00000182575	*NXPH3*	2	8.94E-06	0.000522388	0
122	ENSG00000183283	*DAZAP2*	2	8.94E-06	0.000522388	0
123	ENSG00000184304	*PRKD1*	2	8.94E-06	0.000522388	0
124	ENSG00000184702	*SEPTIN5*	2	8.94E-06	0.000522388	0
125	ENSG00000206190	*ATP10A*	2	8.94E-06	0.000522388	0
126	ENSG00000260230	*FRRS1L*	2	8.94E-06	0.000522388	0
127	ENSG00000125868	*DSTN*	2	8.94E-06	0.000522388	0.01
128	ENSG00000140443	*IGF1R*	2	8.94E-06	0.000522388	0.01
129	ENSG00000157570	*TSPAN18*	2	8.94E-06	0.000522388	0.01
130	ENSG00000173227	*SYT12*	2	8.94E-06	0.000522388	0.01
131	ENSG00000174697	*LEP*	2	8.94E-06	0.000522388	0.01
132	ENSG00000136720	*HS6ST1*	2	8.94E-06	0.000522388	0.02
133	ENSG00000137285	*TUBB2B*	2	8.94E-06	0.000522388	0.03
134	ENSG00000164692	*COL1A2*	2	8.94E-06	0.000522388	0.04
135	ENSG00000171298	*GAA*	2	8.94E-06	0.000522388	0.04
136	ENSG00000161958	*FGF11*	2	8.94E-06	0.000522388	0.05
137	ENSG00000178772	*CPN2*	2	8.94E-06	0.000522388	0.05
138	ENSG00000137699	*TRIM29*	2	8.94E-06	0.000522388	0.06
139	ENSG00000152049	*KCNE4*	2	8.94E-06	0.000522388	0.06
140	ENSG00000077585	*GPR137B*	2	8.94E-06	0.000522388	0.07
141	ENSG00000137507	*LRRC32*	2	8.94E-06	0.000522388	0.07
142	ENSG00000164808	*SPIDR*	2	8.94E-06	0.000522388	0.07
143	ENSG00000171533	*MAP6*	2	8.94E-06	0.000522388	0.07
144	ENSG00000132613	*MTSS2*	2	8.94E-06	0.000522388	0.09
145	ENSG00000135919	*SERPINE2*	2	8.94E-06	0.000522388	0.09
146	ENSG00000090861	*AARS*	2	8.94E-06	0.000522388	0.1
147	ENSG00000138080	*EMILIN1*	2	8.94E-06	0.000522388	0.1
148	ENSG00000158008	*EXTL1*	2	8.94E-06	0.000522388	0.1
149	ENSG00000143499	*SMYD2*	2	8.94E-06	0.000522388	0.12
150	ENSG00000198695	*MT-ND6*	2	8.94E-06	0.000522388	0.14
151	ENSG00000121067	*SPOP*	2	8.94E-06	0.000522388	0.15
152	ENSG00000148926	*ADM*	2	8.94E-06	0.000522388	0.16
153	ENSG00000163083	*INHBB*	2	8.94E-06	0.000522388	0.17
154	ENSG00000117115	*PADI2*	2	8.94E-06	0.000522388	0.18
155	ENSG00000139178	*C1RL*	2	8.94E-06	0.000522388	0.18
156	ENSG00000162520	*SYNC*	2	8.94E-06	0.000522388	0.18
157	ENSG00000072195	*SPEG*	2	8.94E-06	0.000522388	0.2
158	ENSG00000111907	*TPD52L1*	2	8.94E-06	0.000522388	0.2
159	ENSG00000172530	*BANP*	2	8.94E-06	0.000522388	0.2
160	ENSG00000119862	*LGALSL*	2	8.94E-06	0.000522388	0.24
161	ENSG00000154864	*PIEZO2*	2	8.94E-06	0.000522388	0.24
162	ENSG00000166924	*NYAP1*	2	8.94E-06	0.000522388	0.24
163	ENSG00000173546	*CSPG4*	2	8.94E-06	0.000522388	0.25
164	ENSG00000056998	*GYG2*	2	8.94E-06	0.000522388	0.26
165	ENSG00000130176	*CNN1*	2	8.94E-06	0.000522388	0.26
166	ENSG00000108840	*HDAC5*	2	8.94E-06	0.000522388	0.27
167	ENSG00000167693	*NXN*	2	8.94E-06	0.000522388	0.28
168	ENSG00000170370	*EMX2*	2	8.94E-06	0.000522388	0.28
169	ENSG00000090565	*RAB11FIP3*	2	8.94E-06	0.000522388	0.29
170	ENSG00000132000	*PODNL1*	2	8.94E-06	0.000522388	0.3
171	ENSG00000172575	*RASGRP1*	2	8.94E-06	0.000522388	0.3
172	ENSG00000100345	*MYH9*	2	8.94E-06	0.000522388	0.32
173	ENSG00000115828	*QPCT*	2	8.94E-06	0.000522388	0.32
174	ENSG00000150510	*FAM124A*	2	8.94E-06	0.000522388	0.32
175	ENSG00000156804	*FBXO32*	2	8.94E-06	0.000522388	0.32
176	ENSG00000099994	*SUSD2*	2	8.94E-06	0.000522388	0.33
177	ENSG00000123243	*ITIH5*	2	8.94E-06	0.000522388	0.33
178	ENSG00000145687	*SSBP2*	2	8.94E-06	0.000522388	0.33
179	ENSG00000166387	*PPFIBP2*	2	8.94E-06	0.000522388	0.35
180	ENSG00000180914	*OXTR*	2	8.94E-06	0.000522388	0.35
181	ENSG00000198853	*RUSC2*	2	8.94E-06	0.000522388	0.35
182	ENSG00000064042	*LIMCH1*	2	8.94E-06	0.000522388	0.36
183	ENSG00000197635	*DPP4*	2	8.94E-06	0.000522388	0.36
184	ENSG00000099282	*TSPAN15*	2	8.94E-06	0.000522388	0.38
185	ENSG00000136436	*CALCOCO2*	2	8.94E-06	0.000522388	0.38

186	ENSG00000099864	*PALM*	2	8.94E-06	0.000522388	0.39
187	ENSG00000118518	*RNF146*	2	8.94E-06	0.000522388	0.39
188	ENSG00000123240	*OPTN*	2	8.94E-06	0.000522388	0.39
189	ENSG00000169439	*SDC2*	2	8.94E-06	0.000522388	0.4
190	ENSG00000126603	*GLIS2*	2	8.94E-06	0.000522388	0.41
191	ENSG00000197614	*MFAP5*	2	8.94E-06	0.000522388	0.41
192	ENSG00000103044	*HAS3*	2	8.94E-06	0.000522388	0.42
193	ENSG00000134030	*CTIF*	2	8.94E-06	0.000522388	0.42
194	ENSG00000146278	*PNRC1*	2	8.94E-06	0.000522388	0.42
195	ENSG00000156171	*DRAM2*	2	8.94E-06	0.000522388	0.42
196	ENSG00000182749	*PAQR7*	2	8.94E-06	0.000522388	0.42
197	ENSG00000158258	*CLSTN2*	2	8.94E-06	0.000522388	0.43
198	ENSG00000174804	*FZD4*	2	8.94E-06	0.000522388	0.43
199	ENSG00000182985	*CADM1*	2	8.94E-06	0.000522388	0.43
200	ENSG00000112655	*PTK7*	2	8.94E-06	0.000522388	0.44
201	ENSG00000178573	*MAF*	2	8.94E-06	0.000522388	0.44
202	ENSG00000120899	*PTK2B*	2	8.94E-06	0.000522388	0.45
203	ENSG00000122644	*ARL4A*	2	8.94E-06	0.000522388	0.45
204	ENSG00000126778	*SIX1*	2	8.94E-06	0.000522388	0.45
205	ENSG00000135299	*ANKRD6*	2	8.94E-06	0.000522388	0.46
206	ENSG00000108821	*COL1A1*	2	8.94E-06	0.000522388	0.47
207	ENSG00000071282	*LMCD1*	2	8.94E-06	0.000522388	0.48
208	ENSG00000164684	*ZNF704*	2	8.94E-06	0.000522388	0.48
209	ENSG00000183255	*PTTG1IP*	2	8.94E-06	0.000522388	0.48
210	ENSG00000107796	*ACTA2*	2	8.94E-06	0.000522388	0.49
211	ENSG00000139174	*PRICKLE1*	2	8.94E-06	0.000522388	0.49
212	ENSG00000101265	*RASSF2*	2	8.94E-06	0.000522388	0.5
213	ENSG00000112182	*BACH2*	2	8.94E-06	0.000522388	0.5
214	ENSG00000169991	*IFFO2*	2	8.94E-06	0.000522388	0.5
215	ENSG00000181264	*TLCD5*	2	8.94E-06	0.000522388	0.5
216	ENSG00000139567	*ACVRL1*	2	8.94E-06	0.000522388	0.51
217	ENSG00000168453	*HR*	2	8.94E-06	0.000522388	0.53
218	ENSG00000171055	*FEZ2*	2	8.94E-06	0.000522388	0.53
219	ENSG00000170271	*FAXDC2*	2	8.94E-06	0.000522388	0.55
220	ENSG00000157766	*ACAN*	2	8.94E-06	0.000522388	0.56
221	ENSG00000173757	*STAT5B*	2	8.94E-06	0.000522388	0.56
222	ENSG00000170542	*SERPINB9*	2	8.94E-06	0.000522388	0.58
223	ENSG00000197324	*LRP10*	2	8.94E-06	0.000522388	0.58
224	ENSG00000011523	*CEP68*	2	8.94E-06	0.000522388	0.59
225	ENSG00000182118	*FAM89A*	2	8.94E-06	0.000522388	0.59
226	ENSG00000060656	*PTPRU*	2	8.94E-06	0.000522388	0.6
227	ENSG00000142910	*TINAGL1*	2	8.94E-06	0.000522388	0.6
228	ENSG00000196323	*ZBTB44*	2	8.94E-06	0.000522388	0.62
229	ENSG00000004399	*PLXND1*	2	8.94E-06	0.000522388	0.64
230	ENSG00000131171	*SH3BGRL*	2	8.94E-06	0.000522388	0.67
231	ENSG00000149131	*SERPING1*	2	8.94E-06	0.000522388	0.69
232	ENSG00000171105	*INSR*	2	8.94E-06	0.000522388	0.72
233	ENSG00000011465	*DCN*	2	8.94E-06	0.000522388	0.75
234	ENSG00000116679	*IVNS1ABP*	2	8.94E-06	0.000522388	0.76
235	ENSG00000119950	*MXI1*	2	8.94E-06	0.000522388	0.77
236	ENSG00000121964	*GTDC1*	2	8.94E-06	0.000522388	0.77
237	ENSG00000185052	*SLC24A3*	2	8.94E-06	0.000522388	0.77
238	ENSG00000146674	*IGFBP3*	2	8.94E-06	0.000522388	0.78
239	ENSG00000197361	*FBXL22*	2	8.94E-06	0.000522388	0.82
240	ENSG00000155858	*LSM11*	2	8.94E-06	0.000522388	0.83
241	ENSG00000182218	*HHIPL1*	2	8.94E-06	0.000522388	0.84
242	ENSG00000126351	*THRA*	2	8.94E-06	0.000522388	0.86
243	ENSG00000134986	*NREP*	2	8.94E-06	0.000522388	0.86
244	ENSG00000070413	*DGCR2*	2	8.94E-06	0.000522388	0.87
245	ENSG00000144455	*SUMF1*	2	8.94E-06	0.000522388	0.92
246	ENSG00000157837	*SPPL3*	2	8.94E-06	0.000522388	0.94
247	ENSG00000163346	*PBXIP1*	2	8.94E-06	0.000522388	0.94
248	ENSG00000101605	*MYOM1*	2	8.94E-06	0.000522388	1.09
249	ENSG00000171160	*MORN4*	2	8.94E-06	0.000522388	1.1
250	ENSG00000154330	*PGM5*	2	8.94E-06	0.000522388	1.15
251	ENSG00000104321	*TRPA1*	2	8.94E-06	0.000522388	1.24
252	ENSG00000133392	*MYH11*	2	8.94E-06	0.000522388	1.24
253	ENSG00000148175	*STOM*	2	8.94E-06	0.000522388	1.33
254	ENSG00000186866	*POFUT2*	2	8.94E-06	0.000522388	1.48
255	ENSG00000072954	*TMEM38A*	2	8.94E-06	0.000522388	1.51
256	ENSG00000142871	*CCN1*	2	8.94E-06	0.000522388	1.57
257	ENSG00000114853	*ZBTB47*	2	8.94E-06	0.000522388	1.6
258	ENSG00000099204	*ABLIM1*	2	8.94E-06	0.000522388	1.62
259	ENSG00000134243	*SORT1*	2	8.94E-06	0.000522388	2.04
260	ENSG00000006042	*TMEM98*	3	0.000105955	0.003167939	0
261	ENSG00000038002	*AGA*	3	0.000105955	0.003167939	0
262	ENSG00000063660	*GPC1*	3	0.000105955	0.003167939	0
263	ENSG00000121039	*RDH10*	3	0.000105955	0.003167939	0
264	ENSG00000130684	*ZNF337*	3	0.000105955	0.003167939	0
265	ENSG00000136002	*ARHGEF4*	3	0.000105955	0.003167939	0
266	ENSG00000152284	*TCF7L1*	3	0.000105955	0.003167939	0
267	ENSG00000157693	*TMEM268*	3	0.000105955	0.003167939	0
268	ENSG00000162878	*PKDCC*	3	0.000105955	0.003167939	0
269	ENSG00000164066	*INTU*	3	0.000105955	0.003167939	0
270	ENSG00000168763	*CNNM3*	3	0.000105955	0.003167939	0
271	ENSG00000175183	*CSRP2*	3	0.000105955	0.003167939	0
272	ENSG00000181744	*DIPK2A*	3	0.000105955	0.003167939	0
273	ENSG00000182247	*UBE2E2*	3	0.000105955	0.003167939	0
274	ENSG00000188092	*GPR89B*	3	0.000105955	0.003167939	0
275	ENSG00000196843	*ARID5A*	3	0.000105955	0.003167939	0
276	ENSG00000253293	*HOXA10*	3	0.000105955	0.003167939	0
277	ENSG00000000003	*TSPAN6*	3	0.000105955	0.003167939	0.01
278	ENSG00000116273	*PHF13*	3	0.000105955	0.003167939	0.01
279	ENSG00000151729	*SLC25A4*	3	0.000105955	0.003167939	0.01

280	ENSG00000155760	*FZD7*	3	0.000105955	0.003167939	0.01
281	ENSG00000160310	*PRMT2*	3	0.000105955	0.003167939	0.01
282	ENSG00000164188	*RANBP3L*	3	0.000105955	0.003167939	0.01
283	ENSG00000188112	*C6orf132*	3	0.000105955	0.003167939	0.03
284	ENSG00000213614	*HEXA*	3	0.000105955	0.003167939	0.03
285	ENSG00000110042	*DTX4*	3	0.000105955	0.003167939	0.04
286	ENSG00000115457	*IGFBP2*	3	0.000105955	0.003167939	0.04
287	ENSG00000135245	*HILPDA*	3	0.000105955	0.003167939	0.04
288	ENSG00000182253	*SYNM*	3	0.000105955	0.003167939	0.04
289	ENSG00000101608	*MYL12A*	3	0.000105955	0.003167939	0.05
290	ENSG00000145901	*TNIP1*	3	0.000105955	0.003167939	0.05
291	ENSG00000010030	*ETV7*	3	0.000105955	0.003167939	0.07
292	ENSG00000064225	*ST3GAL6*	3	0.000105955	0.003167939	0.07
293	ENSG00000109107	*ALDOC*	3	0.000105955	0.003167939	0.07
294	ENSG00000135823	*STX6*	3	0.000105955	0.003167939	0.07
295	ENSG00000142687	*KIAA0319L*	3	0.000105955	0.003167939	0.07
296	ENSG00000149571	*KIRREL3*	3	0.000105955	0.003167939	0.07
297	ENSG00000174945	*AMZ1*	3	0.000105955	0.003167939	0.07
298	ENSG00000100154	*TTC28*	3	0.000105955	0.003167939	0.08
299	ENSG00000162551	*ALPL*	3	0.000105955	0.003167939	0.08
300	ENSG00000204128	*C2orf72*	3	0.000105955	0.003167939	0.08
301	ENSG00000119938	*PPP1R3C*	3	0.000105955	0.003167939	0.09
302	ENSG00000148180	*GSN*	3	0.000105955	0.003167939	0.09
303	ENSG00000164176	*EDIL3*	3	0.000105955	0.003167939	0.09
304	ENSG00000167930	*FAM234A*	3	0.000105955	0.003167939	0.09
305	ENSG00000178184	*PARD6G*	3	0.000105955	0.003167939	0.09
306	ENSG00000113721	*PDGFRB*	3	0.000105955	0.003167939	0.1
307	ENSG00000197355	*UAP1L1*	3	0.000105955	0.003167939	0.1
308	ENSG00000158186	*MRAS*	3	0.000105955	0.003167939	0.13
309	ENSG00000240771	*ARHGEF25*	3	0.000105955	0.003167939	0.13
310	ENSG00000059804	*SLC2A3*	3	0.000105955	0.003167939	0.14
311	ENSG00000124762	*CDKN1A*	3	0.000105955	0.003167939	0.14
312	ENSG00000173020	*GRK2*	3	0.000105955	0.003167939	0.14
313	ENSG00000176658	*MYO1D*	3	0.000105955	0.003167939	0.14
314	ENSG00000198663	*C6orf89*	3	0.000105955	0.003167939	0.14
315	ENSG00000131398	*KCNC3*	3	0.000105955	0.003167939	0.15
316	ENSG00000158555	*GDPD5*	3	0.000105955	0.003167939	0.15
317	ENSG00000179859	*RNF227*	3	0.000105955	0.003167939	0.15
318	ENSG00000122176	*FMOD*	3	0.000105955	0.003167939	0.16
319	ENSG00000140511	*HAPLN3*	3	0.000105955	0.003167939	0.17
320	ENSG00000165312	*OTUD1*	3	0.000105955	0.003167939	0.17
321	ENSG00000054277	*OPN3*	3	0.000105955	0.003167939	0.18
322	ENSG00000156976	*EIF4A2*	3	0.000105955	0.003167939	0.18
323	ENSG00000164236	*ANKRD33B*	3	0.000105955	0.003167939	0.18
324	ENSG00000187231	*SESTD1*	3	0.000105955	0.003167939	0.18
325	ENSG00000130592	*LSP1*	3	0.000105955	0.003167939	0.19
326	ENSG00000136960	*ENPP2*	3	0.000105955	0.003167939	0.19
327	ENSG00000162849	*KIF26B*	3	0.000105955	0.003167939	0.19
328	ENSG00000173801	*JUP*	3	0.000105955	0.003167939	0.19
329	ENSG00000101400	*SNTA1*	3	0.000105955	0.003167939	0.2
330	ENSG00000117791	*MARC2.*	3	0.000105955	0.003167939	0.2
331	ENSG00000123427	*EEF1AKMT3*	3	0.000105955	0.003167939	0.2
332	ENSG00000136274	*NACAD*	3	0.000105955	0.003167939	0.2
333	ENSG00000147533	*GOLGA7*	3	0.000105955	0.003167939	0.2
334	ENSG00000148344	*PTGES*	3	0.000105955	0.003167939	0.2
335	ENSG00000171680	*PLEKHG5*	3	0.000105955	0.003167939	0.2
336	ENSG00000184481	*FOXO4*	3	0.000105955	0.003167939	0.2
337	ENSG00000105290	*APLP1*	3	0.000105955	0.003167939	0.21
338	ENSG00000120594	*PLXDC2*	3	0.000105955	0.003167939	0.21
339	ENSG00000129353	*SLC44A2*	3	0.000105955	0.003167939	0.21
340	ENSG00000130024	*PHF10*	3	0.000105955	0.003167939	0.21
341	ENSG00000134198	*TSPAN2*	3	0.000105955	0.003167939	0.21
342	ENSG00000182168	*UNC5C*	3	0.000105955	0.003167939	0.21
343	ENSG00000221869	*CEBPD*	3	0.000105955	0.003167939	0.21
344	ENSG00000122547	*EEPD1*	3	0.000105955	0.003167939	0.22
345	ENSG00000175899	*A2M*	3	0.000105955	0.003167939	0.22
346	ENSG00000053254	*FOXN3*	3	0.000105955	0.003167939	0.23
347	ENSG00000113319	*RASGRF2*	3	0.000105955	0.003167939	0.23
348	ENSG00000157240	*FZD1*	3	0.000105955	0.003167939	0.23
349	ENSG00000163516	*ANKZF1*	3	0.000105955	0.003167939	0.23
350	ENSG00000159461	*AMFR*	3	0.000105955	0.003167939	0.24
351	ENSG00000141580	*WDR45B*	3	0.000105955	0.003167939	0.25
352	ENSG00000105664	*COMP*	3	0.000105955	0.003167939	0.26
353	ENSG00000127241	*MASP1*	3	0.000105955	0.003167939	0.27
354	ENSG00000151067	*CACNA1C*	3	0.000105955	0.003167939	0.27
355	ENSG00000174132	*FAM174A*	3	0.000105955	0.003167939	0.27
356	ENSG00000176485	*PLAAT3*	3	0.000105955	0.003167939	0.27
357	ENSG00000152767	*FARP1*	3	0.000105955	0.003167939	0.28
358	ENSG00000165507	*DEPP1*	3	0.000105955	0.003167939	0.28
359	ENSG00000007866	*TEAD3*	3	0.000105955	0.003167939	0.29
360	ENSG00000135070	*ISCA1*	3	0.000105955	0.003167939	0.29
361	ENSG00000135916	*ITM2C*	3	0.000105955	0.003167939	0.29
362	ENSG00000166448	*TMEM130*	3	0.000105955	0.003167939	0.29
363	ENSG00000170153	*RNF150*	3	0.000105955	0.003167939	0.29
364	ENSG00000158292	*GPR153*	3	0.000105955	0.003167939	0.3
365	ENSG00000160445	*ZER1*	3	0.000105955	0.003167939	0.3
366	ENSG00000116157	*GPX7*	3	0.000105955	0.003167939	0.31
367	ENSG00000154511	*DIPK1A*	3	0.000105955	0.003167939	0.31
368	ENSG00000250644	*n_a*	3	0.000105955	0.003167939	0.31
369	ENSG00000131791	*PRKAB2*	3	0.000105955	0.003167939	0.32
370	ENSG00000175220	*ARHGAP1*	3	0.000105955	0.003167939	0.32
371	ENSG00000012171	*SEMA3B*	3	0.000105955	0.003167939	0.33
372	ENSG00000107331	*ABCA2*	3	0.000105955	0.003167939	0.33
373	ENSG00000164093	*PITX2*	3	0.000105955	0.003167939	0.33

374	ENSG00000165996	*HACD1*	3	0.000105955	0.003167939	0.33
375	ENSG00000068976	*PYGM*	3	0.000105955	0.003167939	0.34
376	ENSG00000135636	*DYSF*	3	0.000105955	0.003167939	0.35
377	ENSG00000013288	*MAN2B2*	3	0.000105955	0.003167939	0.36
378	ENSG00000107738	*VSIR*	3	0.000105955	0.003167939	0.36
379	ENSG00000130703	*OSBPL2*	3	0.000105955	0.003167939	0.36
380	ENSG00000181588	*MEX3D*	3	0.000105955	0.003167939	0.36
381	ENSG00000185499	*MUC1*	3	0.000105955	0.003167939	0.36
382	ENSG00000075240	*GRAMD4*	3	0.000105955	0.003167939	0.37
383	ENSG00000082196	*C1QTNF3*	3	0.000105955	0.003167939	0.37
384	ENSG00000109944	*JHY*	3	0.000105955	0.003167939	0.37
385	ENSG00000128294	*TPST2*	3	0.000105955	0.003167939	0.37
386	ENSG00000135452	*TSPAN31*	3	0.000105955	0.003167939	0.37
387	ENSG00000188042	*ARL4C*	3	0.000105955	0.003167939	0.37
388	ENSG00000144647	*POMGNT2*	3	0.000105955	0.003167939	0.38
389	ENSG00000184545	*DUSP8*	3	0.000105955	0.003167939	0.38
390	ENSG00000153179	*RASSF3*	3	0.000105955	0.003167939	0.39
391	ENSG00000169231	*THBS3*	3	0.000105955	0.003167939	0.39
392	ENSG00000100504	*PYGL*	3	0.000105955	0.003167939	0.4
393	ENSG00000109743	*BST1*	3	0.000105955	0.003167939	0.4
394	ENSG00000135929	*CYP27A1*	3	0.000105955	0.003167939	0.4
395	ENSG00000144218	*AFF3*	3	0.000105955	0.003167939	0.4
396	ENSG00000144681	*STAC*	3	0.000105955	0.003167939	0.4
397	ENSG00000154553	*PDLIM3*	3	0.000105955	0.003167939	0.4
398	ENSG00000187479	*C11orf96*	3	0.000105955	0.003167939	0.4
399	ENSG00000163430	*FSTL1*	3	0.000105955	0.003167939	0.41
400	ENSG00000198732	*SMOC1*	3	0.000105955	0.003167939	0.41
401	ENSG00000108352	*RAPGEFL1*	3	0.000105955	0.003167939	0.42
402	ENSG00000157978	*LDLRAP1*	3	0.000105955	0.003167939	0.42
403	ENSG00000034053	*APBA2*	3	0.000105955	0.003167939	0.43
404	ENSG00000138433	*CIR1*	3	0.000105955	0.003167939	0.43
405	ENSG00000143995	*MEIS1*	3	0.000105955	0.003167939	0.43
406	ENSG00000198542	*ITGBL1*	3	0.000105955	0.003167939	0.43
407	ENSG00000077420	*APBB1IP*	3	0.000105955	0.003167939	0.44
408	ENSG00000083750	*RRAGB*	3	0.000105955	0.003167939	0.44
409	ENSG00000142583	*SLC2A5*	3	0.000105955	0.003167939	0.44
410	ENSG00000164751	*PEX2*	3	0.000105955	0.003167939	0.44
411	ENSG00000070404	*FSTL3*	3	0.000105955	0.003167939	0.45
412	ENSG00000143409	*MINDY1*	3	0.000105955	0.003167939	0.45
413	ENSG00000148737	*TCF7L2*	3	0.000105955	0.003167939	0.46
414	ENSG00000069535	*MAOB*	3	0.000105955	0.003167939	0.47
415	ENSG00000104324	*CPQ*	3	0.000105955	0.003167939	0.47
416	ENSG00000119673	*ACOT2*	3	0.000105955	0.003167939	0.47
417	ENSG00000119979	*FAM45A*	3	0.000105955	0.003167939	0.47
418	ENSG00000145681	*HAPLN1*	3	0.000105955	0.003167939	0.47
419	ENSG00000176171	*BNIP3*	3	0.000105955	0.003167939	0.47
420	ENSG00000184922	*FMNL1*	3	0.000105955	0.003167939	0.47
421	ENSG00000112079	*STK38*	3	0.000105955	0.003167939	0.49
422	ENSG00000147027	*TMEM47*	3	0.000105955	0.003167939	0.49
423	ENSG00000204852	*TCTN1*	3	0.000105955	0.003167939	0.49
424	ENSG00000071967	*CYBRD1*	3	0.000105955	0.003167939	0.5
425	ENSG00000213625	*LEPROT*	3	0.000105955	0.003167939	0.5
426	ENSG00000275023	*MLLT6*	3	0.000105955	0.003167939	0.5
427	ENSG00000164050	*PLXNB1*	3	0.000105955	0.003167939	0.51
428	ENSG00000167641	*PPP1R14A*	3	0.000105955	0.003167939	0.51
429	ENSG00000183346	*CABCOCO1*	3	0.000105955	0.003167939	0.51
430	ENSG00000241839	*PLEKHO2*	3	0.000105955	0.003167939	0.51
431	ENSG00000162645	*GBP2*	3	0.000105955	0.003167939	0.52
432	ENSG00000168542	*COL3A1*	3	0.000105955	0.003167939	0.52
433	ENSG00000198728	*LDB1*	3	0.000105955	0.003167939	0.53
434	ENSG00000148730	*EIF4EBP2*	3	0.000105955	0.003167939	0.54
435	ENSG00000088367	*EPB41L1*	3	0.000105955	0.003167939	0.55
436	ENSG00000109063	*MYH3*	3	0.000105955	0.003167939	0.55
437	ENSG00000164442	*CITED2*	3	0.000105955	0.003167939	0.55
438	ENSG00000164694	*FNDC1*	3	0.000105955	0.003167939	0.55
439	ENSG00000166343	*MSS51*	3	0.000105955	0.003167939	0.55
440	ENSG00000011454	*RABGAP1*	3	0.000105955	0.003167939	0.56
441	ENSG00000111674	*ENO2*	3	0.000105955	0.003167939	0.56
442	ENSG00000131634	*TMEM204*	3	0.000105955	0.003167939	0.56
443	ENSG00000105137	*SYDE1*	3	0.000105955	0.003167939	0.57
444	ENSG00000138050	*THUMPD2*	3	0.000105955	0.003167939	0.57
445	ENSG00000139266	*MARCHF9.*	3	0.000105955	0.003167939	0.57
446	ENSG00000149115	*TNKS1BP1*	3	0.000105955	0.003167939	0.57
447	ENSG00000084731	*KIF3C*	3	0.000105955	0.003167939	0.58
448	ENSG00000180447	*GAS1*	3	0.000105955	0.003167939	0.58
449	ENSG00000197043	*ANXA6*	3	0.000105955	0.003167939	0.59
450	ENSG00000131067	*GGT7*	3	0.000105955	0.003167939	0.6
451	ENSG00000159251	*ACTC1*	3	0.000105955	0.003167939	0.6
452	ENSG00000141736	*ERBB2*	3	0.000105955	0.003167939	0.61
453	ENSG00000164574	*GALNT10*	3	0.000105955	0.003167939	0.61
454	ENSG00000073910	*FRY*	3	0.000105955	0.003167939	0.62
455	ENSG00000109501	*WFS1*	3	0.000105955	0.003167939	0.62
456	ENSG00000143845	*ETNK2*	3	0.000105955	0.003167939	0.62
457	ENSG00000010610	*CD4*	3	0.000105955	0.003167939	0.65
458	ENSG00000244486	*SCARF2*	3	0.000105955	0.003167939	0.65
459	ENSG00000116954	*RRAGC*	3	0.000105955	0.003167939	0.66
460	ENSG00000121671	*CRY2*	3	0.000105955	0.003167939	0.66
461	ENSG00000140285	*FGF7*	3	0.000105955	0.003167939	0.66
462	ENSG00000235750	*KIAA0040*	3	0.000105955	0.003167939	0.67
463	ENSG00000074964	*ARHGEF10L*	3	0.000105955	0.003167939	0.68
464	ENSG00000100139	*MICALL1*	3	0.000105955	0.003167939	0.68
465	ENSG00000101210	*EEF1A2*	3	0.000105955	0.003167939	0.68
466	ENSG00000159128	*IFNGR2*	3	0.000105955	0.003167939	0.69
467	ENSG00000163661	*PTX3*	3	0.000105955	0.003167939	0.7

468	ENSG00000080845	*DLGAP4*	3	0.000105955	0.003167939	0.71
469	ENSG00000136379	*ABHD17C*	3	0.000105955	0.003167939	0.71
470	ENSG00000162604	*TM2D1*	3	0.000105955	0.003167939	0.71
471	ENSG00000165409	*TSHR*	3	0.000105955	0.003167939	0.73
472	ENSG00000123096	*SSPN*	3	0.000105955	0.003167939	0.76
473	ENSG00000131196	*NFATC1*	3	0.000105955	0.003167939	0.78
474	ENSG00000071051	*NCK2*	3	0.000105955	0.003167939	0.82
475	ENSG00000101938	*CHRDL1*	3	0.000105955	0.003167939	0.82
476	ENSG00000055211	*GINM1*	3	0.000105955	0.003167939	0.85
477	ENSG00000204851	*PNMA8B*	3	0.000105955	0.003167939	0.86
478	ENSG00000173175	*ADCY5*	3	0.000105955	0.003167939	0.91
479	ENSG00000177169	*ULK1*	3	0.000105955	0.003167939	0.91
480	ENSG00000169122	*FAM110B*	3	0.000105955	0.003167939	0.92
481	ENSG00000163638	*ADAMTS9*	3	0.000105955	0.003167939	0.97
482	ENSG00000134202	*GSTM3*	3	0.000105955	0.003167939	0.98
483	ENSG00000138449	*SLC40A1*	3	0.000105955	0.003167939	0.98
484	ENSG00000067992	*PDK3*	3	0.000105955	0.003167939	1.04
485	ENSG00000105429	*MEGF8*	3	0.000105955	0.003167939	1.04
486	ENSG00000146267	*FAXC*	3	0.000105955	0.003167939	1.06
487	ENSG00000189164	*ZNF527*	3	0.000105955	0.003167939	1.06
488	ENSG00000183111	*ARHGEF37*	3	0.000105955	0.003167939	1.12
489	ENSG00000140044	*JDP2*	3	0.000105955	0.003167939	1.13
490	ENSG00000149451	*ADAM33*	3	0.000105955	0.003167939	1.13
491	ENSG00000167971	*CASKIN1*	3	0.000105955	0.003167939	1.16
492	ENSG00000205903	*ZNF316*	3	0.000105955	0.003167939	1.21
493	ENSG00000243646	*IL10RB*	3	0.000105955	0.003167939	1.26
494	ENSG00000159784	*FAM131B*	3	0.000105955	0.003167939	1.31
495	ENSG00000259330	*INAFM2*	3	0.000105955	0.003167939	1.71
496	ENSG00000117643	*MAN1C1*	3	0.000105955	0.003167939	1.93
497	ENSG00000077943	*ITGA8*	3	0.000105955	0.003167939	2.01
498	ENSG00000102349	*KLF8*	3	0.000105955	0.003167939	2.97
499	ENSG00000179344	*HLA-DQB1*	3	0.000105955	0.003167939	3.63
500	ENSG00000102181	*CD99L2*	3	0.000105955	0.003167939	4.61
501	ENSG00000015153	*YAF2*	4	0.001065296	0.016909828	0
502	ENSG00000037637	*FBXO42*	4	0.001065296	0.016909828	0
503	ENSG00000057935	*MTA3*	4	0.001065296	0.016909828	0
504	ENSG00000074590	*NUAK1*	4	0.001065296	0.016909828	0
505	ENSG00000082781	*ITGB5*	4	0.001065296	0.016909828	0
506	ENSG00000099949	*LZTR1*	4	0.001065296	0.016909828	0
507	ENSG00000102024	*PLS3*	4	0.001065296	0.016909828	0
508	ENSG00000102445	*RUBCNL*	4	0.001065296	0.016909828	0
509	ENSG00000103888	*CEMIP*	4	0.001065296	0.016909828	0
510	ENSG00000104047	*DTWD1*	4	0.001065296	0.016909828	0
511	ENSG00000107159	*CA9*	4	0.001065296	0.016909828	0
512	ENSG00000108239	*TBC1D12*	4	0.001065296	0.016909828	0
513	ENSG00000112210	*RAB23*	4	0.001065296	0.016909828	0
514	ENSG00000117859	*OSBPL9*	4	0.001065296	0.016909828	0
515	ENSG00000118705	*RPN2*	4	0.001065296	0.016909828	0
516	ENSG00000119630	*PGF*	4	0.001065296	0.016909828	0
517	ENSG00000120254	*MTHFD1L*	4	0.001065296	0.016909828	0
518	ENSG00000126561	*STAT5A*	4	0.001065296	0.016909828	0
519	ENSG00000129116	*PALLD*	4	0.001065296	0.016909828	0
520	ENSG00000149591	*TAGLN*	4	0.001065296	0.016909828	0
521	ENSG00000151778	*SERP2*	4	0.001065296	0.016909828	0
522	ENSG00000152894	*PTPRK*	4	0.001065296	0.016909828	0
523	ENSG00000154978	*VOPP1*	4	0.001065296	0.016909828	0
524	ENSG00000156026	*MCU*	4	0.001065296	0.016909828	0
525	ENSG00000159176	*CSRP1*	4	0.001065296	0.016909828	0
526	ENSG00000160255	*ITGB2*	4	0.001065296	0.016909828	0
527	ENSG00000198420	*TCAF1*	4	0.001065296	0.016909828	0
528	ENSG00000211455	*STK38L*	4	0.001065296	0.016909828	0
529	ENSG00000050165	*DKK3*	4	0.001065296	0.016909828	0.01
530	ENSG00000078902	*TOLLIP*	4	0.001065296	0.016909828	0.01
531	ENSG00000117519	*CNN3*	4	0.001065296	0.016909828	0.01
532	ENSG00000135956	*TMEM127*	4	0.001065296	0.016909828	0.01
533	ENSG00000185633	*NDUFA4L2*	4	0.001065296	0.016909828	0.01
534	ENSG00000065989	*n_a*	4	0.001065296	0.016909828	0.02
535	ENSG00000084652	*TXLNA*	4	0.001065296	0.016909828	0.02
536	ENSG00000090924	*PLEKHG2*	4	0.001065296	0.016909828	0.02
537	ENSG00000254726	*MEX3A*	4	0.001065296	0.016909828	0.02
538	ENSG00000046651	*OFD1*	4	0.001065296	0.016909828	0.03
539	ENSG00000101335	*MYL9*	4	0.001065296	0.016909828	0.03
540	ENSG00000107438	*PDLIM1*	4	0.001065296	0.016909828	0.03
541	ENSG00000116786	*PLEKHM2*	4	0.001065296	0.016909828	0.03
542	ENSG00000141447	*OSBPL1A*	4	0.001065296	0.016909828	0.03
543	ENSG00000163251	*FZD5*	4	0.001065296	0.016909828	0.03
544	ENSG00000168118	*RAB4A*	4	0.001065296	0.016909828	0.03
545	ENSG00000178776	*C5orf46*	4	0.001065296	0.016909828	0.03
546	ENSG00000205309	*NT5M*	4	0.001065296	0.016909828	0.03
547	ENSG00000205978	*NYNRIN*	4	0.001065296	0.016909828	0.03
548	ENSG00000214944	*ARHGEF28*	4	0.001065296	0.016909828	0.03
549	ENSG00000104870	*FCGRT*	4	0.001065296	0.016909828	0.04
550	ENSG00000107551	*RASSF4*	4	0.001065296	0.016909828	0.04
551	ENSG00000117139	*KDM5B*	4	0.001065296	0.016909828	0.04
552	ENSG00000118707	*TGIF2*	4	0.001065296	0.016909828	0.04
553	ENSG00000122367	*LDB3*	4	0.001065296	0.016909828	0.04
554	ENSG00000129625	*REEP5*	4	0.001065296	0.016909828	0.04
555	ENSG00000140577	*CRTC3*	4	0.001065296	0.016909828	0.04
556	ENSG00000143382	*ADAMTSL4*	4	0.001065296	0.016909828	0.04
557	ENSG00000160703	*NLRX1*	4	0.001065296	0.016909828	0.04
558	ENSG00000162614	*NEXN*	4	0.001065296	0.016909828	0.04
559	ENSG00000167107	*ACSF2*	4	0.001065296	0.016909828	0.04
560	ENSG00000175084	*DES*	4	0.001065296	0.016909828	0.04
561	ENSG00000060069	*CTDP1*	4	0.001065296	0.016909828	0.05

562	ENSG00000062716	*VMP1*	4	0.001065296	0.016909828	0.05
563	ENSG00000152601	*MBNL1*	4	0.001065296	0.016909828	0.05
564	ENSG00000154556	*SORBS2*	4	0.001065296	0.016909828	0.05
565	ENSG00000169515	*CCDC8*	4	0.001065296	0.016909828	0.05
566	ENSG00000187244	*BCAM*	4	0.001065296	0.016909828	0.05
567	ENSG00000142733	*MAP3K6*	4	0.001065296	0.016909828	0.06
568	ENSG00000151422	*FER*	4	0.001065296	0.016909828	0.06
569	ENSG00000188997	*KCTD21*	4	0.001065296	0.016909828	0.06
570	ENSG00000005379	*TSPOAP1*	4	0.001065296	0.016909828	0.07
571	ENSG00000101871	*MID1*	4	0.001065296	0.016909828	0.07
572	ENSG00000116128	*BCL9*	4	0.001065296	0.016909828	0.07
573	ENSG00000131668	*BARX1*	4	0.001065296	0.016909828	0.07
574	ENSG00000163815	*CLEC3B*	4	0.001065296	0.016909828	0.07
575	ENSG00000180340	*FZD2*	4	0.001065296	0.016909828	0.07
576	ENSG00000275074	*NUDT18*	4	0.001065296	0.016909828	0.07
577	ENSG00000104881	*PPP1R13L*	4	0.001065296	0.016909828	0.08
578	ENSG00000143554	*SLC27A3*	4	0.001065296	0.016909828	0.08
579	ENSG00000155893	*PXYLP1*	4	0.001065296	0.016909828	0.08
580	ENSG00000157637	*SLC38A10*	4	0.001065296	0.016909828	0.08
581	ENSG00000116117	*PARD3B*	4	0.001065296	0.016909828	0.09
582	ENSG00000119318	*RAD23B*	4	0.001065296	0.016909828	0.09
583	ENSG00000168955	*TM4SF20*	4	0.001065296	0.016909828	0.09
584	ENSG00000170442	*KRT86*	4	0.001065296	0.016909828	0.09
585	ENSG00000004975	*DVL2*	4	0.001065296	0.016909828	0.1
586	ENSG00000012822	*CALCOCO1*	4	0.001065296	0.016909828	0.1
587	ENSG00000109625	*CPZ*	4	0.001065296	0.016909828	0.1
588	ENSG00000134363	*FST*	4	0.001065296	0.016909828	0.1
589	ENSG00000172638	*EFEMP2*	4	0.001065296	0.016909828	0.1
590	ENSG00000186205	*MARC1.*	4	0.001065296	0.016909828	0.1
591	ENSG00000112658	*SRF*	4	0.001065296	0.016909828	0.11
592	ENSG00000235194	*PPP1R3E*	4	0.001065296	0.016909828	0.11
593	ENSG00000133142	*TCEAL4*	4	0.001065296	0.016909828	0.12
594	ENSG00000135205	*CCDC146*	4	0.001065296	0.016909828	0.12
595	ENSG00000181284	*TMEM102*	4	0.001065296	0.016909828	0.12
596	ENSG00000198467	*TPM2*	4	0.001065296	0.016909828	0.12
597	ENSG00000012660	*ELOVL5*	4	0.001065296	0.016909828	0.13
598	ENSG00000122203	*KIAA1191*	4	0.001065296	0.016909828	0.13
599	ENSG00000150347	*ARID5B*	4	0.001065296	0.016909828	0.13
600	ENSG00000167123	*CERCAM*	4	0.001065296	0.016909828	0.13
601	ENSG00000083290	*ULK2*	4	0.001065296	0.016909828	0.14
602	ENSG00000148848	*ADAM12*	4	0.001065296	0.016909828	0.14
603	ENSG00000089351	*GRAMD1A*	4	0.001065296	0.016909828	0.15
604	ENSG00000107816	*LZTS2*	4	0.001065296	0.016909828	0.15
605	ENSG00000121413	*ZSCAN18*	4	0.001065296	0.016909828	0.15
606	ENSG00000138759	*FRAS1*	4	0.001065296	0.016909828	0.15
607	ENSG00000160094	*ZNF362*	4	0.001065296	0.016909828	0.15
608	ENSG00000261150	*EPPK1*	4	0.001065296	0.016909828	0.15
609	ENSG00000163521	*GLB1L*	4	0.001065296	0.016909828	0.16
610	ENSG00000177565	*TBL1XR1*	4	0.001065296	0.016909828	0.16
611	ENSG00000180155	*LYNX1*	4	0.001065296	0.016909828	0.16
612	ENSG00000182923	*CEP63*	4	0.001065296	0.016909828	0.16
613	ENSG00000106327	*TFR2*	4	0.001065296	0.016909828	0.17
614	ENSG00000135622	*SEMA4F*	4	0.001065296	0.016909828	0.17
615	ENSG00000143379	*SETDB1*	4	0.001065296	0.016909828	0.17
616	ENSG00000151743	*AMN1*	4	0.001065296	0.016909828	0.17
617	ENSG00000157833	*GAREM2*	4	0.001065296	0.016909828	0.17
618	ENSG00000162704	*ARPC5*	4	0.001065296	0.016909828	0.17
619	ENSG00000166710	*B2M*	4	0.001065296	0.016909828	0.17
620	ENSG00000175309	*PHYKPL*	4	0.001065296	0.016909828	0.17
621	ENSG00000108861	*DUSP3*	4	0.001065296	0.016909828	0.18
622	ENSG00000137103	*TMEM8B*	4	0.001065296	0.016909828	0.18
623	ENSG00000165152	*TMEM246*	4	0.001065296	0.016909828	0.18
624	ENSG00000172667	*ZMAT3*	4	0.001065296	0.016909828	0.18
625	ENSG00000275993	*SIK1B*	4	0.001065296	0.016909828	0.18
626	ENSG00000074657	*ZNF532*	4	0.001065296	0.016909828	0.19
627	ENSG00000077684	*JADE1*	4	0.001065296	0.016909828	0.19
628	ENSG00000112679	*DUSP22*	4	0.001065296	0.016909828	0.19
629	ENSG00000122707	*RECK*	4	0.001065296	0.016909828	0.19
630	ENSG00000124067	*SLC12A4*	4	0.001065296	0.016909828	0.19
631	ENSG00000170004	*CHD3*	4	0.001065296	0.016909828	0.19
632	ENSG00000174306	*ZHX3*	4	0.001065296	0.016909828	0.19
633	ENSG00000175727	*MLXIP*	4	0.001065296	0.016909828	0.19
634	ENSG00000182916	*TCEAL7*	4	0.001065296	0.016909828	0.19
635	ENSG00000184635	*ZNF93*	4	0.001065296	0.016909828	0.19
636	ENSG00000054793	*ATP9A*	4	0.001065296	0.016909828	0.2
637	ENSG00000100376	*FAM118A*	4	0.001065296	0.016909828	0.2
638	ENSG00000150051	*MKX*	4	0.001065296	0.016909828	0.2
639	ENSG00000153048	*CARHSP1*	4	0.001065296	0.016909828	0.2
640	ENSG00000179954	*SSC5D*	4	0.001065296	0.016909828	0.2
641	ENSG00000188783	*PRELP*	4	0.001065296	0.016909828	0.2
642	ENSG00000071073	*MGAT4A*	4	0.001065296	0.016909828	0.21
643	ENSG00000091972	*CD200*	4	0.001065296	0.016909828	0.21
644	ENSG00000125648	*SLC25A23*	4	0.001065296	0.016909828	0.21
645	ENSG00000131370	*SH3BP5*	4	0.001065296	0.016909828	0.21
646	ENSG00000145916	*RMND5B*	4	0.001065296	0.016909828	0.21
647	ENSG00000153885	*KCTD15*	4	0.001065296	0.016909828	0.21
648	ENSG00000076344	*RGS11*	4	0.001065296	0.016909828	0.22
649	ENSG00000107679	*PLEKHA1*	4	0.001065296	0.016909828	0.22
650	ENSG00000111276	*CDKN1B*	4	0.001065296	0.016909828	0.22
651	ENSG00000143437	*ARNT*	4	0.001065296	0.016909828	0.22
652	ENSG00000163378	*EOGT*	4	0.001065296	0.016909828	0.22
653	ENSG00000171385	*KCND3*	4	0.001065296	0.016909828	0.22
654	ENSG00000172673	*THEMIS*	4	0.001065296	0.016909828	0.22
655	ENSG00000196588	*MRTFA*	4	0.001065296	0.016909828	0.22

656	ENSG00000103742	*IGDCC4*	4	0.001065296	0.016909828	0.23
657	ENSG00000107745	*MICU1*	4	0.001065296	0.016909828	0.23
658	ENSG00000121104	*FAM117A*	4	0.001065296	0.016909828	0.23
659	ENSG00000128268	*MGAT3*	4	0.001065296	0.016909828	0.23
660	ENSG00000143590	*EFNA3*	4	0.001065296	0.016909828	0.23
661	ENSG00000151640	*DPYSL4*	4	0.001065296	0.016909828	0.23
662	ENSG00000136378	*ADAMTS7*	4	0.001065296	0.016909828	0.24
663	ENSG00000140931	*CMTM3*	4	0.001065296	0.016909828	0.24
664	ENSG00000166313	*APBB1*	4	0.001065296	0.016909828	0.24
665	ENSG00000025039	*RRAGD*	4	0.001065296	0.016909828	0.25
666	ENSG00000135924	*DNAJB2*	4	0.001065296	0.016909828	0.25
667	ENSG00000137142	*IGFBPL1*	4	0.001065296	0.016909828	0.25
668	ENSG00000143217	*NECTIN4*	4	0.001065296	0.016909828	0.25
669	ENSG00000170390	*DCLK2*	4	0.001065296	0.016909828	0.25
670	ENSG00000176720	*BOK*	4	0.001065296	0.016909828	0.25
671	ENSG00000132321	*IQCA1*	4	0.001065296	0.016909828	0.26
672	ENSG00000155324	*GRAMD2B*	4	0.001065296	0.016909828	0.26
673	ENSG00000166311	*SMPD1*	4	0.001065296	0.016909828	0.26
674	ENSG00000183578	*TNFAIP8L3*	4	0.001065296	0.016909828	0.26
675	ENSG00000185100	*ADSSL1*	4	0.001065296	0.016909828	0.26
676	ENSG00000078725	*BRINP1*	4	0.001065296	0.016909828	0.27
677	ENSG00000117020	*AKT3*	4	0.001065296	0.016909828	0.27
678	ENSG00000125744	*RTN2*	4	0.001065296	0.016909828	0.27
679	ENSG00000171475	*WIPF2*	4	0.001065296	0.016909828	0.27
680	ENSG00000174516	*PELI3*	4	0.001065296	0.016909828	0.28
681	ENSG00000179841	*AKAP5*	4	0.001065296	0.016909828	0.28
682	ENSG00000182919	*C11orf54*	4	0.001065296	0.016909828	0.28
683	ENSG00000196867	*ZFP28*	4	0.001065296	0.016909828	0.28
684	ENSG00000139597	*N4BP2L1*	4	0.001065296	0.016909828	0.29
685	ENSG00000149823	*VPS51*	4	0.001065296	0.016909828	0.29
686	ENSG00000152137	*HSPB8*	4	0.001065296	0.016909828	0.29
687	ENSG00000167771	*RCOR2*	4	0.001065296	0.016909828	0.29
688	ENSG00000183722	*LHFPL6*	4	0.001065296	0.016909828	0.29
689	ENSG00000223953	*C1QTNF5*	4	0.001065296	0.016909828	0.29
690	ENSG00000240849	*TMEM189*	4	0.001065296	0.016909828	0.29
691	ENSG00000277443	*MARCKS*	4	0.001065296	0.016909828	0.29
692	ENSG00000109099	*PMP22*	4	0.001065296	0.016909828	0.3
693	ENSG00000109458	*GAB1*	4	0.001065296	0.016909828	0.3
694	ENSG00000114023	*FAM162A*	4	0.001065296	0.016909828	0.3
695	ENSG00000119408	*NEK6*	4	0.001065296	0.016909828	0.3
696	ENSG00000151882	*CCL28*	4	0.001065296	0.016909828	0.3
697	ENSG00000164086	*DUSP7*	4	0.001065296	0.016909828	0.3
698	ENSG00000170464	*DNAJC18*	4	0.001065296	0.016909828	0.3
699	ENSG00000172663	*TMEM134*	4	0.001065296	0.016909828	0.3
700	ENSG00000176723	*ZNF843*	4	0.001065296	0.016909828	0.3
701	ENSG00000183691	*NOG*	4	0.001065296	0.016909828	0.3
702	ENSG00000198894	*CIPC*	4	0.001065296	0.016909828	0.3
703	ENSG00000077942	*FBLN1*	4	0.001065296	0.016909828	0.31
704	ENSG00000099308	*MAST3*	4	0.001065296	0.016909828	0.31
705	ENSG00000134594	*RAB33A*	4	0.001065296	0.016909828	0.31
706	ENSG00000162522	*KIAA1522*	4	0.001065296	0.016909828	0.31
707	ENSG00000164309	*CMYA5*	4	0.001065296	0.016909828	0.31
708	ENSG00000127334	*DYRK2*	4	0.001065296	0.016909828	0.32
709	ENSG00000128655	*PDE11A*	4	0.001065296	0.016909828	0.32
710	ENSG00000155792	*DEPTOR*	4	0.001065296	0.016909828	0.32
711	ENSG00000169282	*KCNAB1*	4	0.001065296	0.016909828	0.32
712	ENSG00000169992	*NLGN2*	4	0.001065296	0.016909828	0.32
713	ENSG00000174282	*ZBTB4*	4	0.001065296	0.016909828	0.32
714	ENSG00000175356	*SCUBE2*	4	0.001065296	0.016909828	0.32
715	ENSG00000011028	*n_a*	4	0.001065296	0.016909828	0.33
716	ENSG00000107341	*UBE2R2*	4	0.001065296	0.016909828	0.33
717	ENSG00000137801	*THBS1*	4	0.001065296	0.016909828	0.33
718	ENSG00000146122	*DAAM2*	4	0.001065296	0.016909828	0.33
719	ENSG00000158050	*DUSP2*	4	0.001065296	0.016909828	0.33
720	ENSG00000171953	*ATPAF2*	4	0.001065296	0.016909828	0.33
721	ENSG00000124098	*FAM210B*	4	0.001065296	0.016909828	0.34
722	ENSG00000160999	*SH2B2*	4	0.001065296	0.016909828	0.34
723	ENSG00000185338	*SOCS1*	4	0.001065296	0.016909828	0.34
724	ENSG00000243244	*STON1*	4	0.001065296	0.016909828	0.34
725	ENSG00000075624	*ACTB*	4	0.001065296	0.016909828	0.35
726	ENSG00000088543	*C3orf18*	4	0.001065296	0.016909828	0.35
727	ENSG00000114923	*SLC4A3*	4	0.001065296	0.016909828	0.35
728	ENSG00000137409	*MTCH1*	4	0.001065296	0.016909828	0.35
729	ENSG00000152154	*TMEM178A*	4	0.001065296	0.016909828	0.35
730	ENSG00000162104	*ADCY9*	4	0.001065296	0.016909828	0.35
731	ENSG00000133935	*ERG28*	4	0.001065296	0.016909828	0.36
732	ENSG00000137070	*IL11RA*	4	0.001065296	0.016909828	0.36
733	ENSG00000185567	*AHNAK2*	4	0.001065296	0.016909828	0.36
734	ENSG00000203780	*FANK1*	4	0.001065296	0.016909828	0.36
735	ENSG00000134824	*FADS2*	4	0.001065296	0.016909828	0.37
736	ENSG00000160460	*SPTBN4*	4	0.001065296	0.016909828	0.37
737	ENSG00000214402	*LCNL1*	4	0.001065296	0.016909828	0.37
738	ENSG00000113269	*RNF130*	4	0.001065296	0.016909828	0.38
739	ENSG00000114646	*CSPG5*	4	0.001065296	0.016909828	0.38
740	ENSG00000160161	*CILP2*	4	0.001065296	0.016909828	0.38
741	ENSG00000164105	*SAP30*	4	0.001065296	0.016909828	0.38
742	ENSG00000174807	*CD248*	4	0.001065296	0.016909828	0.38
743	ENSG00000235863	*B3GALT4*	4	0.001065296	0.016909828	0.38
744	ENSG00000114450	*GNB4*	4	0.001065296	0.016909828	0.39
745	ENSG00000121753	*ADGRB2*	4	0.001065296	0.016909828	0.39
746	ENSG00000132510	*KDM6B*	4	0.001065296	0.016909828	0.39
747	ENSG00000166444	*ST5*	4	0.001065296	0.016909828	0.39
748	ENSG00000167552	*TUBA1A*	4	0.001065296	0.016909828	0.39
749	ENSG00000173442	*EHBP1L1*	4	0.001065296	0.016909828	0.39

750	ENSG00000178752	*ERFE*	4	0.001065296	0.016909828	0.39
751	ENSG00000100201	*DDX17*	4	0.001065296	0.016909828	0.4
752	ENSG00000115738	*ID2*	4	0.001065296	0.016909828	0.4
753	ENSG00000127663	*KDM4B*	4	0.001065296	0.016909828	0.4
754	ENSG00000198837	*DENND4B*	4	0.001065296	0.016909828	0.4
755	ENSG00000105270	*CLIP3*	4	0.001065296	0.016909828	0.41
756	ENSG00000151376	*ME3*	4	0.001065296	0.016909828	0.41
757	ENSG00000160013	*PTGIR*	4	0.001065296	0.016909828	0.41
758	ENSG00000161921	*CXCL16*	4	0.001065296	0.016909828	0.41
759	ENSG00000166997	*CNPY4*	4	0.001065296	0.016909828	0.41
760	ENSG00000186792	*HYAL3*	4	0.001065296	0.016909828	0.41
761	ENSG00000219607	*PPP1R3G*	4	0.001065296	0.016909828	0.41
762	ENSG00000131943	*C19orf12*	4	0.001065296	0.016909828	0.42
763	ENSG00000143850	*PLEKHA6*	4	0.001065296	0.016909828	0.42
764	ENSG00000050327	*ARHGEF5*	4	0.001065296	0.016909828	0.43
765	ENSG00000072422	*RHOBTB1*	4	0.001065296	0.016909828	0.43
766	ENSG00000119801	*YPEL5*	4	0.001065296	0.016909828	0.43
767	ENSG00000133048	*CHI3L1*	4	0.001065296	0.016909828	0.43
768	ENSG00000165617	*DACT1*	4	0.001065296	0.016909828	0.43
769	ENSG00000168256	*NKIRAS2*	4	0.001065296	0.016909828	0.43
770	ENSG00000168264	*IRF2BP2*	4	0.001065296	0.016909828	0.43
771	ENSG00000095739	*BAMBI*	4	0.001065296	0.016909828	0.44
772	ENSG00000100842	*EFS*	4	0.001065296	0.016909828	0.44
773	ENSG00000105223	*PLD3*	4	0.001065296	0.016909828	0.44
774	ENSG00000109103	*UNC119*	4	0.001065296	0.016909828	0.44
775	ENSG00000113456	*RAD1*	4	0.001065296	0.016909828	0.44
776	ENSG00000143434	*SEMA6C*	4	0.001065296	0.016909828	0.44
777	ENSG00000146373	*RNF217*	4	0.001065296	0.016909828	0.44
778	ENSG00000147912	*FBXO10*	4	0.001065296	0.016909828	0.44
779	ENSG00000166923	*GREM1*	4	0.001065296	0.016909828	0.44
780	ENSG00000004455	*AK2*	4	0.001065296	0.016909828	0.45
781	ENSG00000021762	*OSBPL5*	4	0.001065296	0.016909828	0.45
782	ENSG00000118960	*HS1BP3*	4	0.001065296	0.016909828	0.45
783	ENSG00000125247	*TMTC4*	4	0.001065296	0.016909828	0.45
784	ENSG00000127328	*RAB3IP*	4	0.001065296	0.016909828	0.45
785	ENSG00000130304	*SLC27A1*	4	0.001065296	0.016909828	0.45
786	ENSG00000162627	*SNX7*	4	0.001065296	0.016909828	0.45
787	ENSG00000166484	*MAPK7*	4	0.001065296	0.016909828	0.45
788	ENSG00000105419	*MEIS3*	4	0.001065296	0.016909828	0.46
789	ENSG00000133874	*RNF122*	4	0.001065296	0.016909828	0.46
790	ENSG00000138336	*TET1*	4	0.001065296	0.016909828	0.46
791	ENSG00000181027	*FKRP*	4	0.001065296	0.016909828	0.46
792	ENSG00000076706	*n_a*	4	0.001065296	0.016909828	0.47
793	ENSG00000100380	*ST13*	4	0.001065296	0.016909828	0.47
794	ENSG00000158863	*FAM160B2*	4	0.001065296	0.016909828	0.47
795	ENSG00000186111	*PIP5K1C*	4	0.001065296	0.016909828	0.47
796	ENSG00000196704	*AMZ2*	4	0.001065296	0.016909828	0.47
797	ENSG00000204632	*HLA-G*	4	0.001065296	0.016909828	0.47
798	ENSG00000169105	*CHST14*	4	0.001065296	0.016909828	0.48
799	ENSG00000118816	*CCNI*	4	0.001065296	0.016909828	0.49
800	ENSG00000135362	*PRR5L*	4	0.001065296	0.016909828	0.49
801	ENSG00000146592	*CREB5*	4	0.001065296	0.016909828	0.49
802	ENSG00000174080	*CTSF*	4	0.001065296	0.016909828	0.49
803	ENSG00000270885	*RASL10B*	4	0.001065296	0.016909828	0.49
804	ENSG00000169071	*ROR2*	4	0.001065296	0.016909828	0.5
805	ENSG00000185950	*IRS2*	4	0.001065296	0.016909828	0.5
806	ENSG00000227051	*C14orf132*	4	0.001065296	0.016909828	0.5
807	ENSG00000049769	*PPP1R3F*	4	0.001065296	0.016909828	0.51
808	ENSG00000108389	*MTMR4*	4	0.001065296	0.016909828	0.51
809	ENSG00000167549	*CORO6*	4	0.001065296	0.016909828	0.51
810	ENSG00000184271	*n_a*	4	0.001065296	0.016909828	0.51
811	ENSG00000108950	*FAM20A*	4	0.001065296	0.016909828	0.52
812	ENSG00000112559	*MDFI*	4	0.001065296	0.016909828	0.52
813	ENSG00000167191	*GPRC5B*	4	0.001065296	0.016909828	0.52
814	ENSG00000170581	*STAT2*	4	0.001065296	0.016909828	0.52
815	ENSG00000131446	*MGAT1*	4	0.001065296	0.016909828	0.53
816	ENSG00000158828	*PINK1*	4	0.001065296	0.016909828	0.53
817	ENSG00000198863	*RUNDC1*	4	0.001065296	0.016909828	0.53
818	ENSG00000113328	*CCNG1*	4	0.001065296	0.016909828	0.54
819	ENSG00000197971	*MBP*	4	0.001065296	0.016909828	0.54
820	ENSG00000142065	*ZFP14*	4	0.001065296	0.016909828	0.55
821	ENSG00000151320	*AKAP6*	4	0.001065296	0.016909828	0.55
822	ENSG00000196605	*ZNF846*	4	0.001065296	0.016909828	0.55
823	ENSG00000204219	*TCEA3*	4	0.001065296	0.016909828	0.55
824	ENSG00000062038	*CDH3*	4	0.001065296	0.016909828	0.56
825	ENSG00000100968	*NFATC4*	4	0.001065296	0.016909828	0.56
826	ENSG00000133026	*MYH10*	4	0.001065296	0.016909828	0.56
827	ENSG00000137075	*RNF38*	4	0.001065296	0.016909828	0.56
828	ENSG00000149201	*CCDC81*	4	0.001065296	0.016909828	0.56
829	ENSG00000166482	*MFAP4*	4	0.001065296	0.016909828	0.56
830	ENSG00000046653	*GPM6B*	4	0.001065296	0.016909828	0.57
831	ENSG00000100321	*SYNGR1*	4	0.001065296	0.016909828	0.57
832	ENSG00000144746	*ARL6IP5*	4	0.001065296	0.016909828	0.57
833	ENSG00000196275	*GTF2IRD2*	4	0.001065296	0.016909828	0.57
834	ENSG00000206527	*HACD2*	4	0.001065296	0.016909828	0.57
835	ENSG00000104343	*UBE2W*	4	0.001065296	0.016909828	0.58
836	ENSG00000111052	*LIN7A*	4	0.001065296	0.016909828	0.58
837	ENSG00000140939	*n_a*	4	0.001065296	0.016909828	0.58
838	ENSG00000241852	*C8orf58*	4	0.001065296	0.016909828	0.58
839	ENSG00000065491	*TBC1D22B*	4	0.001065296	0.016909828	0.6
840	ENSG00000110881	*ASIC1*	4	0.001065296	0.016909828	0.6
841	ENSG00000178033	*CALHM5*	4	0.001065296	0.016909828	0.6
842	ENSG00000129250	*KIF1C*	4	0.001065296	0.016909828	0.61
843	ENSG00000146233	*CYP39A1*	4	0.001065296	0.016909828	0.62

844	ENSG00000184206	*GOLGA6L4*	4	0.001065296	0.016909828	0.62
845	ENSG00000139182	*CLSTN3*	4	0.001065296	0.016909828	0.63
846	ENSG00000173898	*SPTBN2*	4	0.001065296	0.016909828	0.63
847	ENSG00000117266	*CDK18*	4	0.001065296	0.016909828	0.64
848	ENSG00000139200	*PIANP*	4	0.001065296	0.016909828	0.64
849	ENSG00000172765	*TMCC1*	4	0.001065296	0.016909828	0.64
850	ENSG00000143847	*PPFIA4*	4	0.001065296	0.016909828	0.65
851	ENSG00000204525	*HLA-C*	4	0.001065296	0.016909828	0.65
852	ENSG00000144810	*COL8A1*	4	0.001065296	0.016909828	0.66
853	ENSG00000182054	*IDH2*	4	0.001065296	0.016909828	0.66
854	ENSG00000161904	*LEMD2*	4	0.001065296	0.016909828	0.67
855	ENSG00000188811	*NHLRC3*	4	0.001065296	0.016909828	0.67
856	ENSG00000112367	*FIG4*	4	0.001065296	0.016909828	0.68
857	ENSG00000036672	*USP2*	4	0.001065296	0.016909828	0.69
858	ENSG00000092295	*TGM1*	4	0.001065296	0.016909828	0.69
859	ENSG00000129467	*ADCY4*	4	0.001065296	0.016909828	0.69
860	ENSG00000173548	*SNX33*	4	0.001065296	0.016909828	0.69
861	ENSG00000197702	*PARVA*	4	0.001065296	0.016909828	0.69
862	ENSG00000213722	*DDAH2*	4	0.001065296	0.016909828	0.69
863	ENSG00000099139	*PCSK5*	4	0.001065296	0.016909828	0.7
864	ENSG00000173376	*NDNF*	4	0.001065296	0.016909828	0.7
865	ENSG00000179476	*C14orf28*	4	0.001065296	0.016909828	0.7
866	ENSG00000100299	*ARSA*	4	0.001065296	0.016909828	0.71
867	ENSG00000127990	*SGCE*	4	0.001065296	0.016909828	0.71
868	ENSG00000162692	*VCAM1*	4	0.001065296	0.016909828	0.72
869	ENSG00000166347	*CYB5A*	4	0.001065296	0.016909828	0.72
870	ENSG00000167772	*ANGPTL4*	4	0.001065296	0.016909828	0.72
871	ENSG00000244242	*IFITM10*	4	0.001065296	0.016909828	0.73
872	ENSG00000159899	*NPR2*	4	0.001065296	0.016909828	0.74
873	ENSG00000169031	*COL4A3*	4	0.001065296	0.016909828	0.74
874	ENSG00000175470	*PPP2R2D*	4	0.001065296	0.016909828	0.76
875	ENSG00000106772	*PRUNE2*	4	0.001065296	0.016909828	0.77
876	ENSG00000148120	*AOPEP*	4	0.001065296	0.016909828	0.77
877	ENSG00000123405	*NFE2*	4	0.001065296	0.016909828	0.78
878	ENSG00000142784	*WDTC1*	4	0.001065296	0.016909828	0.78
879	ENSG00000109846	*CRYAB*	4	0.001065296	0.016909828	0.79
880	ENSG00000146205	*ANO7*	4	0.001065296	0.016909828	0.79
881	ENSG00000067840	*PDZD4*	4	0.001065296	0.016909828	0.81
882	ENSG00000082014	*SMARCD3*	4	0.001065296	0.016909828	0.81
883	ENSG00000145936	*KCNMB1*	4	0.001065296	0.016909828	0.81
884	ENSG00000171045	*TSNARE1*	4	0.001065296	0.016909828	0.82
885	ENSG00000103175	*WFDC1*	4	0.001065296	0.016909828	0.83
886	ENSG00000130707	*ASS1*	4	0.001065296	0.016909828	0.85
887	ENSG00000164930	*FZD6*	4	0.001065296	0.016909828	0.85
888	ENSG00000122971	*ACADS*	4	0.001065296	0.016909828	0.86
889	ENSG00000197879	*MYO1C*	4	0.001065296	0.016909828	0.87
890	ENSG00000143162	*CREG1*	4	0.001065296	0.016909828	0.88
891	ENSG00000164638	*SLC29A4*	4	0.001065296	0.016909828	0.91
892	ENSG00000011275	*RNF216*	4	0.001065296	0.016909828	0.96
893	ENSG00000181035	*SLC25A42*	4	0.001065296	0.016909828	1.03
894	ENSG00000089486	*CDIP1*	4	0.001065296	0.016909828	1.04
895	ENSG00000113504	*SLC12A7*	4	0.001065296	0.016909828	1.04
896	ENSG00000075618	*FSCN1*	4	0.001065296	0.016909828	1.05
897	ENSG00000109072	*VTN*	4	0.001065296	0.016909828	1.07
898	ENSG00000184227	*ACOT1*	4	0.001065296	0.016909828	1.09
899	ENSG00000177106	*EPS8L2*	4	0.001065296	0.016909828	1.1
900	ENSG00000166167	*BTRC*	4	0.001065296	0.016909828	1.13
901	ENSG00000184009	*ACTG1*	4	0.001065296	0.016909828	1.17
902	ENSG00000168434	*COG7*	4	0.001065296	0.016909828	1.21
903	ENSG00000213626	*LBH*	4	0.001065296	0.016909828	1.25
904	ENSG00000122335	*SERAC1*	4	0.001065296	0.016909828	1.3
905	ENSG00000172594	*SMPDL3A*	4	0.001065296	0.016909828	1.39
906	ENSG00000205710	*C17orf107*	4	0.001065296	0.016909828	1.39
907	ENSG00000162734	*PEA15*	4	0.001065296	0.016909828	1.42
908	ENSG00000071246	*VASH1*	4	0.001065296	0.016909828	1.47
909	ENSG00000181788	*SIAH2*	4	0.001065296	0.016909828	1.47
910	ENSG00000182240	*BACE2*	4	0.001065296	0.016909828	1.54
911	ENSG00000067560	*RHOA*	4	0.001065296	0.016909828	1.58
912	ENSG00000084444	*FAM234B*	4	0.001065296	0.016909828	1.79
913	ENSG00000129988	*LBP*	4	0.001065296	0.016909828	1.85
914	ENSG00000131378	*RFTN1*	4	0.001065296	0.016909828	1.86
915	ENSG00000010322	*NISCH*	4	0.001065296	0.016909828	2.08
916	ENSG00000251493	*FOXD1*	4	0.001065296	0.016909828	2.09
917	ENSG00000214517	*PPME1*	4	0.001065296	0.016909828	2.18
918	ENSG00000102466	*FGF14*	4	0.001065296	0.016909828	2.48
919	ENSG00000134343	*ANO3*	4	0.001065296	0.016909828	2.49
920	ENSG00000186314	*PRELID2*	4	0.001065296	0.016909828	2.59
921	ENSG00000168938	*PPIC*	4	0.001065296	0.016909828	2.73
922	ENSG00000183979	*NPB*	4	0.001065296	0.016909828	2.84
923	ENSG00000166165	*CKB*	4	0.001065296	0.016909828	3.02
924	ENSG00000151322	*NPAS3*	4	0.001065296	0.016909828	3.11
925	ENSG00000111640	*GAPDH*	4	0.001065296	0.016909828	3.94
926	ENSG00000135437	*RDH5*	4	0.001065296	0.016909828	3.99
927	ENSG00000147872	*PLIN2*	4	0.001065296	0.016909828	6.74
