# Supplementary material for: Decoding the differentiation of mesenchymal stem cells into mesangial cells at the transcriptomic level
Source: BMC Genomics. 2020 Jul 7;21:467. doi: 10.1186/s12864-020-06868-5 (PMC7339572; doi:10.1186/s12864-020-06868-5)
Supplement: Supplementary file 6 — Additional file 5. Inferences of selected genes to Marbach, TRRUSTv2 and ENCODE TF-Targets dataset. [file 12864_2020_6868_MOESM5_ESM.pdf]

A) Inferences of descending key genes to Marbach, TRRUSTv2 and ENCODE TF-Targets dataset

TF level	TF gene ID	TF gene name	Key gene ID	Key gene name	Marbach2016	TRRUSTv2	ENCODE TF-Targets dataset
1	ENSG00000137309	HMGA1	ENSG00000166825	ANPEP	1	0	0
1	ENSG00000172819	RARG	ENSG00000166825	ANPEP	1	0	0
2	ENSG00000125398	SOX9	ENSG00000166825	ANPEP	1	0	0
1	ENSG00000101412	E2F1	ENSG00000087586	AURKA	1	1	1
1	ENSG00000111206	FOXM1	ENSG00000134057	CCNB1	0	1	1
1	ENSG00000125347	IRF1	ENSG00000134057	CCNB1	1	0	1
1	ENSG00000137309	HMGA1	ENSG00000110092	CCND1	1	0	0
2	ENSG00000001167	NFYA	ENSG00000110092	CCND1	1	0	1
1	ENSG00000101412	E2F1	ENSG00000110092	CCND1	0	1	0
1	ENSG00000129173	E2F8	ENSG00000094804	CDC6	1	0	0
1	ENSG00000173153	ESRRA	ENSG00000094804	CDC6	1	0	0
1	ENSG00000176692	FOXC2	ENSG00000094804	CDC6	1	0	0
1	ENSG00000111206	FOXM1	ENSG00000094804	CDC6	0	1	1
2	ENSG00000114315	HES1	ENSG00000094804	CDC6	1	0	0
2	ENSG00000108788	MLX	ENSG00000094804	CDC6	1	0	0
1	ENSG00000162599	NFIA	ENSG00000094804	CDC6	1	0	0
1	ENSG00000121068	TBX2	ENSG00000094804	CDC6	1	0	0
1	ENSG00000101412	E2F1	ENSG00000170312	CDK1	0	1	1
1	ENSG00000153234	NR4A2	ENSG00000170312	CDK1	1	0	0
1	ENSG00000134532	SOX5	ENSG00000163938	GNL3	1	0	0
1	ENSG00000179388	EGR3	ENSG00000128342	LIF	1	0	0
1	ENSG00000173153	ESRRA	ENSG00000128342	LIF	1	0	0
1	ENSG00000106511	MEOX2	ENSG00000128342	LIF	1	0	0
1	ENSG00000153234	NR4A2	ENSG00000128342	LIF	1	0	0

B) Inferences of ascending key genes to Marbach, TRRUSTv2 and ENCODE TF-Targets dataset

TF level	TF gene ID	TF gene name	Key gene ID	Key gene name	Marbach2016	TRRUSTv2	ENCODE TF-Targets dataset
7	ENSG00000123405	NFE2	ENSG00000107796	ACTA2	1	0	0
8	ENSG00000112658	SRF	ENSG00000107796	ACTA2	1	0	1
8	ENSG00000126561	STAT5A	ENSG00000107796	ACTA2	1	0	1
8	ENSG00000148737	TCF7L2	ENSG00000107796	ACTA2	1	0	1
8	ENSG00000010030	ETV7	ENSG00000107796	ACTA2	1	0	0
8	ENSG00000005073	HOXA11	ENSG00000107796	ACTA2	1	0	0
8	ENSG00000007866	TEAD3	ENSG00000107796	ACTA2	1	0	0
8	ENSG00000112182	BACH2	ENSG00000115461	IGFBP5	1	0	0
8	ENSG00000253293	HOXA10	ENSG00000115461	IGFBP5	1	0	0
8	ENSG00000147862	NFIB	ENSG00000115461	IGFBP5	1	0	0
8	ENSG00000184271	POU6F1	ENSG00000115461	IGFBP5	1	0	0
8	ENSG00000165804	ZNF219	ENSG00000115461	IGFBP5	1	0	0
8	ENSG00000139083	ETV6	ENSG00000115461	IGFBP5	0	1	0
8	ENSG00000150907	FOXO1	ENSG00000077943	ITGA8	1	0	0
8	ENSG00000165804	ZNF219	ENSG00000140545	MFGE8	1	0	0
8	ENSG00000168610	STAT3	ENSG00000100345	MYH9	1	0	1
8	ENSG00000150907	FOXO1	ENSG00000100345	MYH9	1	0	0
8	ENSG00000118689	FOXO3	ENSG00000100345	MYH9	1	0	0
8	ENSG00000172201	ID4	ENSG00000100345	MYH9	1	0	0
8	ENSG00000124766	SOX4	ENSG00000100345	MYH9	1	0	0
7	ENSG00000123405	NFE2	ENSG00000101605	MYOM1	1	0	0
7	ENSG00000164920	OSR2	ENSG00000101605	MYOM1	1	0	0
8	ENSG00000146592	CREB5	ENSG00000101605	MYOM1	1	0	0
8	ENSG00000170370	EMX2	ENSG00000101605	MYOM1	1	0	0
8	ENSG00000139083	ETV6	ENSG00000101605	MYOM1	1	0	0
8	ENSG00000172201	ID4	ENSG00000101605	MYOM1	1	0	0
8	ENSG00000164093	PITX2	ENSG00000101605	MYOM1	1	0	0
8	ENSG00000148737	TCF7L2	ENSG00000101605	MYOM1	1	0	0
8	ENSG00000007866	TEAD3	ENSG00000101605	MYOM1	1	0	0
9	ENSG00000106031	HOXA13	ENSG00000101605	MYOM1	1	0	0
9	ENSG00000186350	RXRA	ENSG00000101605	MYOM1	1	0	0
9	ENSG00000152284	TCF7L1	ENSG00000101605	MYOM1	1	0	0
6	ENSG00000105866	SP4	ENSG00000113721	PDGFRB	1	0	0
7	ENSG00000184481	FOXO4	ENSG00000113721	PDGFRB	1	0	0
7	ENSG00000120149	MSX2	ENSG00000113721	PDGFRB	1	0	0
8	ENSG00000178573	MAF	ENSG00000113721	PDGFRB	1	0	0
8	ENSG00000178665	ZNF713	ENSG00000113721	PDGFRB	1	0	0
8	ENSG00000143437	ARNT	ENSG00000124212	PTGIS	1	0	0
8	ENSG00000139083	ETV6	ENSG00000124212	PTGIS	1	0	0
8	ENSG00000178573	MAF	ENSG00000124212	PTGIS	1	0	0
8	ENSG00000147862	NFIB	ENSG00000124212	PTGIS	1	0	0
8	ENSG00000184271	POU6F1	ENSG00000124212	PTGIS	1	0	0
8	ENSG00000159216	RUNX1	ENSG00000124212	PTGIS	1	0	0
8	ENSG00000007866	TEAD3	ENSG00000124212	PTGIS	1	0	0
9	ENSG00000186350	RXRA	ENSG00000124212	PTGIS	1	0	0
7	ENSG00000123405	NFE2	ENSG00000068976	PYGM	1	0	0
8	ENSG00000131759	RARA	ENSG00000068976	PYGM	1	0	0
8	ENSG00000112658	SRF	ENSG00000068976	PYGM	1	0	0
7	ENSG00000123405	NFE2	ENSG00000135919	SERPINE2	1	0	0
8	ENSG00000105419	MEIS3	ENSG00000135919	SERPINE2	1	0	0
9	ENSG00000176842	IRX5	ENSG00000135919	SERPINE2	1	0	0
9	ENSG00000152284	TCF7L1	ENSG00000135919	SERPINE2	1	0	0
8	ENSG00000007866	TEAD3	ENSG00000149591	TAGLN	1	0	0
8	ENSG00000112658	SRF	ENSG00000149591	TAGLN	1	1	1

C) Inferences of vascular smooth muscle contraction related genes to Marbach, TRRUSTv2 and ENCODE TF-Targets dataset

TF level	TF gene ID	TF gene name	Target gene ID	Target gene name	Marbach2016	TRRUSTv2	ENCODE TF-Targets dataset
8	ENSG00000221869	CEBPD	ENSG00000072952	MRVI1	1	0	0
7	ENSG00000184481	FOXO4	ENSG00000072952	MRVI1	1	0	0
8	ENSG00000178573	MAF	ENSG00000072952	MRVI1	1	0	0
8	ENSG00000164093	PITX2	ENSG00000072952	MRVI1	1	0	0
8	ENSG00000124766	SOX4	ENSG00000072952	MRVI1	1	0	0
8	ENSG00000112658	SRF	ENSG00000072952	MRVI1	1	0	1
9	ENSG00000152284	TCF7L1	ENSG00000072952	MRVI1	1	0	0
8	ENSG00000148737	TCF7L2	ENSG00000072952	MRVI1	1	0	0
8	ENSG00000112182	BACH2	ENSG00000077157	PPP1R12B	1	0	0
8	ENSG00000007866	TEAD3	ENSG00000077157	PPP1R12B	1	0	0
8	ENSG00000126351	THRA	ENSG00000077157	PPP1R12B	1	0	0
8	ENSG00000010030	ETV7	ENSG00000101335	MYL9	1	0	0
8	ENSG00000112658	SRF	ENSG00000101335	MYL9	1	1	1
8	ENSG00000010030	ETV7	ENSG00000107796	ACTA2	1	0	0
8	ENSG00000005073	HOXA11	ENSG00000107796	ACTA2	1	0	0
7	ENSG00000123405	NFE2	ENSG00000107796	ACTA2	1	0	0
8	ENSG00000112658	SRF	ENSG00000107796	ACTA2	1	0	1
8	ENSG00000126561	STAT5A	ENSG00000107796	ACTA2	1	0	1
8	ENSG00000148737	TCF7L2	ENSG00000107796	ACTA2	1	0	1
8	ENSG00000007866	TEAD3	ENSG00000107796	ACTA2	1	0	0
8	ENSG00000172216	CEBPB	ENSG00000145936	KCNMB1	1	0	0
8	ENSG00000140044	JDP2	ENSG00000145936	KCNMB1	1	0	0
8	ENSG00000131759	RARA	ENSG00000145936	KCNMB1	1	0	0
8	ENSG00000112658	SRF	ENSG00000145936	KCNMB1	1	0	1
8	ENSG00000170265	ZNF282	ENSG00000145936	KCNMB1	1	0	0
8	ENSG00000054598	FOXC1	ENSG00000151067	CACNA1C	1	0	0
9	ENSG00000106031	HOXA13	ENSG00000151067	CACNA1C	1	0	0
8	ENSG00000165030	NFIL3	ENSG00000151067	CACNA1C	1	0	0
8	ENSG00000164093	PITX2	ENSG00000151067	CACNA1C	1	0	0
8	ENSG00000143190	POU2F1	ENSG00000151067	CACNA1C	1	0	0
8	ENSG00000126351	THRA	ENSG00000151067	CACNA1C	1	0	0
8	ENSG00000151090	THRB	ENSG00000151067	CACNA1C	1	0	0
7	ENSG00000131668	BARX1	ENSG00000160013	PTGIR	1	0	0
8	ENSG00000010030	ETV7	ENSG00000160013	PTGIR	1	0	0
8	ENSG00000131759	RARA	ENSG00000160013	PTGIR	1	0	0
8	ENSG00000170265	ZNF282	ENSG00000160013	PTGIR	1	0	0
8	ENSG00000139083	ETV6	ENSG00000167641	PPP1R14A	1	0	0
8	ENSG00000124766	SOX4	ENSG00000167641	PPP1R14A	1	0	0
8	ENSG00000112658	SRF	ENSG00000167641	PPP1R14A	1	0	0
9	ENSG00000152284	TCF7L1	ENSG00000173175	ADCY5	1	0	0
