# Supplementary material for: Decoding the differentiation of mesenchymal stem cells into mesangial cells at the transcriptomic level
Source: BMC Genomics. 2020 Jul 7;21:467. doi: 10.1186/s12864-020-06868-5 (PMC7339572; doi:10.1186/s12864-020-06868-5)
Supplement: Supplementary file 7 — Additional file 6. List of enriched pathways in each level. [file 12864_2020_6868_MOESM6_ESM.pdf]

Full list of enriched KEGG pathways in each level FDR < 0.05

L1	L2	L3	L4	L5	L6	L7	L8	L9	KEGG	Pathway name
1	1	0	0	0	0	0	0	0	hsa00970	Aminoacyl-tRNA biosynthesis
1	1	0	0	0	0	0	0	0	hsa03410	Base excision repair
1	1	0	0	0	0	0	0	0	hsa03030	DNA replication
1	1	0	0	0	0	0	0	0	hsa03460	Fanconi anemia pathway
1	1	0	0	0	0	0	0	0	hsa03440	Homologous recombination
1	1	0	0	0	0	0	0	0	hsa04114	Oocyte meiosis
1	1	0	0	0	0	0	0	0	hsa00230	Purine metabolism
1	1	1	0	0	0	0	0	0	hsa04110	Cell cycle
1	1	1	0	0	0	0	0	0	hsa03430	Mismatch repair
1	1	1	0	0	0	0	0	0	hsa03420	Nucleotide excision repair
1	1	1	0	0	0	0	0	0	hsa00240	Pyrimidine metabolism
1	1	1	0	0	0	0	0	0	hsa03008	Ribosome biogenesis in eukaryotes
1	1	1	1	0	0	0	0	0	hsa03050	Proteasome
1	1	1	1	0	0	0	0	0	hsa03013	RNA transport
1	1	1	1	0	0	0	0	0	hsa03040	Spliceosome
0	1	0	0	0	0	0	0	0	hsa04914	Progesterone-mediated oocyte maturation
0	1	1	0	0	0	0	0	0	hsa04210	Apoptosis
0	1	1	1	0	0	0	0	0	hsa03022	Basal transcription factors
0	1	1	1	0	0	0	0	0	hsa03015	mRNA surveillance pathway
0	1	1	1	0	0	0	0	0	hsa03018	RNA degradation
0	1	1	1	1	1	1	0	0	hsa01100	Metabolic pathways
0	0	1	0	0	0	0	0	0	hsa00510	N-Glycan biosynthesis
0	0	1	1	0	0	0	0	0	hsa01130	Biosynthesis of antibiotics
0	0	1	1	0	0	0	0	0	hsa00310	Lysine degradation
0	0	1	1	1	0	0	0	0	hsa04141	Protein processing in endoplasmic reticulum
0	0	1	1	1	0	0	0	0	hsa03020	RNA polymerase
0	0	1	1	1	0	0	0	0	hsa04120	Ubiquitin mediated proteolysis
0	0	0	1	0	0	0	0	0	hsa05231	Choline metabolism in cancer
0	0	0	1	0	0	0	0	0	hsa00020	Citrate cycle (TCA cycle)
0	0	0	1	0	0	0	0	0	hsa00563	Glycosylphosphatidylinositol(GPI)-anchor biosynthesis
0	0	0	1	0	0	0	0	0	hsa04146	Peroxisome
0	0	0	1	0	0	0	0	0	hsa04070	Phosphatidylinositol signaling system
0	0	0	1	0	0	0	0	0	hsa03060	Protein export
0	0	0	1	0	0	0	0	0	hsa05211	Renal cell carcinoma
0	0	0	1	1	0	0	0	0	hsa05100	Bacterial invasion of epithelial cells
0	0	0	1	1	0	0	0	0	hsa05120	Epithelial cell signaling in Helicobacter pylori infection
0	0	0	1	1	0	0	0	0	hsa05131	Shigellosis
0	0	0	1	1	1	0	0	0	hsa04722	Neurotrophin signaling pathway
0	0	0	1	1	1	0	0	0	hsa05016	Huntington's disease
0	0	0	1	1	1	1	0	0	hsa05010	Alzheimer's disease
0	0	0	1	1	1	1	0	0	hsa05012	Parkinson's disease
0	0	0	1	1	1	1	0	0	hsa04932	Non-alcoholic fatty liver disease (NAFLD)
0	0	0	1	1	1	1	0	0	hsa00190	Oxidative phosphorylation
0	0	0	0	1	1	1	0	0	hsa04142	Lysosome
0	0	0	0	1	1	1	1	0	hsa03010	Ribosome
0	0	0	0	0	0	1	0	0	hsa04520	Adherens junction
0	0	0	0	0	0	1	0	0	hsa04360	Axon guidance
0	0	0	0	0	0	1	0	0	hsa00531	Glycosaminoglycan degradation
0	0	0	0	0	0	1	1	0	hsa04270	Vascular smooth muscle contraction
0	0	0	0	0	0	1	1	0	hsa04512	ECM-receptor interaction
0	0	0	0	0	0	1	1	0	hsa04510	Focal adhesion
0	0	0	0	0	0	1	1	0	hsa04066	HIF-1 signaling pathway
0	0	0	0	0	0	1	1	0	hsa04910	Insulin signaling pathway
0	0	0	0	0	0	1	1	0	hsa00511	Other glycan degradation
0	0	0	0	0	0	1	1	0	hsa04921	Oxytocin signaling pathway
0	0	0	0	0	0	1	1	0	hsa04145	Phagosome
0	0	0	0	0	0	1	1	0	hsa04611	Platelet activation
0	0	0	0	0	0	1	1	0	hsa04015	Rap1 signaling pathway
0	0	0	0	0	0	1	1	0	hsa04810	Regulation of actin cytoskeleton
0	0	0	0	0	0	1	1	1	hsa04514	Cell adhesion molecules (CAMs)
0	0	0	0	0	0	1	1	1	hsa05414	Dilated cardiomyopathy
0	0	0	0	0	0	1	1	1	hsa05410	Hypertrophic cardiomyopathy (HCM)
0	0	0	0	0	0	0	1	0	hsa04152	AMPK signaling pathway
0	0	0	0	0	0	0	1	0	hsa01040	Biosynthesis of unsaturated fatty acids
0	0	0	0	0	0	0	1	0	hsa04068	FoxO signaling pathway
0	0	0	0	0	0	0	1	0	hsa04670	Leukocyte transendothelial migration
0	0	0	0	0	0	0	1	0	hsa04010	MAPK signaling pathway
0	0	0	0	0	0	0	1	0	hsa04530	Tight junction
0	0	0	0	0	0	0	1	1	hsa05412	Arrhythmogenic right ventricular cardiomyopathy (ARVC)

Term	Pathway name	P Value	FDR (Benjamini-Hochberg method)	Level
hsa00970	Aminoacyl-tRNA biosynthesis	5.69E-04	0.01152069	L1
hsa03410	Base excision repair	1.68E-04	0.003990091	L1
hsa04110	Cell cycle	6.88E-15	1.96E-12	L1
hsa03030	DNA replication	2.70E-14	3.86E-12	L1
hsa03460	Fanconi anemia pathway	1.93E-07	9.16E-06	L1
hsa03440	Homologous recombination	1.46E-04	0.003787921	L1
hsa03430	Mismatch repair	4.11E-05	0.001301931	L1
hsa03420	Nucleotide excision repair	1.02E-04	0.002907191	L1
hsa04114	Oocyte meiosis	3.14E-05	0.001116311	L1
hsa03050	Proteasome	4.90E-04	0.01068541	L1
hsa00230	Purine metabolism	9.29E-04	0.017510916	L1
hsa00240	Pyrimidine metabolism	1.02E-07	5.81E-06	L1
hsa03008	Ribosome biogenesis in eukaryotes	5.35E-08	3.81E-06	L1
hsa03013	RNA transport	2.55E-06	1.04E-04	L1
hsa03040	Spliceosome	6.83E-10	6.49E-08	L1
hsa04110	Cell cycle	4.97E-15	1.44E-12	L2
hsa03030	DNA replication	1.73E-14	1.67E-12	L2
hsa03460	Fanconi anemia pathway	6.57E-10	3.80E-08	L2
hsa03440	Homologous recombination	3.14E-05	6.48E-04	L2
hsa03430	Mismatch repair	2.53E-07	7.32E-06	L2
hsa03420	Nucleotide excision repair	2.60E-07	6.84E-06	L2
hsa04114	Oocyte meiosis	1.27E-07	4.60E-06	L2
hsa04914	Progesterone-mediated oocyte maturation	0.001946419	0.026456265	L2
hsa03050	Proteasome	2.00E-07	6.42E-06	L2
hsa00230	Purine metabolism	8.73E-05	0.001681189	L2
hsa00240	Pyrimidine metabolism	8.74E-09	3.61E-07	L2
hsa03008	Ribosome biogenesis in eukaryotes	7.06E-10	3.40E-08	L2
hsa03018	RNA degradation	7.63E-06	1.70E-04	L2
hsa03013	RNA transport	2.70E-12	1.95E-10	L2
hsa03040	Spliceosome	7.95E-15	1.16E-12	L2
hsa00970	Aminoacyl-tRNA biosynthesis	0.00163523	0.02337098	L2
hsa04210	Apoptosis	5.39E-04	0.008612076	L2
hsa03022	Basal transcription factors	3.22E-04	0.005459108	L2
hsa03410	Base excision repair	2.75E-04	0.004950544	L2
hsa01100	Metabolic pathways	8.73E-04	0.01319934	L2
hsa03015	mRNA surveillance pathway	4.87E-06	1.17E-04	L2
hsa04210	Apoptosis	2.30E-04	0.006659435	L3
hsa03022	Basal transcription factors	4.37E-07	4.22E-05	L3
hsa01130	Biosynthesis of antibiotics	3.85E-04	0.010096169	L3
hsa00310	Lysine degradation	0.001366431	0.030042252	L3
hsa00510	N-Glycan biosynthesis	0.001432656	0.029261075	L3
hsa04110	Cell cycle	0.003164692	0.049785528	L3
hsa01100	Metabolic pathways	2.52E-05	0.001042067	L3
hsa03430	Mismatch repair	0.002053433	0.038961223	L3
hsa03015	mRNA surveillance pathway	2.11E-06	1.22E-04	L3
hsa03420	Nucleotide excision repair	7.66E-05	0.00246381	L3
hsa03050	Proteasome	1.97E-05	9.52E-04	L3
hsa04141	Protein processing in endoplasmic reticulum	3.98E-04	0.009576996	L3
hsa00240	Pyrimidine metabolism	0.002297159	0.040827068	L3
hsa03008	Ribosome biogenesis in eukaryotes	1.48E-06	1.08E-04	L3
hsa03020	RNA polymerase	0.002339763	0.03917248	L3
hsa03013	RNA transport	2.26E-09	3.28E-07	L3
hsa03040	Spliceosome	4.82E-14	1.40E-11	L3
hsa04120	Ubiquitin mediated proteolysis	4.27E-05	0.001545895	L3
hsa05100	Bacterial invasion of epithelial cells	1.69E-06	7.06E-05	L4
hsa05231	Choline metabolism in cancer	0.001288296	0.019683387	L4
hsa00020	Citrate cycle (TCA cycle)	0.003642659	0.040290759	L4
hsa04144	Endocytosis	4.77E-09	1.40E-06	L4
hsa05120	Epithelial cell signaling in Helicobacter pylori infection	4.56E-04	0.007395635	L4
hsa00563	Glycosylphosphatidylinositol(GPI)-anchor biosynthesis	0.00526481	0.048668216	L4
hsa03015	mRNA surveillance pathway	8.83E-08	6.47E-06	L4
hsa04146	Peroxisome	0.004477181	0.045870329	L4
hsa04070	Phosphatidylinositol signaling system	0.002826704	0.035418297	L4
hsa03060	Protein export	0.002176061	0.028595923	L4
hsa04141	Protein processing in endoplasmic reticulum	5.33E-08	5.21E-06	L4
hsa05211	Renal cell carcinoma	0.004527505	0.044812171	L4
hsa03020	RNA polymerase	4.83E-05	0.001087215	L4
hsa05131	Shigellosis	2.15E-05	5.25E-04	L4
hsa04071	Sphingolipid signaling pathway	0.001780124	0.024552655	L4
hsa05010	Alzheimer's disease	6.25E-06	1.66E-04	L4
hsa03022	Basal transcription factors	0.003160244	0.037905316	L4

hsa01130	Biosynthesis of antibiotics	0.00134308	0.019496785	L4
hsa05016	Huntington's disease	1.05E-08	1.54E-06	L4
hsa00310	Lysine degradation	0.003737926	0.039824708	L4
hsa01100	Metabolic pathways	1.75E-06	6.40E-05	L4
hsa04722	Neurotrophin signaling pathway	0.00332709	0.038305555	L4
hsa04932	Non-alcoholic fatty liver disease (NAFLD)	2.30E-04	0.003959359	L4
hsa00190	Oxidative phosphorylation	3.70E-06	1.08E-04	L4
hsa05012	Parkinson's disease	3.06E-06	9.97E-05	L4
hsa03050	Proteasome	8.02E-05	0.001564515	L4
hsa05205	Proteoglycans in cancer	0.005150172	0.049179507	L4
hsa03018	RNA degradation	8.11E-05	0.00148374	L4
hsa03013	RNA transport	1.52E-06	7.44E-05	L4
hsa03040	Spliceosome	8.89E-08	5.21E-06	L4
hsa04120	Ubiquitin mediated proteolysis	5.11E-05	0.001069003	L4
hsa04120	Ubiquitin mediated proteolysis	9.10E-09	6.57E-07	L5
hsa05010	Alzheimer's disease	1.72E-06	4.96E-05	L5
hsa05100	Bacterial invasion of epithelial cells	0.00257882	0.045569164	L5
hsa04144	Endocytosis	2.58E-08	1.49E-06	L5
hsa05120	Epithelial cell signaling in Helicobacter pylori infection	0.002105935	0.049504073	L5
hsa05016	Huntington's disease	7.54E-08	3.63E-06	L5
hsa04142	Lysosome	1.97E-04	0.005164404	L5
hsa01100	Metabolic pathways	5.31E-07	1.92E-05	L5
hsa04722	Neurotrophin signaling pathway	0.002537554	0.047773485	L5
hsa04932	Non-alcoholic fatty liver disease (NAFLD)	1.54E-06	4.94E-05	L5
hsa00190	Oxidative phosphorylation	7.95E-15	2.31E-12	L5
hsa05012	Parkinson's disease	2.14E-09	2.07E-07	L5
hsa04141	Protein processing in endoplasmic reticulum	4.19E-07	1.73E-05	L5
hsa03010	Ribosome	7.29E-11	1.05E-08	L5
hsa03020	RNA polymerase	0.002157884	0.046888369	L5
hsa05131	Shigellosis	0.002288203	0.046188462	L5
hsa05010	Alzheimer's disease	3.92E-11	3.81E-09	L6
hsa05016	Huntington's disease	4.33E-10	2.53E-08	L6
hsa01100	Metabolic pathways	3.61E-09	1.76E-07	L6
hsa04722	Neurotrophin signaling pathway	8.73E-05	0.002546359	L6
hsa04932	Non-alcoholic fatty liver disease (NAFLD)	3.04E-07	1.27E-05	L6
hsa00190	Oxidative phosphorylation	4.06E-16	6.48E-14	L6
hsa05012	Parkinson's disease	2.45E-10	1.79E-08	L6
hsa03010	Ribosome	3.61E-33	1.05E-30	L6
hsa04144	Endocytosis	7.17E-07	2.62E-05	L6
hsa04142	Lysosome	5.14E-05	0.00166602	L6
hsa04071	Sphingolipid signaling pathway	0.001478141	0.038505988	L6
hsa04520	Adherens junction	0.00369944	0.042507928	L7
hsa04360	Axon guidance	0.003091287	0.038673623	L7
hsa04512	ECM-receptor interaction	3.25E-05	0.001191119	L7
hsa00531	Glycosaminoglycan degradation	0.002910087	0.038069976	L7
hsa04066	HIF-1 signaling pathway	2.02E-04	0.004909463	L7
hsa04142	Lysosome	4.39E-06	3.22E-04	L7
hsa00511	Other glycan degradation	0.001593658	0.024295485	L7
hsa04145	Phagosome	3.00E-04	0.006263096	L7
hsa05205	Proteoglycans in cancer	0.003260011	0.039080186	L7
hsa04810	Regulation of actin cytoskeleton	2.29E-05	9.60E-04	L7
hsa05010	Alzheimer's disease	6.75E-05	0.002194021	L7
hsa04514	Cell adhesion molecules (CAMs)	2.09E-04	0.004697069	L7
hsa05414	Dilated cardiomyopathy	4.46E-04	0.008133322	L7
hsa04144	Endocytosis	1.51E-06	2.21E-04	L7
hsa04510	Focal adhesion	1.93E-05	9.43E-04	L7
hsa05410	Hypertrophic cardiomyopathy (HCM)	9.97E-04	0.017049157	L7
hsa04910	Insulin signaling pathway	0.001208265	0.019487375	L7
hsa01100	Metabolic pathways	1.51E-04	0.004026929	L7
hsa04932	Non-alcoholic fatty liver disease (NAFLD)	1.10E-05	6.45E-04	L7
hsa00190	Oxidative phosphorylation	2.03E-06	1.98E-04	L7
hsa04921	Oxytocin signaling pathway	0.001840064	0.025369573	L7
hsa05012	Parkinson's disease	2.09E-04	0.004697069	L7
hsa04611	Platelet activation	0.001638173	0.02373276	L7
hsa04015	Rap1 signaling pathway	1.43E-04	0.004169934	L7
hsa03010	Ribosome	8.38E-23	2.46E-20	L7
hsa04270	Vascular smooth muscle contraction	4.03E-04	0.007845315	L7
hsa04152	AMPK signaling pathway	0.002764207	0.037123199	L8
hsa01040	Biosynthesis of unsaturated fatty acids	0.001392946	0.026317766	L8
hsa04514	Cell adhesion molecules (CAMs)	4.76E-06	3.41E-04	L8
hsa05414	Dilated cardiomyopathy	7.91E-06	4.54E-04	L8
hsa04510	Focal adhesion	1.05E-06	3.02E-04	L8

hsa04068	FoxO signaling pathway	0.003916002	0.047781571	L8
hsa05410	Hypertrophic cardiomyopathy (HCM)	1.13E-06	1.61E-04	L8
hsa04910	Insulin signaling pathway	2.63E-04	0.00628188	L8
hsa04670	Leukocyte transendothelial migration	0.002985044	0.038248806	L8
hsa04010	MAPK signaling pathway	7.33E-04	0.014926529	L8
hsa04921	Oxytocin signaling pathway	1.15E-05	5.51E-04	L8
hsa04611	Platelet activation	1.27E-04	0.004052049	L8
hsa04015	Rap1 signaling pathway	2.26E-06	2.17E-04	L8
hsa04530	Tight junction	0.001735084	0.028892142	L8
hsa04270	Vascular smooth muscle contraction	2.33E-04	0.00605894	L8
hsa05412	Arrhythmogenic right ventricular cardiomyopathy (ARVC)	9.12E-05	0.003267151	L8
hsa04512	ECM-receptor interaction	1.33E-04	0.003807655	L8
hsa04144	Endocytosis	5.05E-04	0.011090252	L8
hsa04066	HIF-1 signaling pathway	0.002032358	0.030263152	L8
hsa00511	Other glycan degradation	0.002604038	0.036725324	L8
hsa04145	Phagosome	0.00177796	0.027975061	L8
hsa04810	Regulation of actin cytoskeleton	3.98E-05	0.001630149	L8
hsa03010	Ribosome	0.001498573	0.026542218	L8
hsa05412	Arrhythmogenic right ventricular cardiomyopathy (ARVC)	1.53E-05	0.002036824	L9
hsa04514	Cell adhesion molecules (CAMs)	2.84E-06	7.58E-04	L9
hsa05414	Dilated cardiomyopathy	2.39E-04	0.015839575	L9
hsa05410	Hypertrophic cardiomyopathy (HCM)	1.01E-04	0.008910191	L9
