# Supplementary material for: Decoding the differentiation of mesenchymal stem cells into mesangial cells at the transcriptomic level
Source: BMC Genomics. 2020 Jul 7;21:467. doi: 10.1186/s12864-020-06868-5 (PMC7339572; doi:10.1186/s12864-020-06868-5)
Supplement: Supplementary file 8 — Additional file 8. TO-GCN robustness analysis. [file 12864_2020_6868_MOESM8_ESM.pdf]

TO-GCN robustness analysis

In order to demonstrate the robustness of TO-GCN, we tested the level order stability by using 7 different TF genes with DE ≤ 4 and co-expressed with *FOSL1* (PCC > 0.99) as new initial nodes to construct the corresponding TO-GCNs. We calculated the differences in level number for each TF gene against the original TO-GCN: the TO-GCN constructed with *FOSL1* as initial node.

The results showed that on average 5.5% of TFs in the original TO-GCN were assigned to a different level (Table 1). However, the average and standard deviation of the overall level change for each new TO-GCN with a different initial node is very small (Table 1). This indicated that the new ordered TO-GCNs are very similar to the original TO-GCN that was constructed with *FOSL1*.

Table 1. Statistics of level order changes with different initial seeds

TF Gene ID of initial seed	DE value	Gene Name	Proportion of TFs with Level Change	Mean of Level Change [a]	STDEV of Level Change
ENSG00000111206	1	*FOXM1*	0.34%	0.00	0.06
ENSG00000137309	1	*HMGA1*	7.72%	0.04	0.27
ENSG00000137310	2	*TCF19*	6.13%	0.05	0.24
ENSG00000176692	2	*FOXC2*	7.14%	0.04	0.26
ENSG00000115163	3	*CENPA*	7.39%	0.05	0.27
ENSG00000007968	4	*E2F2*	6.21%	-0.04	0.25
ENSG00000115816	4	*CEBPZ*	3.61%	0.03	0.19

a. For each node, the positive and negative number represented the respective level changes to lower or higher level; in new TO-GCN against the original TO-GCN that was constructed with *FOSL1*. Zero represents no change between two TO-GCNs. For example, if a node belonging to level 5 in original TO-GCN has changed to level 4 in the new TO-GCN, the level change value will be -1.
